# Synthesis and Antifungal Evaluation of Isoquinoline‐3‐amide Derivatives Containing a Phenoxypyridine Moiety for Plant Protection

**DOI:** 10.1002/cbdv.71635

**Published:** 2026-09-10

**Authors:** Jiayao Zhang, Shuaipeng Sun, Yajing Guo, Zhiyuan Xu, Jing Zhang, Gang Feng, Haixin Ding, Qiang Xiao

**Affiliations:** ^1^ Jiangxi Provincial Key Laboratory of Organic Functional Molecules Institute of Organic Chemistry Jiangxi Science and Technology Normal University Nanchang China; ^2^ Environment and Plant Protection Institute Chinese Academy of Tropical Agricultural Science Haikou China

**Keywords:** isoquinoline amides, molecular docking, phenoxypyridine, plant fungicide, SDHI

## Abstract

To discover novel green antifungal candidates, 20 3‐acylaminoisoquinoline derivatives were designed and synthesized by introducing the active fragment phenoxypyridine. Their structures were verified by ^1^H NMR, ^13^C NMR, and HRMS. The structure of compound **X9** was further confirmed via X‐ray crystallography. Their antifungal activities against five phytopathogenic fungi were evaluated to develop isoquinoline‐based antifungal agents. Among them, compound **X6** demonstrated significant antifungal activity against *Botrytis cinerea* with an EC_50_ value of 7.55 µg/mL. *In vivo* assays revealed that compound **X6** exhibited strong protective and curative efficacy against *B. cinerea* with rates of 77.86% and 45.45% at a concentration of 100 µg/mL. Molecular docking studies confirmed that isoquinoline amide compounds possess strong binding affinity to succinate dehydrogenase (SDH). Molecular dynamics (MD) simulations revealed that compound **X6** has better pocket spatial compatibility and more stable binding interactions with SDH than boscalid. Furthermore, SDH activity assays showed compound **X6** inhibited SDH activity by 66.7% at 4 EC_50_ concentration, which was comparable to that of the boscalid (75.85%). These findings demonstrated that isoquinoline derivatives incorporating the phenoxypyridine fragment possess potent fungicidal properties for crop safety, with compound **X6** emerging as a promising candidate for a novel SDH inhibitor.

## Graphical abstract

The splicing of phenoxypyridine moieties to isoquinolin‐3‐carboxylic acid enhanced its biological activity against plant pathogenic fungi. Among target compounds, compound **X6** demonstrated significant antifungal activity against *Botrytis cinerea* *in vitro* and *in vivo* assays, Molecular docking, molecular dynamics simulations, as well as SDH enzyme assay, have further verified that compound **X6** may be a promising candidate for novel SDH inhibitors.

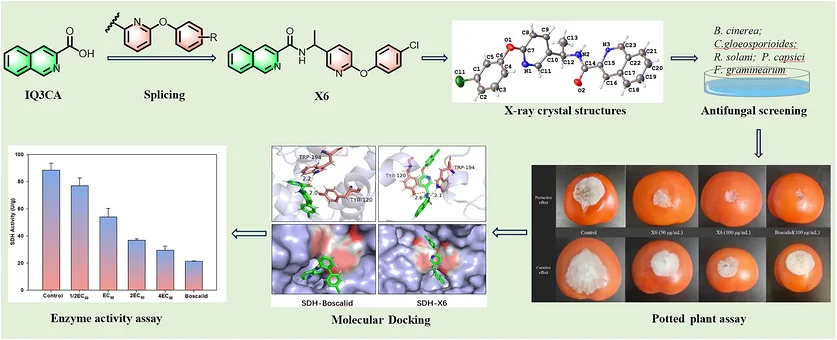

## 1 Introduction

The development of candidate pesticide molecules featuring novel skeletons, environmental friendliness, and low toxicity to non‐target organisms has always been a hot topic in pesticide chemistry [1, 2, 3]. An active fragment is the key component of chemical drug structure. To find effective active fragments and create new pesticides by using intermediate derivatization method or active substructure splicing is beneficial to pesticide innovation and reduce research costs [4, 5]. Among the active fragments, diphenyl ether or phenoxypyridine structures show excellent, broad‐spectrum and potent biological activity [6, 7, 8].

**FIGURE 1:**
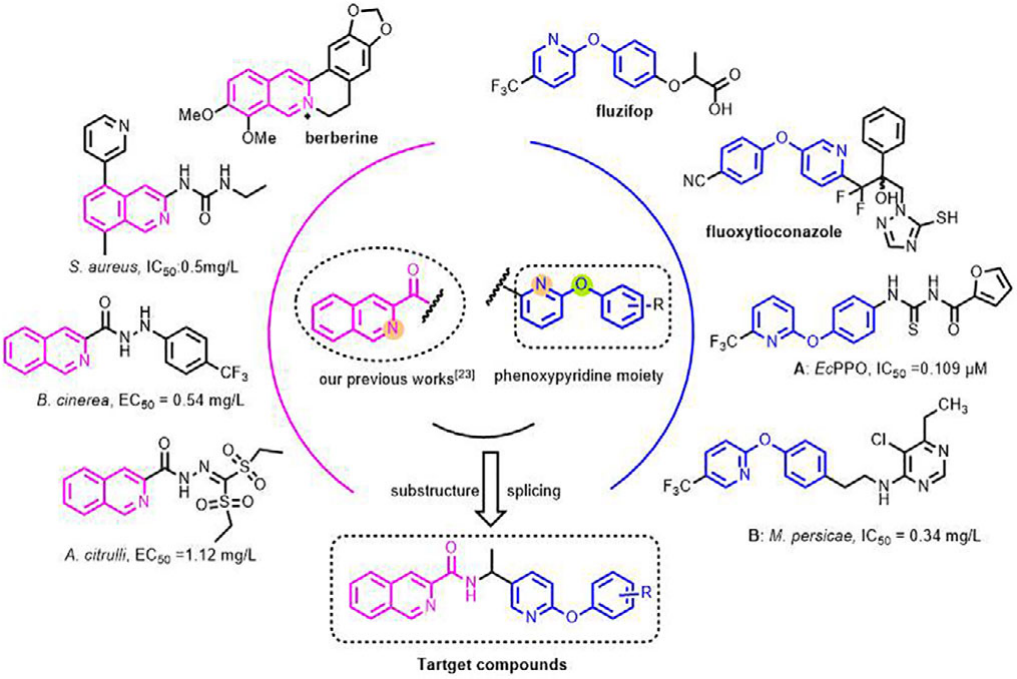
Design strategy of target compounds in this work.

Compared with the diphenyl ether structure, the pyridine ring in the structure of phenoxypyridine is substituted by the benzene ring. In addition to having good lipid solubility, metabolic stability, and cell membrane penetration, it can also increase the probability of π–π stacking of target molecules and show more excellent biological activity [7]. A variety of pesticide molecules containing the phenoxypyridine fragments have been developed and play an important role in crop protection research (Figure 1). Using diclofop as the lead compound and replacing 2,4‐dichlorophenyl with trifluoromethylpyridine, the highly active acetyl CoA carboxylase (ACC) inhibitor fluazifop was obtained. Subsequently, extensive research on herbicides containing phenylpyridine was developed [9]. Zhao et al. synthesized a novel protoporphyrinogen oxidase (PPO) inhibitor A, which demonstrated superior inhibitory activity compared to oxyfluorfen containing the structure of diphenyl ether [10]. The compounds containing the phenoxypyridine moiety also exhibit significant insecticidal and fungicidal activity. Using phenoxypyridine as an intermediate to couple a pyrimidine group, the IC_50_ value of compound **B** against peach aphid (*Myzus persicae*) was 0.34 mg/L, which was much lower than that of pythiodifon and imidacloprid [11]. The phenoxypyridine derivatives containing chalcone have shown excellent antiviral activity against TMW, which had a strong binding affinity for TMV‐CP [12].

Based on natural products to find the active skeleton, it is one of the effective ways to realize the innovation of small molecule green pesticides. Isoquinoline compounds, as vital constituents of alkaloids, exhibit a wide range of biological activities and pharmacological effects, including antitumor, antiviral, anti‐inflammatory, antifungal, antimalarial, and other activities [13, 14, 15, 16]. Through the simplification and derivatization of isoquinoline scaffold, alkynyl isoquinolines [17], isoquinoline‐3‐oxazoline amides and isoquinoline‐3‐hydrazides [18] have shown strong antibacterial and antifungal activities. In the previous screening of antibacterial activities, our group found that isoquinoline‐3‐carboxylic acid (IQ3CA) has excellent broad‐spectrum bactericidal and fungicidal activity against a variety of plant pathogens [19]. The active substructure splicing is a highly efficient strategy in pesticide development. This approach involves linking two or more molecular fragments with specific biological activities via chemical bonds to obtain novel compounds that exhibit multiple advantages or enhanced activity [20, 21, 22]. By splicing the active substructure disulfone, we obtained an isoquinoline disulfone compound which has excellent bactericidal effect on plant bacterial diseases [23]. Therefore, isoquinoline‐3‐carboxylic acid, as a promising lead compound, was selected for further structure–activity studies. In this paper, a series of isoquinoline‐3‐amide derivatives containing phenoxypyridine moieties (**X1**‐**X20**) were designed and synthesized, characterized by NMR and HRMS. Through antifungal activity testing and structure–activity relationship analysis, we aim to identify more effective compounds and develop fungicidal agents based on the isoquinoline skeleton structure.

## 2 Results and Discussions

### 2.1 Chemical Synthesis

**SCHEME 1:**
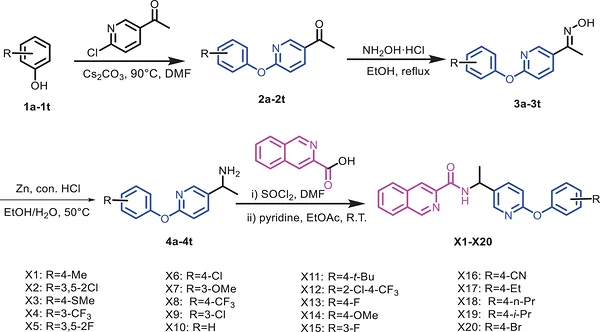
Synthesis of target compounds **X1**−**X20**.

The synthesis of isoquinoline‐3‐amide derivatives containing phenoxypyridine fragments is depicted in Scheme 1. Using IQ3CA as starting material, phenoxypyridine moiety was introduced at its 3‐carboxyl position based on the principle of substructure splicing. The phenoxypyridine fragment was synthesized by a three‐step chemical reaction. Under the catalysis of cesium carbonate, benzene phenols with different substituents were coupled with 2‐chloro‐5‐acetylpyridine to synthesize compound **2a**–**2t**. The synthesized oximes **3a**–**3t** was reduced using zinc powder and hydrochloric acid to obtain the intermediate **4a**–**4t** bearing an amino functional group. Concurrently, isoquinoline‐3‐carboxylic acid underwent acylation to generate acyl chloride intermediate. The acyl chloride intermediate was then coupled with compound **4a**–**4t** in the presence of pyridine as a base to afford the desired target compound **X1–X20**. All target compounds were purified by column chromatography or recrystallization, and their structures were confirmed by ^1^H NMR, ^13^C NMR, and high‐resolution mass spectrometry (HRMS).

### 2.2 Crystal Structure of Compound X9

**FIGURE 2:**
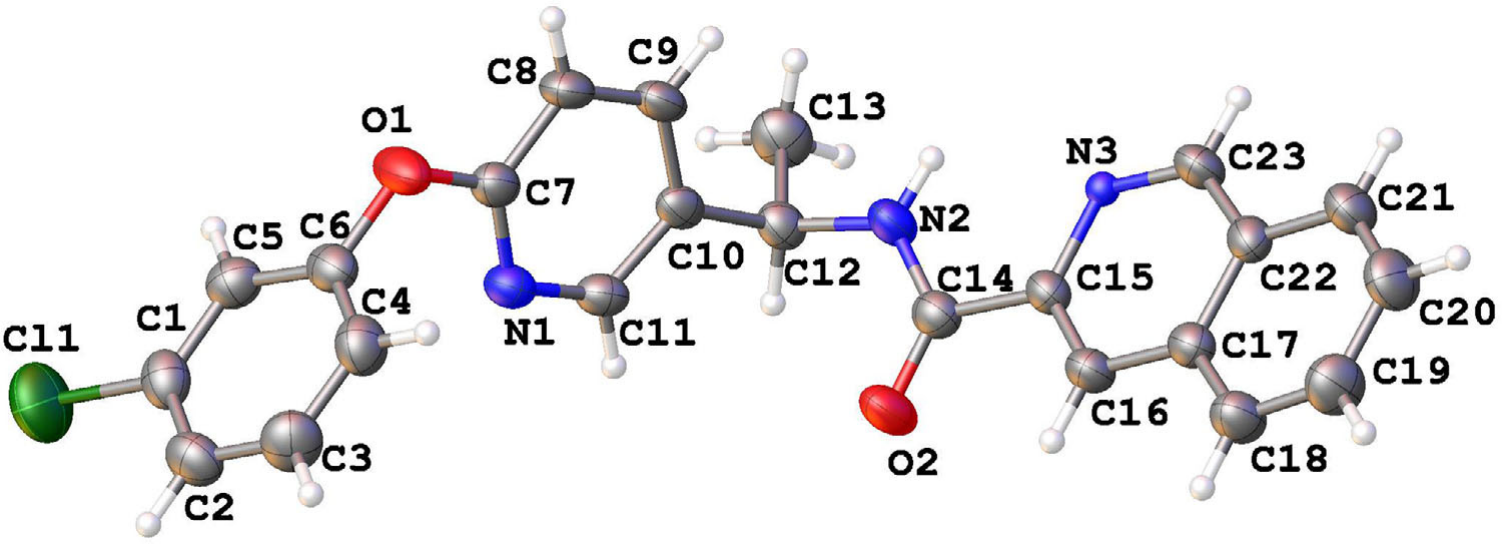
X‐ray crystal structures of compound **X9**.

As shown in Figure 2, the structure of compound **X9** has been further confirmed by X‐ray crystallographic diffraction analysis, and the detailed crystallographic data have been deposited at the Cambridge Crystallographic Data Centre (CCDC number: 2499347). The isoquinoline and phenoxypyridine moieties were linked by the C(14)─N(2)─C(12) bond. The dihedral angles between the isoquinoline ring and the pyridine, phenyl rings are 85.5° and 3.8°, respectively. Two intramolecular hydrogen bonds N2─H2⋯N3 and C9─H9⋯O2 are formed within a unit cell, resulting in a more compact packing structure. The crystallographic data could be acquired from Tables S2–S4.

### 2.3 *In Vitro* Antifungal Activity and SAR Analysis

The *in vitro* antifungal activities of all synthesized compounds against five phytopathogenic fungi were primarily evaluated at concentration of 50 µg/mL (Table S1) and 100 µg/mL. Boscalid was selected as the positive control agent. The results of the primary screening as shown in Table 1 indicated that some target compounds exhibited good antifungal activities against *Botrytis cinerea*, *Colletotrichum gloeosporioides*, and *Rhizoctonia solani*. For *Phytophthora capsici* and *Fusarium graminearum*, the target compounds showed weak inhibitory effects (inhibition rate < 60%). Among the target compounds, compounds **X6** and **X16** exhibited remarkable antifungal activity against *B. cinerea*, with inhibition rate more than 80%, which was similar to that of boscalid. Compounds **X6**, **X14**, and **X16** exhibited strong activity against *C. gloeosporioides* with inhibition of 71.73%, 69.57%, and 80.27%, superior to boscalid (56.51%). To explore the relationship between the chemical structure of the target compounds and their antifungal efficacy, the structure–activity relationship (SAR) was analyzed. We found that the ─*t*Bu group dramatically reduced activity compared to the ─Et group, indicating that bulky groups at this position are detrimental due to the restricted space of the hydrophobic pocket. We have specifically compared F, Cl, and Br. The order of activity was found to be Cl > Br > F (inhibition rate, 4‐Cl 84.32%, 4‐F 34.83%). This trend suggests that polarizability plays a more important role than electronegativity here, as Cl exhibit optimal halogen‐bonding interactions in the active site. Furthermore, we found that the inhibition effect of *ortho*‐substituents on benzene rings was greater than that of *meta*‐substituents, especially the electron‐withdrawing groups (EWG) at 4‐position showed significantly improved activity, among which chlorine and cyano groups at 4‐position were the best. This may be attributed to the fact the para position allows the EWG to exert maximum resonance electron withdrawal on the aromatic ring.

**TABLE 1:** *In vitro* antifungal activity of target compounds against five plant pathogenic fungi at 100 µg/mL.

Compound	Inhibition rate (%)^a^				
	*B. cinerea*	*P. capsici*	*C. gloeosporioides*	*R. solani*	*F. graminearum*
X1	36.13 ± 0.80	18.19 ± 2.0	30.82 ±2.53	45.01 ±1.36	36.13 ± 2.11
X2	41.07 ± 2.12	16.11 ± 0.46	49.17 ± 4.40	24.29 ± 3.75	46.22 ± 1.87
X3	76.92 ± 3.03	16.37 ± 0.44	45.55 ± 3.96	64.02 ± 0.68	37.86 ± 1.55
X4	35.35 ± 0.80	35.93 ± 1.15	40.82 ± 3.61	55.47 ± 2.45	36.9 ± 0.86
X5	37.66 ± 0.92	24.14 ± 1.87	46.66 ± 1.52	63.65 ± 0.68	27.63 ± 2.61
X6	84.32 ± 0.46	24.99 ± 4.86	71.73 ± 0.42	74.02 ± 1.04	53.45 ± 1.29
X7	47.59 ± 0.46	25.82 ± 3.05	34.59 ± 1.93	58.37 ± 3.12	43.42 ± 3.41
X8	41.08 ± 1.22	45.71 ± 0.87	42.08 ± 0.42	66.52 ± 1.96	32.05 ± 1.14
X9	38.55 ± 2.12	11.37 ± 0.44	60.34 ± 1.11	58.38 ± 1.71	5.95 ± 3.41
X10	36.49 ± 1.67	40.65 ± 0.87	41.68 ± 0.84	63.63 ± 1.42	28.21 ± 0.43
X11	20.22 ± 3.03	44.42 ± 0.76	19.59 ± 1.52	53.58 ± 2.58	33.33 ± 1.29
X12	36.23 ± 4.47	25.26 ± 1.88	26.37 ± 1.84	26.53 ± 1.18	42.86 ± 1.52
X13	34.83 ± 0.8	18.95 ± 2.31	35.27 ± 1.52	37.79 ± 3.88	13.1 ± 2.61
X14	47.2 ± 2.88	41.55 ± 1.60	69.57 ± 1.11	29.15 ± 3.53	23.81 ± 4.23
X15	49.06 ± 2.31	23.31 ± 1.57	44.58 ± 0.84	56.96 ± 0.49	57.95 ± 1.14
X16	80.25 ±1.85	18.43 ± 0.76	80.27 ± 2.19	64.02 ± 0.79	33.33 ± 1.87
X17	48.3 ± 2.31	8.53 ± 1.07	49.65 ± 0.73	47.54 ± 2.19	37.52 ± 1.69
X18	37.88 ±1.60	7.51 ± 0.76	44.17 ± 3.11	24.86 ± 3.49	34.36 ± 1.26
X19	44.21 ± 0.8	19.87 ± 2.31	38.76 ± 1.93	44.01 ± 2.89	30.38 ± 1.52
X20	38.21 ± 3.23	22.01 ± 1.15	35.42 ± 2.53	33.94 ± 1.18	24.36 ± 1.47
Boscalid	85.91 ± 0.46	51.71 ±1.57	56.51 ± 2.92	97.12 ± 1.04	56.31 ± 1.29

^a^ Values are the mean ± standard deviation (SD) of three replicates.

The target compounds exhibiting over 60% inhibition rate were selected for EC_50_ values determination. As shown in Table 2, compounds **X6** displayed significant antifungal activity against *B. cinerea* with EC_50_ values of 7.55 µg/mL, which was comparable to that of the positive control boscalid (EC_50_ = 5.39 µg/mL). For *C. gloeosporioides*, compounds **X6**, **X9**, **X14**, and **X16** displayed significant growth inhibition with EC_50_ values of 11.92, 23.12, 14.16, and 10.49 µg/mL, respectively, which are more potent than boscalid (26.17 µg/mL).

**TABLE 2:** EC_50_ values of high‐activity compounds against three pathogenic fungi.

Fungi	Compd.	Regression equation	*r*	EC_50_ (µg/mL)	95% CI^a^
*B. cinerea*	X3	3.4187 + 1.3177x	0.9929	15.85	10.10–24.87
	X6	3.9346 + 1.2135x	0.967	7.55	4.29–13.29
	X16	3.5936 + 1.2680x	0.9634	12.86	8.62–19.18
	Boscalid	4.0541 + 1.2931x	0.9899	5.39	2.78–10.47
*C. gloeosporioides*	X6	4.0213 + 0.9095x	0.9824	11.92	6.99–20.32
	X9	3.7966 + 0.8822x	0.9914	23.12	12.51–42.72
	X14	3.8050 + 1.0383x	0.971	14.16	8.87–22.60
	X16	3.4613 + 1.5745x	0.9894	10.49	6.71–13.42
	Boscalid	3.8153 + 0.8356x	0.9892	26.17	14.78–46.32
*R. solani*	X3	3.3076 + 1.0449x	0.9907	41.66	23.42‐74.08
	X5	3.8081 + 0.8958x	0.9891	21.41	11.37–40.30
	X6	3.8805 + 0.9489x	0.9924	15.13	7.55–30.32
	X8	3.5956 + 0.9431x	0.9974	30.84	17.19–55.34
	X16	3.1910 + 1.0180x	0.9892	59.84	29.91–119.72
	Boscalid	4.5513 + 1.3980x	0.9968	2.09	1.31–3.34

^a^ Confidence interval.

### 2.4 *In Vivo* Antifungal Activity

**FIGURE 3:**
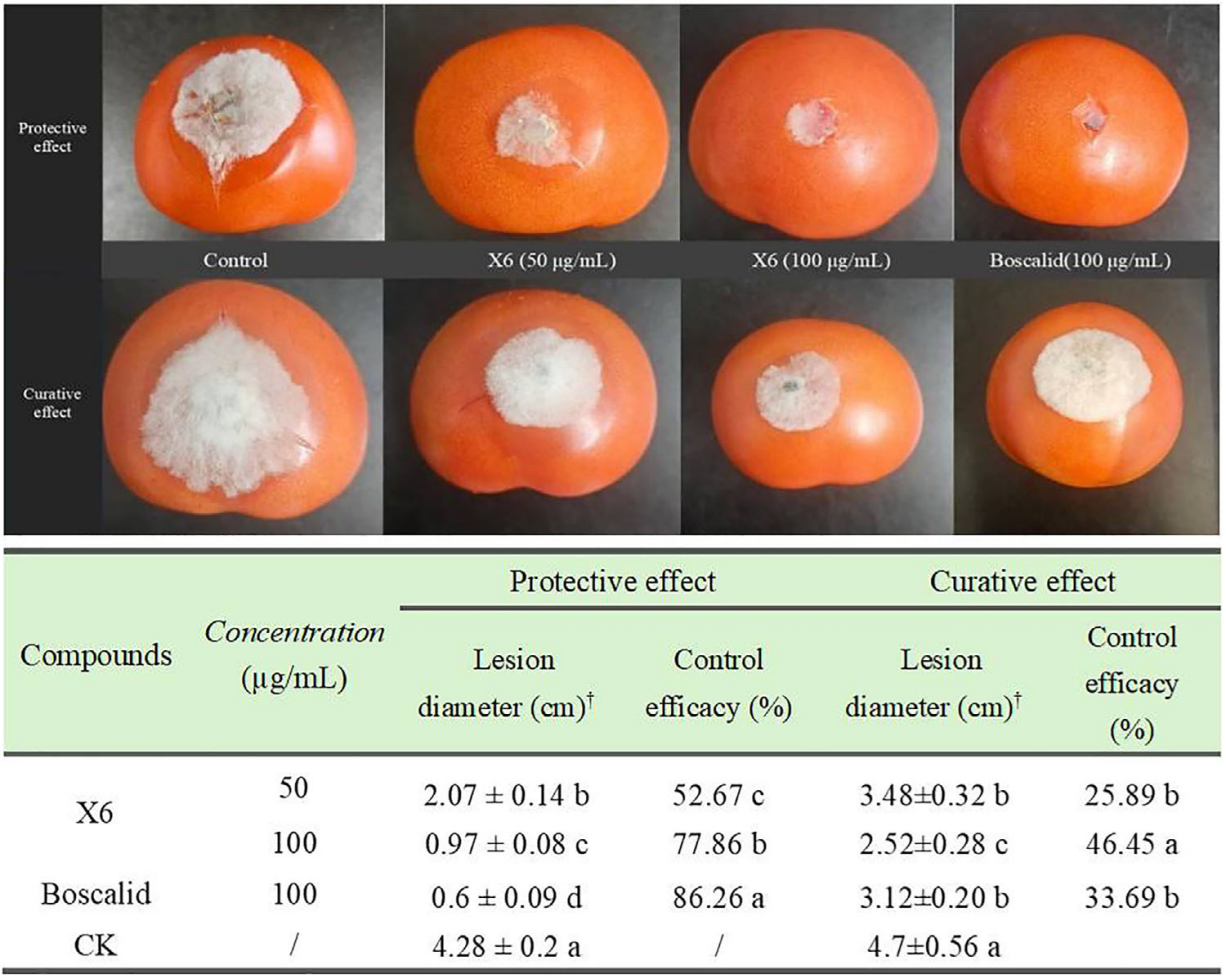
*In vivo* protective and curative effect of compound **X6** and boscalid against *B. cinerea* on tomato fruits. †: Values are the mean ± standard deviation (SD) of three replicates. Different letters were significantly different (*p* < 0.05) through a Duncan's test.

With the acquired encouraging antifungal activity of isoquinoline‐3‐amide derivatives containing phenoxypyridine fragments, compound **X6** with satisfactory EC_50_ values against *B. cinerea* were selected for further evaluation of its *in vivo* protective and curative efficacy on tomato fruits. The results were shown in Figure 3. Compound **X6** showed a strong protective effect with an efficacy of 77.86% at the concentration of 100 µg/mL, which was weaker than that of the control agent boscalid (86.26%). However, in the curative activities test, the efficacy of compound X6 was 46.45% at the same concentration, which was better than that of the control agent boscalid (33.69%). These results revealed that phenoxypyridine combined with isoquinoline compound can improve potent fungicidal potency in crop protection.

### 2.5 Scanning Electron Microscopy (SEM) Observations

**FIGURE 4:**
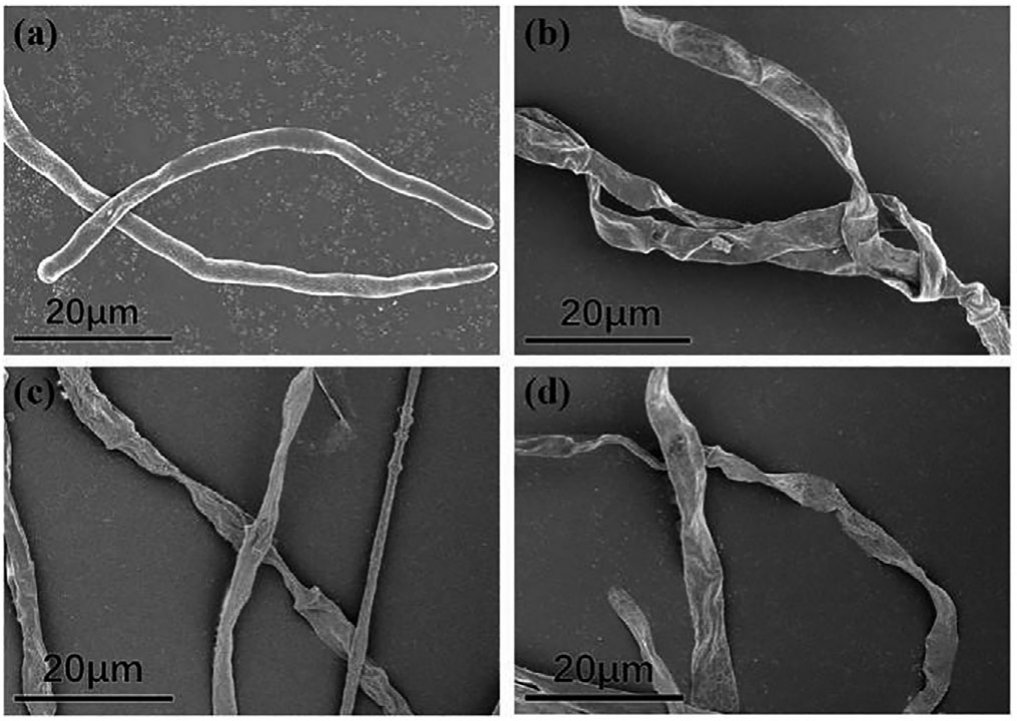
SEM images of *B. cinerea* treated with DMSO (a), boscalid (b) and **X6** (c and d).

To elucidate the effective pathway of compound **X6** against *B. cinerea*, the SEM was used to observe the effect of compound **X6** on mycelium morphology. As shown in Figure 4, after treatment with compound **X6** at concentrations of 2EC_50_ value and 4EC_50_ value for 48 h, the tested mycelial collapsed and shriveled, which was different from that of the blank control group. It was seen that compound **X6** could effectively destroy the mycelial structure.

### 2.6 Molecular Docking

Currently, reported commercialized SDHI (succinate dehydrogenase inhibitor) compounds adhere to a classical structural model comprising an aromatic heterocyclic carboxylic acid core, an amide linkage, and a hydrophobic tail chain [24, 25, 26]. Our designed compound structure precisely conforms to this model, leading us to hypothesize that its active moiety may exert a fungicidal mechanism with potential inhibitory effects on SDH. To elucidate the structural basis underlying the differential antifungal activity of isoquinoline amides, we performed molecular docking analyses using the cryo‐electron microscopy structure of succinate dehydrogenase (SDH) from *Saccharomyces cerevisiae* [27]. This SDH structure exhibits a high degree of homology with pathogenic fungi, supporting its utility in the rational design of next‐generation fungicides.

**FIGURE 5:**
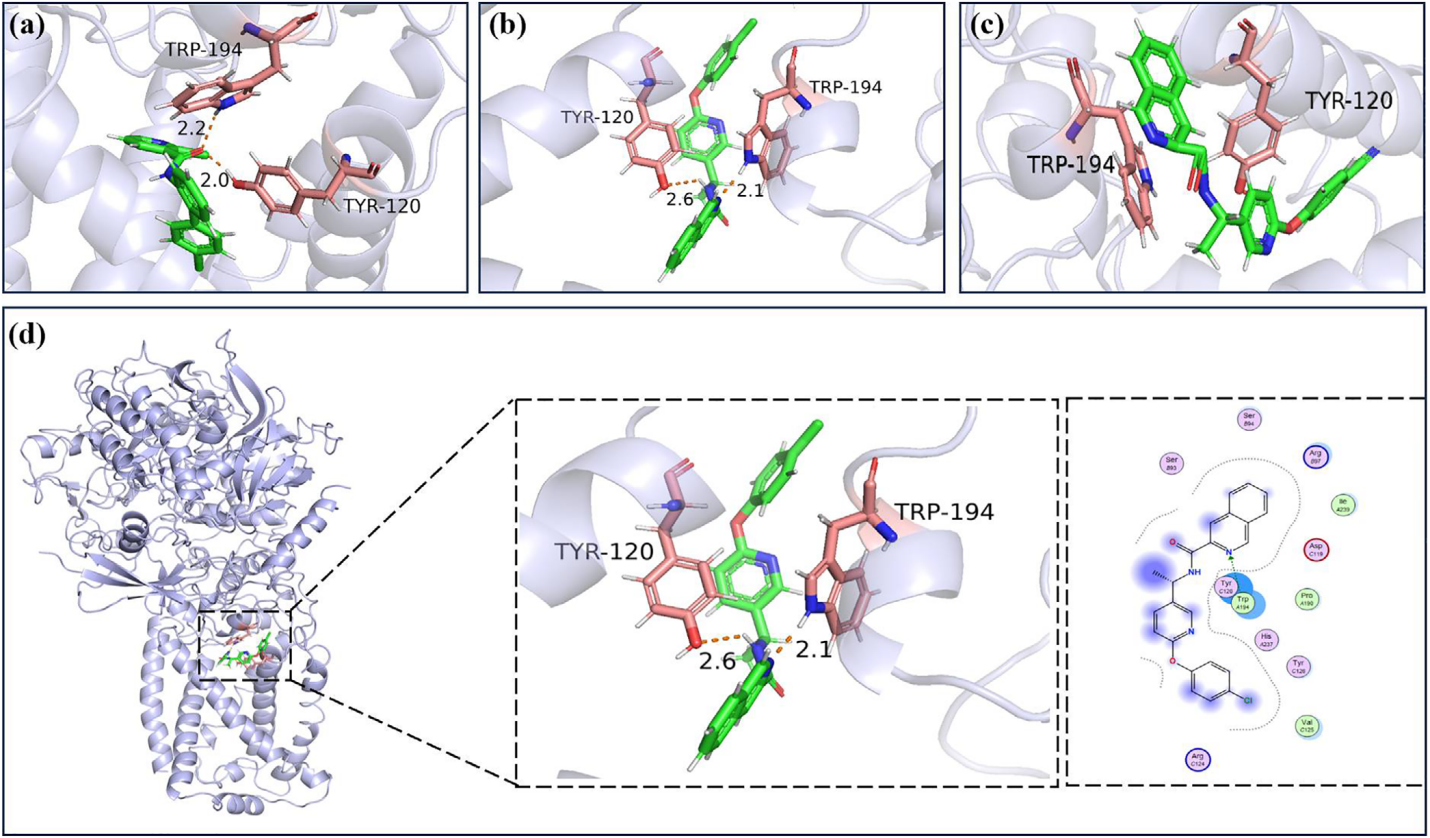
The molecular docking of compounds boscalid (a), **X6** (b and d), and **X16** (c) with SDH.

Binding energy analysis showed that the binding energies of **X6** and **X16** with SDH were −6.67 and −5.63 kcal/mol, respectively. Both values are lower than that of the positive control boscalid (−5.33 kcal/mol), indicating that **X6** and X**16** have stronger binding affinity for the target protein. In terms of interaction patterns, hydrogen bonding is the main interaction mode between the **X6** compound and SDH. As depicted in Figure 5, boscalid and **X6** each formed two hydrogen bonds with TRP‐194 and TYR‐120 on the protein; however, **X16** did not form hydrogen bonds, and it is speculated that its interactions with TRP‐194 and TYR‐120 are mainly through aromatic ring and C–H group interactions. It is noteworthy that although both **X6** and boscalid formed two hydrogen bonds, the binding energy of **X6** is significantly lower than that of boscalid. This is because, although the **X6** molecule binds in the same pocket as boscalid, the binding sites are different, which may cause the hydrogen bond interactions between boscalid and TRP‐194/TYR‐120 to be less stable compared to those with **X6**. Therefore, it can be inferred that TRP‐194 and TYR‐120 may play a more critical role in stabilizing the ligand–protein complex. However, this inference still requires further experimental verification.

### 2.7 Molecular Dynamic Simulation

**FIGURE 6:**
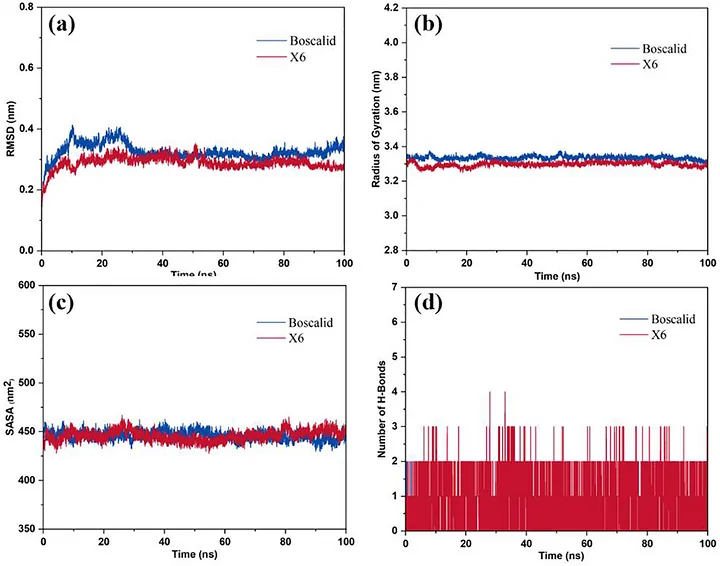
100 ns MD analysis of Boscalid– and X6–protein complexes. (a) Complex RMSD in MD. (b) Complex Rg in MD. (c) Complex SASA in MD. (d) Number of H‐bonds.

Molecular dynamics (MD) simulations revealed distinct differences in binding stability between **X6–**protein and boscalid–protein complexes. First, as shown in the RMSD profile (Figure 6a), the **X6** complex rapidly reached thermodynamic equilibrium within the first 20 ns, with ligand RMSD stabilizing at 0.3 nm and remaining consistent throughout the simulation. In contrast, the boscalid complex exhibited biphasic behavior: severe conformational fluctuations in the initial 30 ns, followed by a short stable period, and a continuous RMSD increase in the final 20 ns, indicating that stable binding was not achieved within 100 ns. Furthermore, hydrogen bond analysis (Figure 6d) showed that **X6** formed an average of two stable hydrogen bonds with key active‐site residues during the entire simulation, maintaining stable binding via polar interactions. By contrast, boscalid translocated from the original binding pocket to an interhelical region after 30 ns, with its six‐membered ring exposed to solvent and lacking stable hydrogen‐bonding constraints, which likely accounts for its unstable binding. Moreover, the radius of gyration (Rg ≈ 3.30 nm) and solvent‐accessible surface area (SASA ≈ 450 nm^2^) of both complexes remained nearly constant (Figure 6b,c). These results further verified that the increased RMSD of the boscalid system originated exclusively from local ligand displacement, rather than global structural variation of the target protein. To further evaluate the binding affinity difference, MM‐GBSA binding free energy calculation was performed based on the last 10 ns of equilibrated trajectories. Energy decomposition indicated that binding was driven mainly by van der Waals interactions, with weak contributions from electrostatic interactions. The total binding free energy of **X6** was −47.05 kcal mol^−^^1^, significantly more favorable than that of boscalid (−35.19 kcal mol^−^^1^) (Figure S1). In conclusion, **X6** exhibits better pocket spatial compatibility and more stable binding interactions, thereby achieving higher target affinity than boscalid.

### 2.8 SDH Enzyme Inhibition Assay

**FIGURE 7:**
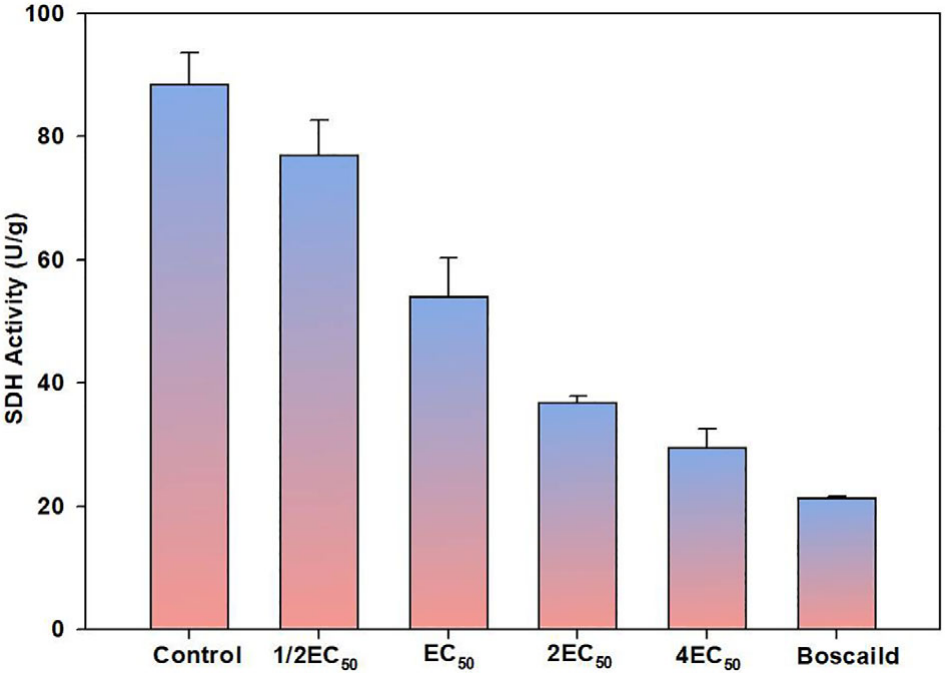
SDH activity under different treatment concentrations.

To further verify whether succinate dehydrogenase (SDH) is a potential target of compound **X6**, the activity of the extracted crude enzyme from *B. cinerea* was determined using a SDH Activity Assay Kit. The results showed that compound **X6** significantly inhibited SDH activity in a concentration‐dependent manner (Figure 7). The inhibition rate of SDH activity reached 38.93% at the EC_50_ concentration and increased to 66.7% at 4 EC_50_, which was comparable to that of the positive control boscalid (75.85%) at the same concentration (30.2 µg/mL). These results aligned with in vitro antifungal activity, indicating that SDH is likely one of the key target enzymes of compound **X6** against *B. cinerea*.

## 3 Materials and Methods

### 3.1 Equipment and Materials

Unless otherwise specified, all the reagents were acquired from commercial sources and used without further drying or purification. Silica gel GF_254_ and column chromatography silica gel for isolation (200−300 mesh) were both purchased from Qingdao Haiyang Chemical Co. Ltd. All of the ^1^H and ^13^C nuclear magnetic resonance (NMR) spectra were recorded on Bruker Avance DPX 400 MHz spectrometer with CDCl_3_ or DMSO‐*d*_6_ as solvent. High‐resolution mass spectrometry (HRMS) was recorded on a Waters Xevo G3 QTOF. X‐ray diffraction analysis was performed on a Bruker D8 VENTURE system. SEM (SU8600, Hitachi) was used to observe the morphology of fungi.

Five phytopathogenic fungi, *B. cinerea*, *F. graminearum*, *R. solani*, *P. capsici*, and *C. gloeosporioides* were provided and stored by the plant pathology laboratory of Hainan Environment and Plant Protection Institute. Fresh tomato fruits (*Solanum lycopersicum* L.) were obtained from a commercial market in Longhua District, Haikou, Hainan, China.

### 3.2 Chemical Synthesis

#### 3.2.1 General Procedure for Synthesis of Compounds **2a–2t**

A solution of the differently substituted phenol **1a**–**1t** (1.1 mmol) and cesium carbonate (0.5 mmol) in DMF (100 mL) was stirred at room temperature for 30 min. Subsequently, the 2‐chloro‐5‐acetylpyridine (1 mmol) was added and the reaction mixture was heated at 90°C for 12 h. After cooling, the solvent was removed under vacuum, and the residue was purified by silica gel column chromatography using a gradient elution of petroleum ether/ethyl acetate (PE/EA, 100:1 to 20:1) to afford the compounds **2a–2t**.

#### 3.2.2 General Procedure for Synthesis of Compounds **3a–3t**

A solution of compounds **2a–2t** (1 mmol) in anhydrous ethanol (50 mL) was added hydroxylamine hydrochloride (1.1 mmol) at room temperature under argon. The reaction mixture was refluxed for 6 h. After completion of the reaction, the solution was poured into ice‐water and the solid was filtered, washed with cool ethanol, dried under vacuum to afford the compounds **3a–3t**.

#### 3.2.3 General Procedure for Synthesis of Compounds **4a–4t**

To a solution of compounds **3a–3t** (1 mmol) in ethanol (40 mL) and water (20 mL) was added zinc powder (2 mmol) and hydrochloric acid (6 mmol, 2 M) in four portions. After addition, the reaction mixture was heated at 50°C for 6 h. After completion, the solution was neutralized with sat. aq NaHCO solution, and extracted with ethyl acetate (50 mL × 2). The combined organic layers were dried with anhydrous Na_2_SO_4_, and concentrated under vacuum. The obtained residue was purified by silica gel column chromatography (PE/EtOAc, 1:1, v/v) to afford compounds **4a–4t**.

#### 3.2.4 General Procedure for Synthesis of Compounds **X1**–**X20**

The IQ3CA (1 mmol), 10 mL of thionyl chloride, and three drops of DMF (catalytic amount) were heated at 50°C for 3 h in a 50 mL round‐bottom flask. After completion, the solvent was removed under reduced pressure to afford the acyl chloride intermediate. To a solution of the intermediate (1 mmol) in dry ethyl acetate (5 mL) and dry pyridine (2 mL) was added compounds **4a–4t** (1 mmol) under argon. The reaction mixture was stirred overnight and quenched with iced water (10 mL). The mixture was diluted with EtOAc (20 mL) and washed with water (30 mL × 3), sat. NaHCO_3_ (30 mL × 3), brine (30 mL), and dried (anhydrous Na_2_SO_4_). The obtained residue was purified by silica gel column chromatography (PE/EtOAc, 2:1 v/v) to afford the target compounds **X1**–**X20**.

*N*‐(1‐(6‐(*p*‐Tolyloxy)pyridin‐3‐yl)ethyl)isoquinoline‐3‐carboxamide (**X1**). Yield: 85.55%. White solid. m.p. 160°C–161°C. ^1^H NMR (400 MHz, DMSO‐*d*_6_) δ 9.39 (s, 1H), 9.27 (d, *J* = 8.5 Hz, 1H), 8.54 (s, 1H), 8.25 (d, *J* = 8.1 Hz, 1H), 8.22–8.16 (m, 2H), 7.96 (dd, *J* = 8.5, 2.5 Hz, 1H), 7.89–7.84 (m, 1H), 7.80 (t, *J* = 7.7 Hz, 1H), 7.18 (d, *J* = 8.1 Hz, 2H), 6.96 (t, *J* = 8.7 Hz, 3H), 5.26 (q, *J* = 7.3 Hz, 1H), 2.29 (s, 3H), 1.58 (d, *J* = 7.1 Hz, 3H). ^13^C NMR (100 MHz, DMSO) δ 163.9, 162.8, 152.2, 152.0, 146.0, 144.2, 138.8, 135.9, 135.3, 133.9, 131.8, 130.5, 129.7, 129.6, 128.4, 128.3, 121.4, 120.4, 111.4, 46.3, 22.0, 20.8. HRMS (ESI‐TOF) *m*/*z*: [M + H]^+^ calcd for C_24_H_22_N_3_O_2_, 384.1712, found 384.1709.

*N*‐(1‐(6‐(3,5‐Dichlorophenoxy)pyridin‐3‐yl)ethyl)isoquinoline‐3‐carboxamide (**X2**). Yield: 68.52%. Brown solid. m.p. 158°C–159°C. ^1^H NMR (400 MHz, DMSO‐*d*_6_) δ 9.37 (s, 1H), 9.30 (d, *J* = 8.4 Hz, 1H), 8.54 (s, 1H), 8.28 (d, *J* = 2.6 Hz, 1H), 8.21 (d, *J* = 8.0 Hz, 1H), 8.14 (d, *J* = 8.1 Hz, 1H), 8.04 (dd, *J* = 8.6, 2.6 Hz, 1H), 7.84–7.74 (m, 2H), 7.37 (s, 1H), 7.23 (s, 2H), 7.09 (d, *J* = 8.5 Hz, 1H), 5.34–5.27 (m, 1H), 1.60 (d, *J* = 7.0 Hz, 3H). ^13^C NMR (100 MHz, DMSO) δ 163.5, 161.0, 155.2, 151.4, 145.5, 143.6, 138.8, 135.9, 135.3, 134.4, 131.2, 129.2, 129.0, 127.9, 127.7, 124.2, 120.3, 119.9, 111.6, 45.8, 21.5. HRMS (ESI‐TOF) *m*/*z*: [M + H]^+^ calcd for C_23_H_18_Cl_2_N_3_O_2_, 438.0776, found 438.0784.

*N‐*(1‐(6‐(4‐(methylthio)phenoxy)pyridin‐3‐yl)ethyl)isoquinoline‐3‐carboxamide (**X3**). Yield: 70.56%. Pale yellow solid. m.p. 135°C–136°C. ^1^H NMR (400 MHz, DMSO‐*d*_6_) δ 9.37 (s, 1H), 9.28 (d, *J* = 8.4 Hz, 1H), 8.55 (s, 1H), 8.26–8.19 (m, 2H), 8.14 (d, *J* = 8.1 Hz, 1H), 7.99 (dd, *J* = 8.5, 2.5 Hz, 1H), 7.82 (t, *J* = 7.5 Hz, 1H), 7.78–7.74 (m, 1H), 7.27 (d, *J* = 8.7 Hz, 2H), 7.05 (d, *J* = 8.7 Hz, 2H), 6.98 (d, *J* = 8.5 Hz, 1H), 5.29 (q, *J* = 7.4 Hz, 1H), 2.44 (s, 3H), 1.59 (d, *J* = 7.0 Hz, 3H). ^13^C NMR (100 MHz, DMSO) δ 163.4, 162.0, 151.6, 151.4, 145.5, 143.6, 138.4, 135.3, 135.0, 133.4, 131.3, 129.2, 129.0, 127.9, 127.7, 121.8, 119.9, 111.0, 45.8, 21.5, 15.5. HRMS (ESI‐TOF) *m*/*z*: [M + H]^+^ calcd for C_24_H_22_N_3_O_2_S, 416.1433, found 416.1434.

*N*‐(1‐(6‐(3‐(Trifluoromethyl)phenoxy)pyridin‐3‐yl)ethyl)isoquinoline‐3‐carboxamide (**X4**). Yield: 67.34%. Bright yellow solid. m.p. 92°C–93°C. ^1^H NMR (400 MHz, DMSO‐*d*_6_) δ 9.37 (s, 1H), 9.30 (d, *J* = 8.4 Hz, 1H), 8.55 (s, 1H), 8.27 (d, *J* = 2.6 Hz, 1H), 8.20 (d, *J* = 8.1 Hz, 1H), 8.13 (d, *J* = 8.2 Hz, 1H), 8.04 (dd, *J* = 8.5, 2.6 Hz, 1H), 7.81 (t, *J* = 7.4 Hz, 1H), 7.74 (t, *J* = 7.5 Hz, 1H), 7.59 (t, *J* = 8.0 Hz, 1H), 7.53–7.46 (m, 2H), 7.41 (d, *J* = 8.0 Hz, 1H), 7.09 (d, *J* = 8.5 Hz, 1H), 5.31 (q, *J* = 7.4 Hz, 1H), 1.60 (d, *J* = 7.1 Hz, 3H). ^13^C NMR (100 MHz, DMSO) δ 163.5, 161.5, 154.3, 151.4, 145.4, 143.7, 138.7, 135.6, 135.4, 131.2, 130.8, 130.6, 130.2, 129.2, 129.0, 127.9, 127.8, 127.7, 125.3, 125.1, 122.4, 121.0, 120.9, 120.0, 117.9, 117.9, 111.5, 45.9, 21.5. ^19^F NMR (376 MHz, DMSO) δ −61.2. HRMS (ESI‐TOF) *m*/*z*: [M + H]^+^ calcd for C_24_H_19_F_3_N_3_O_2_, 438.1429, found 438.1440.

*N*‐(1‐(6‐(3,5‐Difluorophenoxy)pyridin‐3‐yl)ethyl)isoquinoline‐3‐carboxamide (**X5**). Yield: 60.77%. White solid. m.p. 157°C–158°C. ^1^H NMR (400 MHz, DMSO‐*d*_6_) δ 9.39 (s, 1H), 9.30 (d, *J* = 8.5 Hz, 1H), 8.54 (s, 1H), 8.29 (d, *J* = 2.5 Hz, 1H), 8.24 (d, *J* = 8.1 Hz, 1H), 8.16 (d, *J* = 8.2 Hz, 1H), 8.04 (dd, *J* = 8.5, 2.6 Hz, 1H), 7.85 (t, *J* = 7.5 Hz, 1H), 7.79 (t, *J* = 7.6 Hz, 1H), 7.11–7.03 (m, 2H), 6.93 (dd, *J* = 8.3, 2.4 Hz, 2H), 5.30 (q, *J* = 7.4 Hz, 1H), 1.60 (d, *J* = 7.0 Hz, 3H). ^13^C NMR (100 MHz, DMSO) δ 164.0, 163.8, 163.5, 161.5, 161.4, 160.9, 156.1, 156.0, 155.8, 151.5, 145.6, 143.6, 138.8, 136.0, 135.4, 131.3, 129.2, 129.1, 127.9, 127.8, 120.0, 111.7, 105.2, 105.0, 104.9, 100.2, 100.0, 99.7, 45.8, 21.5. ^19^F NMR (376 MHz, DMSO) δ −109.1. HRMS (ESI‐TOF) *m*/*z*: [M + H]^+^ calcd for C_23_H_18_F_2_N_3_O_2_, 406.1367, found 406.1369.

*N*‐(1‐(6‐(4‐Chlorophenoxy)pyridin‐3‐yl)ethyl)isoquinoline‐3‐carboxamide (**X6**). Yield: 78.52%. White solid. m.p. 177°C–178°C. ^1^H NMR (400 MHz, CDCl_3_) δ 9.14 (s, 1H), 8.59 (s, 1H), 8.50 (d, *J* = 8.3 Hz, 1H), 8.27 (d, *J* = 2.6 Hz, 1H), 8.03 (d, *J* = 8.1 Hz, 1H), 7.97 (d, *J* = 8.1 Hz, 1H), 7.81–7.74 (m, 2H), 7.73–7.67 (m, 1H), 7.33 (d, *J* = 8.8 Hz, 2H), 7.06 (d, *J* = 8.8 Hz, 2H), 6.91 (d, *J* = 8.5 Hz, 1H), 5.38 (q, *J* = 7.2 Hz, 1H), 1.67 (d, *J* = 7.0 Hz, 3H). ^13^C NMR (100 MHz, CDCl_3_) δ 164.2, 162.8, 152.8, 151.2, 145.7, 143.4, 138.2, 136.1, 134.2, 131.3, 129.9, 129.9, 129.8, 129.1, 128.3, 127.8, 122.7, 120.6, 111.6, 46.4, 22.0. HRMS (ESI‐TOF) *m*/*z*: [M + H]^+^ calcd for C_23_H_19_ClN_3_O_2_, 404.1166, found 404.1164.

*N*‐(1‐(6‐(3‐Methoxyphenoxy)pyridin‐3‐yl)ethyl)isoquinoline‐3‐carboxamide (**X7**). Yield: 86.64%. White solid. m.p. 134°C–135°C. ^1^H NMR (400 MHz, DMSO‐*d*_6_) δ 9.39 (s, 1H), 9.30 (d, *J* = 8.4 Hz, 1H), 8.53 (s, 1H), 8.29–8.21 (m, 2H), 8.18 (d, *J* = 8.1 Hz, 1H), 7.98 (dd, *J* = 8.6, 2.5 Hz, 1H), 7.90–7.84 (m, 1H), 7.80 (t, *J* = 7.6 Hz, 1H), 7.28 (t, *J* = 8.2 Hz, 1H), 6.98 (d, *J* = 8.5 Hz, 1H), 6.77 (dd, *J* = 8.3, 2.5 Hz, 1H), 6.69–6.62 (m, 2H), 5.27 (q, *J* = 7.3 Hz, 1H), 3.72 (s, 3H), 1.58 (d, *J* = 7.1 Hz, 3H). ^13^C NMR (100 MHz, DMSO) δ 163.5, 161.9, 160.4, 155.2, 151.5, 145.6, 143.6, 138.4, 135.3, 135.1, 131.3, 130.1, 129.2, 129.1, 127.9, 127.8, 119.9, 112.9, 111.1, 110.1, 106.9, 55.3, 45.8, 21.5. HRMS (ESI‐TOF) *m*/*z*: [M + H]^+^ calcd for C_24_H_22_N_3_O_3_, 400.1661, found 400.1658.

*N*‐(1‐(6‐(4‐(Trifluoromethyl)phenoxy)pyridin‐3‐yl)ethyl)isoquinoline‐3‐carboxamide (**X8**). Yield: 64.66%. Brown solid. m.p. 122°C–123°C. ^1^H NMR (400 MHz, CDCl_3_) δ 9.14 (s, 1H), 8.59 (s, 1H), 8.53 (d, *J* = 8.1 Hz, 1H), 8.29 (d, *J* = 2.5 Hz, 1H), 8.03 (d, *J* = 8.1 Hz, 1H), 7.97 (d, *J* = 8.1 Hz, 1H), 7.83 (dd, *J* = 8.5, 2.6 Hz, 1H), 7.76 (t, *J* = 7.5 Hz, 1H), 7.70 (t, *J* = 7.5 Hz, 1H), 7.62 (d, *J* = 8.4 Hz, 2H), 7.22 (d, *J* = 8.4 Hz, 2H), 6.96 (d, *J* = 8.4 Hz, 1H), 5.40 (q, *J* = 7.2 Hz, 1H), 1.68 (d, *J* = 7.0 Hz, 3H). ^13^C NMR (100 MHz, CDCl_3_) δ 164.3, 162.2, 157.2, 151.3, 145.8, 143.4, 138.4, 136.1, 134.9, 131.3, 129.9, 129.1, 128.3, 127.8, 127.1, 127.1, 121.2, 120.7, 112.2, 46.5, 22.0. HRMS (ESI‐TOF) *m*/*z*: [M + H]^+^ calcd for C_24_H_19_F_3_N_3_O_2_, 438.1429, found 438.1429.

*N*‐(1‐(6‐(3‐Chlorophenoxy)pyridin‐3‐yl)ethyl)isoquinoline‐3‐carboxamiden (**X9**). Yield: 74.43%. White solid. m.p. 130°C–131°C. ^1^H NMR (400 MHz, DMSO‐*d*_6_) δ 9.37 (s, 1H), 9.31 (d, *J* = 8.4 Hz, 1H), 8.53 (s, 1H), 8.27–8.19 (m, 2H), 8.14 (d, *J* = 8.0 Hz, 1H), 8.01 (dd, *J* = 8.5, 2.5 Hz, 1H), 7.86–7.80 (m, 1H), 7.79–7.74 (m, 1H), 7.39 (t, *J* = 8.1 Hz, 1H), 7.26–7.17 (m, 2H), 7.05 (t, *J* = 8.0 Hz, 2H), 5.29 (q, *J* = 7.2 Hz, 1H), 1.59 (d, *J* = 7.1 Hz, 3H). ^13^C NMR (100 MHz, DMSO) δ 163.6, 161.5, 154.9, 151.5, 145.5, 143.7, 138.7, 135.6, 135.4, 133.5, 131.3, 131.0, 129.3, 129.1, 127.9, 127.8, 124.4, 121.2, 120.0, 119.8, 111.5, 45.9, 21.5. HRMS (ESI‐TOF) *m*/*z*: [M + H]^+^ calcd for C_23_H_19_ClN_3_O_2_, 404.1166, found 404.1163.

*N*‐(1‐(6‐Phenoxypyridin‐3‐yl)ethyl)isoquinoline‐3‐carboxamide (**X10**). Yield: 80.21%. White solid. m.p. 120°C–121°C^. 1^H NMR (400 MHz, DMSO‐*d*_6_) δ 9.40 (s, 1H), 9.32 (d, *J* = 8.5 Hz, 1H), 8.54 (s, 1H), 8.26 (d, *J* = 8.1 Hz, 1H), 8.23–8.15 (m, 2H), 7.99 (d, *J* = 8.5 Hz, 1H), 7.87 (t, *J* = 7.4 Hz, 1H), 7.81 (t, *J* = 7.4 Hz, 1H), 7.39 (t, *J* = 7.6 Hz, 2H), 7.19 (t, *J* = 7.4 Hz, 1H), 7.09 (d, *J* = 8.0 Hz, 2H), 7.00 (d, *J* = 8.5 Hz, 1H), 5.26 (q, *J* = 7.4 Hz, 1H), 1.58 (d, *J* = 7.0 Hz, 3H). ^13^C NMR (100 MHz, DMSO) δ 163.4, 162.0, 153.9, 151.5, 145.5, 143.7, 138.4, 135.3, 135.0, 131.3, 129.6, 129.2, 129.1, 127.9, 127.8, 124.3, 120.9, 119.9, 111.1, 45.8, 21.5. HRMS (ESI‐TOF) *m*/*z*: [M + H]^+^ calcd for C_23_H_20_N_3_O_2_, 370.1556, found 370.1556.

*N*‐(1‐(6‐(4‐(*tert*‐Butyl)phenoxy)pyridin‐3‐yl)ethyl)isoquinoline‐3‐carboxamide (**X11**). Yield: 74.13%. White solid. m.p. 140°C–141°C. ^1^H NMR (400 MHz, DMSO‐*d*_6_) δ 9.37 (s, 1H), 9.31 (d, *J* = 8.4 Hz, 1H), 8.57 (s, 1H), 8.30–8.17 (m, 2H), 8.14 (d, *J* = 8.1 Hz, 1H), 7.98 (dd, *J* = 8.5, 2.5 Hz, 1H), 7.81 (t, *J* = 7.5 Hz, 1H), 7.74 (t, *J* = 6.9 Hz, 1H), 7.33 (d, *J* = 8.8 Hz, 2H), 6.97 (t, *J* = 8.8 Hz, 3H), 5.31 (p, *J* = 7.3 Hz, 1H), 1.59 (d, *J* = 7.0 Hz, 3H), 1.21 (s, 9H). ^13^C NMR (100 MHz, DMSO) δ 163.4, 162.2, 151.6, 151.4, 146.5, 145.5, 143.7, 138.3, 135.3, 134.8, 131.2, 129.2, 129.0, 127.9, 127.7, 126.2, 120.5, 119.9, 111.0, 45.8, 34.0, 31.1, 21.5. HRMS (ESI‐TOF) *m*/*z*: [M + H]^+^ calcd for C_27_H_28_N_3_O_2_, 426.2182, found 426.2172.

*N*‐(1‐(6‐(2‐Chloro‐4‐(trifluoromethyl)phenoxy)pyridin‐3‐yl)ethyl)isoquinoline‐3‐carboxamide (**X12**). Yield: 56.87%. Brown solid. m.p. 142°C–143°C. ^1^H NMR (400 MHz, DMSO‐*d_6_*) δ 9.39 (d, *J* = 9.1 Hz, 2H), 8.55–8.49 (m, 2H), 8.25 (d, *J* = 8.2 Hz, 1H), 8.17 (d, *J* = 8.3 Hz, 1H), 7.97 (dd, *J* = 8.3, 2.6 Hz, 1H), 7.85 (d, *J* = 6.8 Hz, 1H), 7.80 (t, *J* = 6.9 Hz, 1H), 7.48 (d, *J* = 8.3 Hz, 1H), 5.33–5.26 (m, 1H), 1.59 (d, *J* = 7.1 Hz, 3H). ^13^C NMR (100 MHz, DMSO) δ 163.7, 151.6, 151.4, 148.6, 148.4, 148.2, 143.6, 139.5, 137.9, 135.4, 131.4, 129.3, 129.2, 128.0, 127.8, 124.0, 120.1, 120.0, 45.9, 21.4. ^19^F NMR (376 MHz, DMSO) δ −55.9, −55.9, −56.0. HRMS (ESI‐TOF) *m*/*z*: [M + H]^+^ calcd for C_24_H_18_ClF_3_N_3_O_2_, 472.1040, found 472.1037.

*N*‐(1‐(6‐(4‐Fluorophenoxy)pyridin‐3‐yl)ethyl)isoquinoline‐3‐carboxamide (**X13**). Yield: 70.55%. Gray solid. m.p. 175°C–176°C. ^1^H NMR (400 MHz, DMSO‐*d*_6_) δ 9.40 (s, 1H), 9.34 (d, *J* = 8.5 Hz, 1H), 8.54 (s, 1H), 8.27–8.22 (m, 2H), 8.17 (d, *J* = 8.2 Hz, 1H), 8.03 (d, *J* = 8.5 Hz, 1H), 7.88–7.82 (m, 1H), 7.82–7.76 (m, 1H), 7.41 (q, *J* = 8.6 Hz, 1H), 7.09–7.01 (m, 3H), 6.95 (d, *J* = 8.4 Hz, 1H), 5.29 (p, *J* = 7.3 Hz, 1H), 1.59 (d, *J* = 7.0 Hz, 3H). ^13^C NMR (100 MHz, DMSO) δ 163.7, 163.5, 161.4, 161.3, 155.2, 151.5, 145.5, 143.6, 138.6, 135.5, 135.3, 131.3, 130.8, 130.7, 129.2, 129.1, 127.9, 127.7, 119.9, 117.0, 111.4, 111.2, 111.0, 108.7, 108.5, 45.8, 21.5. ^19^F NMR (376 MHz, DMSO) δ −118.8. HRMS (ESI‐TOF) *m*/*z*: [M + H]^+^ calcd for C_23_H_19_FN_3_O_2_, 388.1461, found 388.1455.

*N*‐(1‐(6‐(4‐Methoxyphenoxy)pyridin‐3‐yl)ethyl)isoquinoline‐3‐carboxamide (**X14**). Yield: 74.13%. White solid. m.p. 160°C–161°C. ^1^H NMR (400 MHz, DMSO‐*d*_6_) δ 9.48–9.20 (m, 2H), 8.54 (s, 1H), 8.30–8.09 (m, 3H), 7.96 (s, 1H), 7.88–7.71 (m, 2H), 7.11–6.87 (m, 5H), 5.39–5.17 (m, 1H), 3.73 (s, 3H), 1.58 (d, *J* = 7.0 Hz, 3H). ^13^C NMR (100 MHz, DMSO) δ 163.4, 162.6, 156.0, 151.4, 145.4, 143.6, 138.1, 135.3, 134.5, 131.2, 129.2, 129.0, 127.8, 127.7, 122.2, 119.8, 114.6, 110.5, 55.3, 45.8, 21.4. HRMS (ESI‐TOF) *m*/*z*: [M + H]^+^ calcd for C_24_H_22_N_3_O_3_, 400.1661, found 400.1660.

*N*‐(1‐(6‐(3‐Fluorophenoxy)pyridin‐3‐yl)ethyl)isoquinoline‐3‐carboxamide (**X15**). Yield: 50.61%. Brown solid. m.p. 141°C–142°C. ^1^H NMR (400 MHz, DMSO‐*d*_6_) δ 9.39 (s, 1H), 9.29 (d, *J* = 8.4 Hz, 1H), 8.54 (s, 1H), 8.25 (d, *J* = 9.0 Hz, 2H), 8.17 (d, *J* = 8.1 Hz, 1H), 8.02 (dd, *J* = 8.5, 2.5 Hz, 1H), 7.89–7.83 (m, 1H), 7.80 (t, *J* = 7.5 Hz, 1H), 7.45–7.38 (m, 1H), 7.04 (t, *J* = 9.7 Hz, 3H), 6.95 (d, *J* = 9.2 Hz, 1H), 5.29 (p, *J* = 7.2 Hz, 1H), 1.59 (d, *J* = 7.0 Hz, 3H). ^13^C NMR (100 MHz, DMSO) δ 163.8, 163.5, 161.5, 161.3, 155.3, 155.2, 151.5, 145.5, 143.7, 138.6, 135.6, 135.4, 131.3, 130.8, 130.7, 129.2, 129.1, 127.9, 127.8, 120.0, 117.0, 117.0, 111.4, 111.3, 111.0, 108.8, 108.5, 45.8, 21.5. ^19^F NMR (376 MHz, DMSO) δ −111.5. HRMS (ESI‐TOF) *m*/*z*: [M + H]^+^ calcd for C_23_H_19_FN_3_O_2_, 388.1461, found 388.1461.

*N*‐(1‐(6‐(4‐Cyanophenoxy)pyridin‐3‐yl)ethyl)isoquinoline‐3‐carboxamide (**X16**). Yield: 70.72%. White solid. m.p. 155°C–156°C. ^1^H NMR (400 MHz, DMSO‐*d*_6_) δ 9.45–9.27 (m, 2H), 8.54 (s, 1H), 8.30 (d, *J* = 2.4 Hz, 1H), 8.22 (d, *J* = 8.1 Hz, 1H), 8.14 (d, *J* = 8.1 Hz, 1H), 8.06 (dd, *J* = 8.5, 2.5 Hz, 1H), 7.90–7.71 (m, 4H), 7.26 (d, *J* = 8.7 Hz, 2H), 7.12 (d, *J* = 8.5 Hz, 1H), 5.38–5.26 (m, 1H), 1.60 (d, *J* = 7.1 Hz, 3H). ^13^C NMR (100 MHz, DMSO) δ 163.5, 160.7, 158.0, 151.4, 145.7, 143.6, 138.8, 136.3, 135.3, 134.1, 131.2, 129.2, 129.0, 127.9, 127.7, 121.3, 119.9, 118.5, 112.1, 106.5, 45.8, 21.4. HRMS (ESI‐TOF) *m*/*z*: [M + H]^+^ calcd for C_24_H_19_N_4_O_2_, 395.1508, found 395.1511.

*N*‐(1‐(6‐(4‐Ethylphenoxy)pyridin‐3‐yl)ethyl)isoquinoline‐3‐carboxamide (**X17**). Yield: 80.86%. White solid. m.p. 130°C–131°C. ^1^H NMR (400 MHz, DMSO‐*d*_6_) δ 9.37 (s, 1H), 9.29 (d, *J* = 8.4 Hz, 1H), 8.55 (s, 1H), 8.29–8.16 (m, 2H), 8.13 (d, *J* = 8.1 Hz, 1H), 7.97 (dd, *J* = 8.5, 2.5 Hz, 1H), 7.81 (t, *J* = 6.8 Hz, 1H), 7.78–7.69 (m, 1H), 7.16 (d, *J* = 8.5 Hz, 2H), 7.04–6.90 (m, 3H), 5.29 (p, *J* = 7.2 Hz, 1H), 2.53 (q, *J* = 7.6 Hz, 2H), 1.58 (d, *J* = 7.0 Hz, 3H), 1.12 (t, *J* = 7.6 Hz, 3H). ^13^C NMR (100 MHz, DMSO) δ 163.4, 162.2, 151.9, 151.4, 145.4, 143.6, 139.7, 138.2, 135.3, 134.7, 131.2, 129.2, 129.0, 128.7, 127.8, 127.7, 120.8, 119.9, 110.9, 45.8, 27.4, 21.4, 15.5. HRMS (ESI‐TOF) *m*/*z*: [M + H]^+^ calcd for C_25_H_24_N_3_O_2_, 398.1869, found 398.1867.

*N*‐(1‐(6‐(4‐Propylphenoxy)pyridin‐3‐yl)ethyl)isoquinoline‐3‐carboxamide (**X18**). Yield: 76.52%. White solid. m.p. 136°C–137°C. ^1^H NMR (400 MHz, DMSO‐*d*_6_) δ 9.38 (s, 1H), 9.29 (d, *J* = 8.4 Hz, 1H), 8.56 (s, 1H), 8.27–8.19 (m, 2H), 8.15 (d, *J* = 8.2 Hz, 1H), 7.98 (dd, *J* = 8.5, 2.5 Hz, 1H), 7.82 (d, *J* = 7.9 Hz, 1H), 7.76 (t, *J* = 7.5 Hz, 1H), 7.14 (d, *J* = 8.4 Hz, 2H), 7.02–6.92 (m, 3H), 5.30 (p, *J* = 7.2 Hz, 1H), 2.48 (t, *J* = 7.5 Hz, 2H), 1.59 (d, *J* = 7.1 Hz, 3H), 1.52 (q, *J* = 7.5 Hz, 2H), 0.84 (t, *J* = 7.1 Hz, 3H). ^13^C NMR (100 MHz, DMSO) δ 163.4, 162.2, 152.0, 151.4, 145.5, 143.6, 138.2, 138.0, 135.3, 134.7, 131.2, 129.2, 129.0, 127.8, 127.7, 120.6, 119.8, 110.9, 45.8, 36.5, 24.0, 21.4, 13.5. HRMS (ESI‐TOF) *m*/*z*: [M + H]^+^ calcd for C_26_H_26_N_3_O_2_, 412.2025, found 412.2027.

*N‐*(1‐(6‐(4‐Isopropylphenoxy)pyridin‐3‐yl)ethyl)isoquinoline‐3‐carboxamide (**X19**). Yield: 69.20%. White solid; mp 122°C–123°C; **^1^H NMR** (400 MHz, DMSO‐*d*_6_) δ 9.38 (s, 1H), 9.29 (d, *J* = 8.7 Hz, 1H), 8.55 (s, 1H), 8.23 (d, *J* = 6.6 Hz, 2H), 8.16 (d, *J* = 8.1 Hz, 1H), 7.98 (dd, *J* = 8.6, 2.4 Hz, 1H), 7.87–7.81 (m, 1H), 7.81–7.74 (m, 1H), 7.26–7.17 (m, 2H), 7.03–6.91 (m, 3H), 5.34–5.22 (m, 1H), 1.58 (d, *J* = 6.5 Hz, 3H), 1.16 (dd, *J* = 7.0, 1.8 Hz, 6H); **^13^C NMR** (100 MHz, DMSO) δ 163.4, 162.2, 151.9, 151.4, 145.4, 144.3, 143.6, 138.2, 135.3, 134.7, 131.2, 129.2, 129.0, 127.9, 127.7, 127.2, 120.7, 119.8, 110.9, 45.8, 32.7, 23.8, 21.4; **HRMS** (ESI‐TOF) *m*/*z*: [M + H]^+^ calcd for C_26_H_26_N_3_O_2_, 412.2025, found 412.2023.

*N*‐(1‐(6‐(4‐Bromophenoxy)pyridin‐3‐yl)ethyl)isoquinoline‐3‐carboxamide (**X20**). Yield: 70.05%. Bright yellow solid; mp 145°C–146°C; **^1^H NMR** (400 MHz, DMSO‐*d*_6_) δ 9.38 (s, 1H), 9.30 (d, *J* = 8.5 Hz, 1H), 8.54 (s, 1H), 8.29–8.19 (m, 2H), 8.16 (d, *J* = 8.2 Hz, 1H), 8.06–7.96 (m, 1H), 7.89–7.74 (m, 2H), 7.54 (d, *J* = 8.8 Hz, 2H), 7.15–6.97 (m, 3H), 5.28 (p, *J* = 7.2 Hz, 1H), 1.58 (d, *J* = 7.1 Hz, 3H); **^13^C NMR** (100 MHz, DMSO) δ 163.4, 161.5, 153.3, 151.4, 145.4, 143.6, 138.5, 135.3, 132.3, 131.3, 129.2, 129.0, 127.9, 127.7, 123.3, 123.2, 119.9, 116.2, 111.3, 45.8, 21.4; **HRMS** (ESI‐TOF) *m*/*z*: [M + H]^+^ calcd for C_23_H_19_BrN_3_O_2_, 448.0661, found 448.0669.

### 3.3 Antifungal Activity Assay

#### 3.3.1 *In Vitro* Antifungal Assay

The *in vitro* antifungal activities of the target compounds were tested using the mycelial growth inhibition method [28, 29, 30]. All the tested compounds were dissolved in dimethyl sulfoxide (DMSO) and subsequently diluted with sterilized PDA medium to obtain concentrations of 50 and 100 µg/mL. The media‐containing compounds were then poured into Petri dishes and incubated at 25°C in the darkness for initial antifungal screening. For the EC_50_ calculation of the promising compounds, the target compounds were diluted to obtain a range of concentrations (100, 50, 25, 12.5, 6.25, and 3.125 mg/L). Boscalid was utilized as the positive control. Each sample was tested in triplicate. The results was calculated using the formula: mycelial growth inhibition (%) = (*C*—*T*)/(*C*—*d*) × 100%, where *d* represents the diameter of the fungal inoculum (4 mm), and *C* and *T* are the average colony diameter of the control and treatment groups, respectively.

#### 3.3.2 *In Vivo* Antifungal Assay

The protective and curative effects of compound **X6** against *B*. cinerea were assessed on tomato fruits by wounded assay [28]. The test compounds were initially dissolved in DMSO and subsequently diluted with 0.3% Tween‐80 distilled water to get concentrations of 50 and 100 µg/mL. For protective effect, healthy tomato fruits were soaked in compound **X6** solution for 5–10 s and dried. After drying at room temperature for 30 min, each wound was inoculated with *B. cinerea* to test preventative efficacy. For curative effect, healthy tomato fruits were cultivated with *B. cinerea* for 24 h and subsequently sprayed with compound **X6** solution to test curative efficacy. Boscalid was used as the positive control. The diameters of symptoms were measured after cultivation for 5 days at 25°C. The results was calculated using the formula: efficacy (%) = [(*C*—*T*)/(*C*—*d*)] × 100%, where *d* represents the diameter of the fungal inoculum (4 mm), and *C* and *T* are the average colony diameter of the control and treatment groups, respectively.

### 3.4 Mycelial Morphological Observations

Fresh mycelia were collected from 3‐ to 4‐day‐old cultures on Petri dishes and inoculated into liquid medium. Compound **X6** was dissolved in DMSO and added to the medium to achieve final concentrations of 2 × EC_50_ and 4 × EC_50_. An equivalent concentration of DMSO was used as the negative control, and boscalid served as the positive control. After 48 h of treatment, the samples were rinsed thoroughly with 0.1 M PBS at 4°C to remove the culture medium. Subsequently, the treated samples were immersed in 2.5% glutaraldehyde solution and fixed in a refrigerator at 4°C for 4–8 h. Following fixation, the samples were washed with gradient ethanol and isoamyl acetate, dried at the critical point, gold‐sprayed, and observed under scanning electron microscope.

### 3.5 Molecular Docking

To elucidate the fungicidal mechanism of compounds **X6** and **X16** against *B. cinerea*, molecular docking was performed for boscalid, **X6**, and **X16** against SDH. The protein model was derived from the first structurally characterized fungal SDH, sourced from *S. cerevisiae*, with its crystal structure (PDB ID: 9KPT) obtained from the RCSB Protein Data Bank (https://www.rcsb.org/). Docking software was conducted using AutoDock Tools 1.5.7, and the resulting ligand–protein interaction patterns were visualized and analyzed with PyMOL 1.0.0.

### 3.6 Molecular Dynamic Simulation

To systematically investigate the conformational dynamics and structural stability of the **X6–**protein and boscalid–protein complexes, 100 ns all‐atom molecular dynamics simulations were performed with Gromacs 2023.3. The optimal docking poses of each complex were used as the initial simulation structures. The AMBER14SB force field was applied for protein parametrization. Hydrogen atoms were added to the protein via the pdb2gmx module. Each complex was solvated in a cubic TIP3P water box with a 10 Å buffer distance, and Na^+^/Cl^−^ counterions were randomly added to neutralize the net charge of each system [31, 32]. Prior to the production run, all systems were sequentially equilibrated through energy minimization, NVT isothermal equilibration, and NPT isobaric equilibration. Periodic boundary conditions were applied throughout the simulation. Structural snapshots were collected every 10 ps, and the trajectories were analyzed for RMSD, radius of gyration (Rg), and MM‐GBSA binding free energies.

### 3.7 SDH Enzyme Activity Assay

Succinate dehydrogenase (SDH) activity was determined using a commercial kit (Solarbio, BC0955). The *B. cinerea* was cultured in sterilized potato dextrose broth (PDB) for 72 h and then treated with 1/2 × EC_50_, EC_50_, 2 × EC_50_, and 4 × EC_50_ concentrations of compound **X6**. The boscalid was used as positive controls. After 24 h, the mycelium was filtered and the SDH enzyme activity was determined using the SDH assay kit according to the instructions. All experiments were performed in three independent biological replicates.

### 3.8 Statistical Analysis

Experiments were conducted in triplicate, with data presented as mean ± standard deviation. Statistical analysis was performed using the SPSS software (version 20.0, IBM Corp., Armonk, NY, USA). Statistical significance was determined as *p* < 0.05 through the application of Duncan's multiple range tests, and the outcomes were depicted in lowercase alphabets in both figures and tables.

## 4 Conclusion

In summary, a series of isoquinoline‐3‐amide derivatives containing phenoxypyridine moieties were designed and synthesized, and their antifungal activity against five phytopathogens was assessed. The phenoxypyridine combined with isoquinoline‐3‐carboxylic acid can improve the sensitivity of fungi to the antifungal agents. Among them, compounds **X6**, **X9**, **X14**, and **X16** exhibited potent *in vitro* inhibitory effects against *B. cinerea*, *C. gloeosporioides*, and *R. solani*. Notably, compound **X6** demonstrated significant antifungal activity against *B. cinerea*, with an EC_50_ value of 7.55 µg/mL, which was comparable to that of the positive control boscalid (EC_50_ = 5.39 µg/mL). Furthermore, *in vivo* antifungal assays indicated that compound **X6** possessed good protective effects against *B. cinerea* at 100 µg/mL, achieving a control efficacy of 77.86%. In curative activity tests, **X6** exhibited a treatment efficacy of 46.45%, better than that of boscalid (33.69%). Molecular docking studies, molecular dynamic simulation, and SDH enzyme assay further confirmed that these compounds exhibited strong binding affinity and potent inhibitory effect against SDH. Collectively, these experimental and computational findings suggest that isoquinoline derivatives incorporating phenoxypyridine fragments possess potent fungicidal properties for crop protection, with compound **X6** emerging as a promising candidate for novel SDH inhibitors.

## Author Contributions

**Jiayao Zhang** contributed to investigation, formal analysis, and the preparation of the original draft. **Shuaipeng Sun** was involved in methodology development and validation. **Yajing Guo** contributed to the methodology and investigation. **Zhiyuan Xu** was responsible for data curation and formal analysis. **Jing Zhang** provided experimental facilities and supervision for anti‐fungal activity. **Haixin Ding** contributed to conceptualization, methodology design, reviewing and editing the manuscript. **Gang Feng** was involved in methodology, supervision, and contributed to reviewing and editing the manuscript. **Qiang Xiao** supervised the project and other necessary editing.

## Conflicts of Interest

The authors declare no conflicts of interest.

## Supporting information




**Supporting File 1**: cbdv71635‐sup‐0001‐SuppMat.pdf

## Data Availability

Data is provided within the manuscript or Supporting Information files.
