# Supplementary material for: Synthesis and Antifungal Evaluation of Isoquinoline‐3‐amide Derivatives Containing a Phenoxypyridine Moiety for Plant Protection
Source: Chem Biodivers. 2026 Sep 10;23(9):e71635. doi: 10.1002/cbdv.71635 (PMC13559863; doi:10.1002/cbdv.71635)
Supplement: Supplementary file 1 [file CBDV-23-e71635-s001.pdf]

Supporting Information

Content

1. Antifungal activity of target compounds against pathogenic fungi......................... S2

2. MM- GBSA binding free energy decomposition analysis S3

3. Characterization of target compounds..S4

4. Crystal data of the target compound **X9**... S10

5. NMR and HRMS spectrum of the target compounds..S12

1. <u>Antifungal activity of target compounds against pathogenic fungi</u>

Table S1. In *vitro* antifungal activity of target compounds against five plant pathogenic fungi at 50 μg/mL.

Compound	*Inhibition rate (%)* †				
	B. cinerea	*P. capsici*	*C.gloeosporioides*	*R. solani*	*F. graminearum*
X1	33.42 ± 2.20	10.24 ± 1.53	29. 8 ± 3.28	31.7 ± 1.41	13.72 ± 2.43
X2	38.2 ± 0.70	12.25 ± 1.95	43.74 ± 1.52	10.15 ± 2.71	33.06 ± 0.44
X3	57.96 ± 1.40	13.66 ± 2.94	43.49 ± 0.78	56.41 ± 3.58	29.78 ± 0.76
X4	33.44 ± 0.40	30.92 ± 3.94	37.49 ± 0.78	51.69 ± 1.17	30.13 ± 0.53
X5	36.7 ± 0.40	21.1 ± 1.47	43.75 ± 1.56	53.58 ± 0.49	14.97 ± 1.60
X6	73.91 ± 0.81	22.85 ± 2.97	67.49 ± 1.17	62.61 ± 0.59	48.54 ± 0.76
X7	40.06 ±1.07	16.07 ± 1.95	32.11 ± 3.62	53.92 ± 1.95	32.2 ± 0.44
X8	36.27 ± 0.81	24.09 ± 3.32	40.68 ± 1.17	59.79 ± 3.10	27.13 ± 0.76
X9	36.7 ± 0.40	8.18 ± 3.90	52.8 ± 1.56	47.43 ± 0.40	0.66 ± 1.15
X10	11.82 ± 1.76	36.43 ± 3.37	37.48 ± 2.34	61.67 ± 0.78	15.02 ± 3.05
X11	8.96 ± 1.78	38.5 ± 3.63	16.69 ± 1.40	52.13 ± 0.78	30.58 ± 3.43
X12	34.85 ± 1.56	3.75 ± 1.12	18.49 ± 2.72	12.37 ± 3.10	38.96 ± 0.61
X13	27.33 ± 0.81	15.35 ± 1.12	30.82 ± 2.72	31.59 ± 2.38	9.54 ± 2.59
X14	40.09 ± 1.07	24.97 ± 4.25	57.92 ± 1.17	23.45 ± 1.03	20.48 ± 2.07
X15	42.33 ± 1.85	19.4 ± 1.27	26.68 ± 2.34	55.96 ± 2.35	51.12 ± 1.01
X16	60.19 ± 0.81	14.34 ± 1.53	68.23 ± 0.59	51.2 ± 3.34	18.48 ± 1.10
X17	40.07 ± 3.45	3.4 ± 1.12	44.87 ± 0.78	43.06 ± 1.35	31.42 ± 2.52
X18	33.32 ± 2.25	1.02 ± 3.11	40.76 ± 1.17	23.92 ± 4.08	22.55 ± 3.73
X19	38.19 ± 0.40	19.4 ± 1.53	32.91 ± 3.11	43.55 ± 3.23	26.19 ± 1.58
X20	34.79 ± 0.70	19.45 ± 0.74	31.17 ± 2.34	26.76 ± 2.72	17.11 ± 1.24
Boscalid	75.66 ± 0.81	33.89 ± 1.82	49.18 ± 1.17	94.28 ± 0.78	42.25 ± 3.50

† Values are the mean ± standard deviation (SD) of three replicates.

2. MM- GBSA binding free energy decomposition analysis

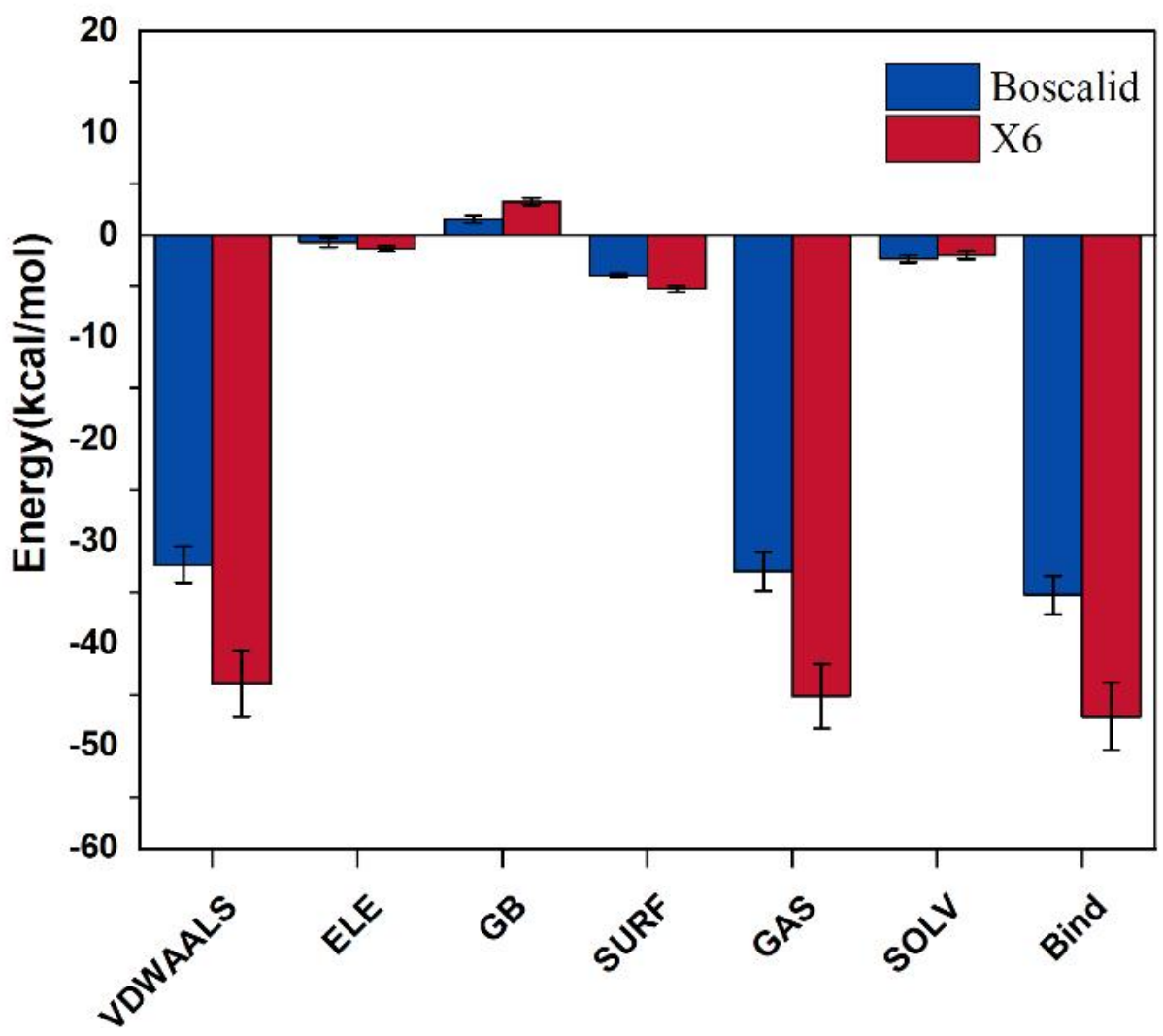

Energy	Energy values of boscalid (kcal/mol)	Energy values of **X6** (kcal/mol)
$\triangle E_{vdw}$	-32.21±1.77	-43.83±3.2
$\triangle E_{ele}$	-0.66±0.43	-1.28±0.28
$\triangle E_{GB}$	1.57±0.38	3.33±0.34
$\triangle E_{surf}$	-3.88±0.2	-5.27±0.32
$\triangle E_{Gas}$	-32.87±1.93	-45.11±3.18
$\triangle E_{solv}$	-2.31±0.36	-1.94±0.39
$\triangle E_{Bind}$	-35.19±1.88	-47.05±3.32

Figure S1. MM- GBSA binding free energy decomposition analysis of boscalid–protein and X6–protein complexes. ($\triangle E_{vdw}$, van der Waals energy; $\triangle E_{ele}$, electrostatic energy; $\triangle E_{Gas}$, gas-phase free energy ($\triangle E_{Gas} = \triangle E_{vdw} + \triangle E_{ele}$); $\triangle E_{surf}$, nonpolar solvation energy; $\triangle E_{GB}$, polar solvation energy; $\triangle E_{solv}$, total solvation energy ($\triangle E_{solv} = \triangle E_{GB} + \triangle E_{surf}$); $\triangle E_{Bind}$, total binding free energy ($\triangle E_{Bind} = \triangle E_{Gas} + \triangle E_{solv}$).)

3. Characterization of intermediate and target compounds

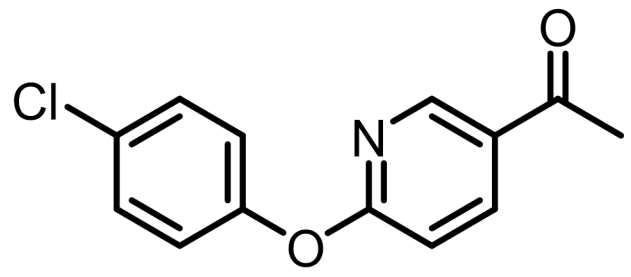

Data for intermediate compound **X6-2f :** Yield: 90.5 %; ^{1}H NMR (400 MHz, $CDCl_3$) δ 8.75 (d, *J* = 2.4 Hz, 1H), 8.28 (dd, *J* = 8.7, 2.5 Hz, 1H), 7.36 (t, *J* = 8.1 Hz, 1H), 7.23 (d, *J* = 8.2 Hz, 1H), 7.18 (t, *J* = 2.1 Hz, 1H), 7.06 (dd, *J* = 8.2, 2.3 Hz, 1H), 7.00 (d, *J* = 8.6 Hz, 1H), 2.58 (s, 3H); ^{13}C NMR (100 MHz, $CDCl_3$) *δ* 195.5, 164.9, 154.0, 150.9, 141.6, 134.3, 131.0, 125.4, 122.3, 120.0, 111.1, 104.0, 25.3.

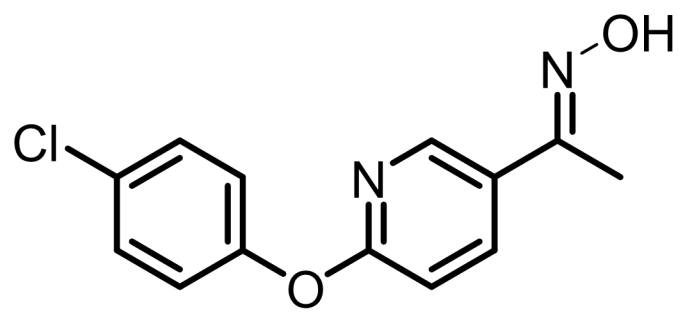

Data for intermediate compound **X6-3f :**Yield: 84.6%; ^{1}H NMR (400 MHz, $CDCl_3$) δ 8.42 (d, *J* = 2.6 Hz, 1H), 8.02 (dd, *J* = 8.7, 2.6 Hz, 1H), 7.81 (s, 1H), 7.33 (t, *J* = 8.1 Hz, 1H), 7.23 – 7.16 (m, 2H), 7.05 (d, *J* = 8.2 Hz, 1H), 6.94 (d, *J* = 8.6 Hz, 1H), 2.26 (s, 3H); ^{13}C NMR (100 MHz, $CDCl_3$) δ 165.1, 154.5, 143.9, 136.3, 134.8, 130.0, 127.8, 124.8, 121.9, 121.5, 119.6, 113.8, 111.6, 12.4.

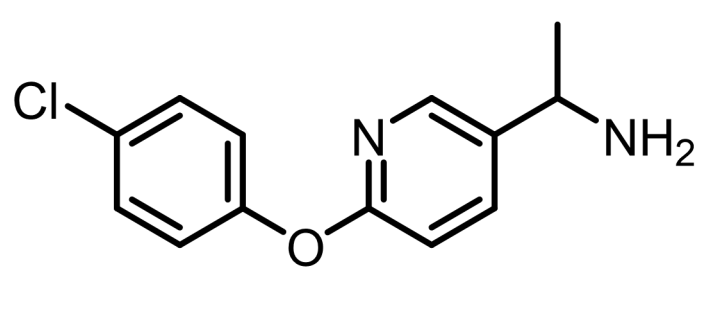

Data for intermediate compound **X6-4f:** Yield: 76.4%; ^{1}H NMR (400 MHz, DMSO-d_6) δ 8.16 (s, 1H), 7.96 (dd, *J* = 8.5, 2.5 Hz, 1H), 7.42 (t, *J* = 8.1 Hz, 1H), 7.25 (d, *J* = 7.8 Hz, 1H), 7.19 (s, 1H), 7.07 (d, *J* = 8.5 Hz, 2H), 4.19 (d, *J* = 6.8 Hz, 1H), 1.41 (d, *J* = 6.7 Hz, 3H); ^{13}C NMR (100 MHz, DMSO) δ 161.8, 155.0, 145.8, 138.8, 135.5, 133.6, 131.2, 124.6, 121.2, 119.9, 111.6, 48.1, 23.1.

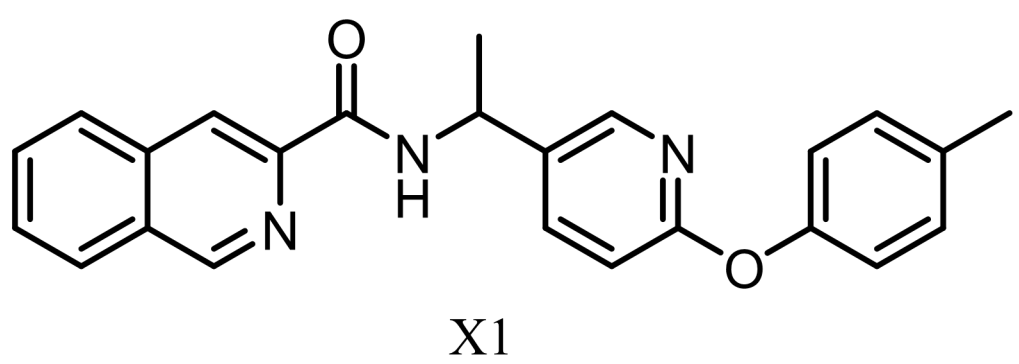

X1

***N*-(1-(6-(p-tolyloxy)pyridin-3-yl)ethyl)isoquinoline-3-carboxamide (X1)**

Yield: 85.55%; White solid; mp 160-161°C ; **^{1}H NMR** (400 MHz, DMSO-*d*$_6$) δ 9.39 (s, 1H), 9.27 (d, *J* = 8.5 Hz, 1H), 8.54 (s, 1H), 8.25 (d, *J* = 8.1 Hz, 1H), 8.22 – 8.16 (m, 2H), 7.96 (dd, *J* = 8.5, 2.5 Hz, 1H), 7.89 – 7.84 (m, 1H), 7.80 (t, *J* = 7.7 Hz, 1H), 7.18 (d, *J* = 8.1 Hz, 2H), 6.96 (t, *J* = 8.7 Hz, 3H), 5.26 (q, *J* = 7.3 Hz, 1H), 2.29 (s, 3H), 1.58 (d, *J* = 7.1 Hz, 3H); **^{13}C NMR** (100 MHz, DMSO) δ 163.9, 162.8, 152.2, 152.0, 146.0, 144.2, 138.8, 135.9, 135.3, 133.9, 131.8, 130.5, 129.7, 129.6, 128.4, 128.3, 121.4, 120.4, 111.4, 46.3, 22.0, 20.8; **HRMS** (ESI-TOF) m/z: $[M+H]^+$ calcd for $C_{24}H_{22}N_3O_2$, 384.1712, found 384.1709.

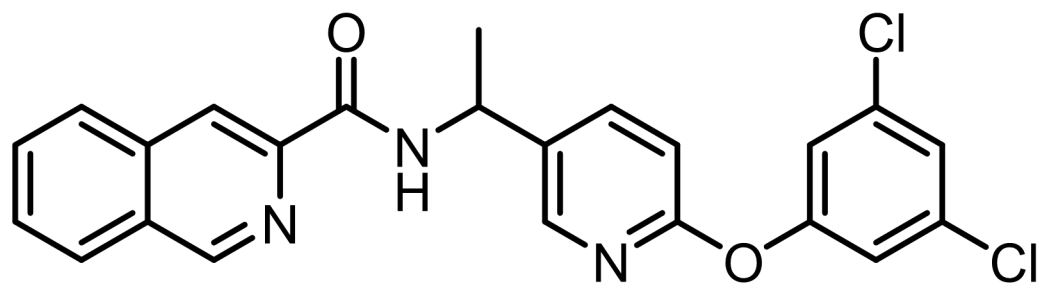

X2

***N*-(1-(6-(3,5-dichlorophenoxy)pyridin-3-yl)ethyl)isoquinoline-3-carboxamide (X2)**

Yield: 68.52%; Brown solid; mp 158-159°C ; **^{1}H NMR** (400 MHz, DMSO-*d*$_6$) δ 9.37 (s, 1H), 9.30 (d, J = 8.4 Hz, 1H), 8.54 (s, 1H), 8.28 (d, J = 2.6 Hz, 1H), 8.21 (d, J = 8.0 Hz, 1H), 8.14 (d, J = 8.1 Hz, 1H), 8.04 (dd, J = 8.6, 2.6 Hz, 1H), 7.84 – 7.74 (m, 2H), 7.37 (s, 1H), 7.23 (s, 2H), 7.09 (d, J = 8.5 Hz, 1H), 5.34 – 5.27 (m, 1H), 1.60 (d, J = 7.0 Hz, 3H); **^{13}C NMR** (100 MHz, DMSO) δ 163.5, 161.0, 155.2, 151.4, 145.5, 143.6, 138.8, 135.9, 135.3, 134.4, 131.2, 129.2, 129.0, 127.9, 127.7, 124.2, 120.3, 119.9, 111.6, 45.8, 21.5; **HRMS** (ESI-TOF) m/z: $[M+H]^+$ calcd for $C_{23}H_{18}Cl_2N_3O_2$, 438.0776, found 438.0784.

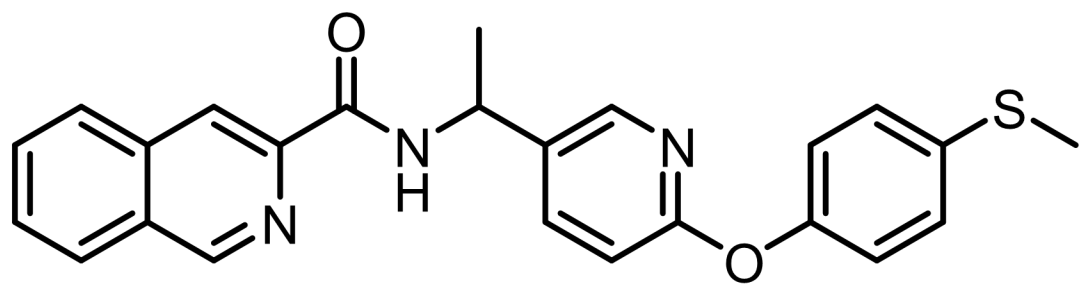

X3

***N*-(1-(6-(4-(methylthio)phenoxy)pyridin-3-yl)ethyl)isoquinoline-3-carboxamide (X3)**

Yield: 70.56%; Pale yellow solid; mp 135-136°C; **^{1}H NMR** (400 MHz, DMSO-*d*$_6$) δ 9.37 (s, 1H), 9.28 (d, *J* = 8.4 Hz, 1H), 8.55 (s, 1H), 8.26 – 8.19 (m, 2H), 8.14 (d, *J* = 8.1 Hz, 1H), 7.99 (dd, *J* = 8.5, 2.5 Hz, 1H), 7.82 (t, *J* = 7.5 Hz, 1H), 7.78 – 7.74 (m, 1H), 7.27 (d, *J* = 8.7 Hz, 2H), 7.05 (d, *J* = 8.7 Hz, 2H), 6.98 (d, *J* = 8.5 Hz, 1H), 5.29 (q, *J* = 7.4 Hz, 1H), 2.44 (s, 3H), 1.59 (d, *J* = 7.0 Hz, 3H); **^{13}C NMR** (100 MHz, DMSO) δ 163.4, 162.0, 151.6, 151.4, 145.5, 143.6, 138.4, 135.3, 135.0, 133.4, 131.3,

129.2, 129.0, 127.9, 127.7, 121.8, 119.9, 111.0, 45.8, 21.5, 15.5; **HRMS** (ESI-TOF) m/z: $[M+H]^+$ calcd for $C_{24}H_{22}N_3O_2S$, 416.1433, found 416.1434.

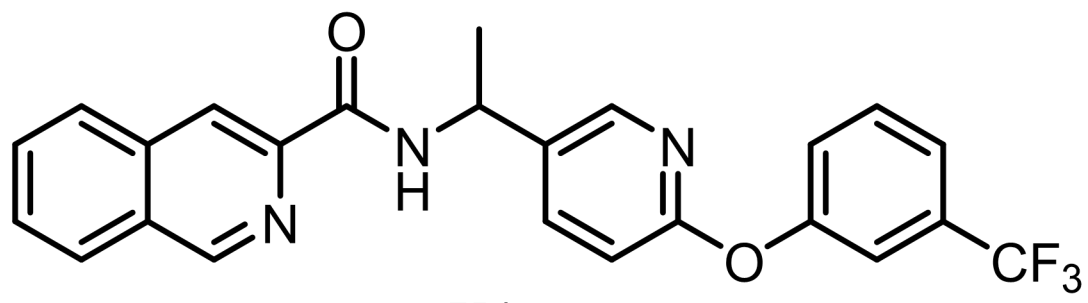

X4

***N*-(1-(6-(3-(trifluoromethyl)phenoxy)pyridin-3-yl)ethyl)isoquinoline-3-carboxamide(X4)**

Yield: 67.34%; Bright yellow solid; mp 92-93°C ; **^{1}H NMR** (400 MHz, DMSO-d_6) δ 9.37 (s, 1H), 9.30 (d, *J* = 8.4 Hz, 1H), 8.55 (s, 1H), 8.27 (d, *J* = 2.6 Hz, 1H), 8.20 (d, *J* = 8.1 Hz, 1H), 8.13 (d, *J* = 8.2 Hz, 1H), 8.04 (dd, *J* = 8.5, 2.6 Hz, 1H), 7.81 (t, *J* = 7.4 Hz, 1H), 7.74 (t, *J* = 7.5 Hz, 1H), 7.59 (t, *J* = 8.0 Hz, 1H), 7.53 – 7.46 (m, 2H), 7.41 (d, *J* = 8.0 Hz, 1H), 7.09 (d, *J* = 8.5 Hz, 1H), 5.31 (q, *J* = 7.4 Hz, 1H), 1.60 (d, *J* = 7.1 Hz, 3H); **^{13}C NMR** (100 MHz, DMSO) δ 163.5, 161.5, 154.3, 151.4, 145.4, 143.7, 138.7, 135.6, 135.4, 131.2, 130.8, 130.6, 130.2, 129.2, 129.0, 127.9, 127.8, 127.7, 125.3, 125.1, 122.4, 121.0, 120.9, 120.0, 117.9, 117.9, 111.5, 45.9, 21.5; **^{19}F NMR** (376 MHz, DMSO) δ -61.2; **HRMS** (ESI-TOF) m/z: $[M+H]^+$ calcd for $C_{24}H_{19}F_3N_3O_2$, 438.1429, found 438.1440.

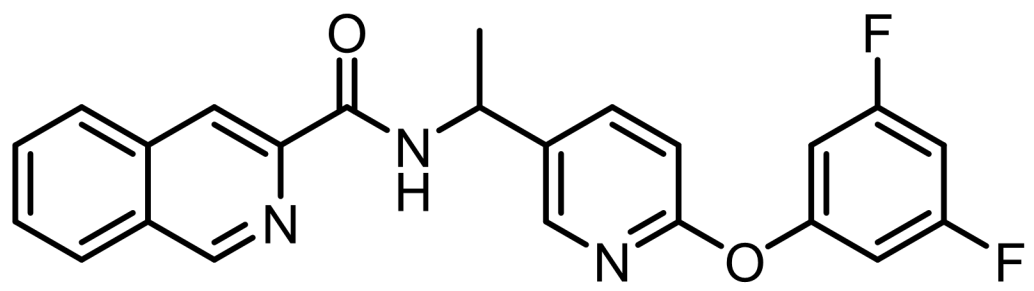

X5

***N*-(1-(6-(3,5-difluorophenoxy)pyridin-3-yl)ethyl)isoquinoline-3-carboxamide (X5)**

Yield: 60.77%; White solid; mp 157-158°C ; **^{1}H NMR** (400 MHz, DMSO-d_6) δ 9.39 (s, 1H), 9.30 (d, *J* = 8.5 Hz, 1H), 8.54 (s, 1H), 8.29 (d, *J* = 2.5 Hz, 1H), 8.24 (d, *J* = 8.1 Hz, 1H), 8.16 (d, *J* = 8.2 Hz, 1H), 8.04 (dd, *J* = 8.5, 2.6 Hz, 1H), 7.85 (t, *J* = 7.5 Hz, 1H), 7.79 (t, *J* = 7.6 Hz, 1H), 7.11 – 7.03 (m, 2H), 6.93 (dd, *J* = 8.3, 2.4 Hz, 2H), 5.30 (q, *J* = 7.4 Hz, 1H), 1.60 (d, *J* = 7.0 Hz, 3H); **^{13}C NMR** (100 MHz, DMSO) δ 164.0, 163.8, 163.5, 161.5, 161.4, 160.9, 156.1, 156.0, 155.8, 151.5, 145.6, 143.6, 138.8, 136.0, 135.4, 131.3, 129.2, 129.1, 127.9, 127.8, 120.0, 111.7, 105.2, 105.0, 104.9, 100.2, 100.0, 99.7, 45.8, 21.5; **^{19}F NMR** (376 MHz, DMSO) δ -109.1; **HRMS** (ESI-TOF) m/z: $[M+H]^+$ calcd for $C_{23}H_{18}F_2N_3O_2$, 406.1367, found 406.1369.

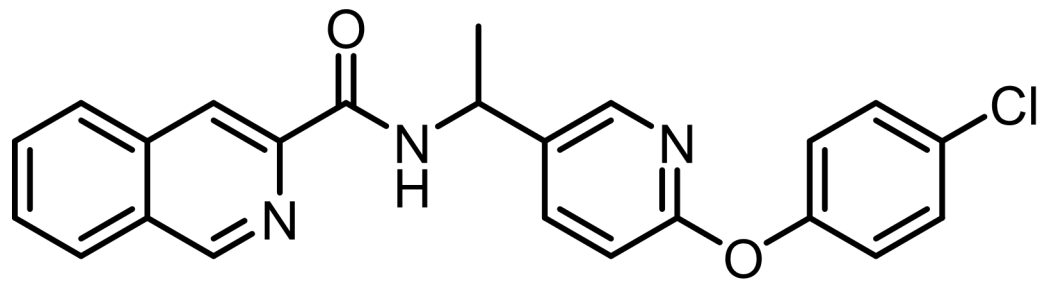

X6

N-(1-(6-(4-chlorophenoxy)pyridin-3-yl)ethyl)isoquinoline-3-carboxamide (X6)

Yield: 78.52%; White solid; mp 177-178 °C ; **^{1}H NMR** (400 MHz, $CDCl_3$) δ 9.14 (s, 1H), 8.59 (s, 1H), 8.50 (d, *J* = 8.3 Hz, 1H), 8.27 (d, *J* = 2.6 Hz, 1H), 8.03 (d, *J* = 8.1 Hz, 1H), 7.97 (d, *J* = 8.1 Hz, 1H), 7.81 – 7.74 (m, 2H), 7.73 – 7.67 (m, 1H), 7.33 (d, *J* = 8.8 Hz, 2H), 7.06 (d, *J* = 8.8 Hz, 2H), 6.91 (d, *J* = 8.5 Hz, 1H), 5.38 (q, *J* = 7.2 Hz, 1H), 1.67 (d, *J* = 7.0 Hz, 3H); **^{13}C NMR** (100 MHz, $CDCl_3$) δ 164.2, 162.8, 152.8, 151.2, 145.7, 143.4, 138.2, 136.1, 134.2, 131.3, 129.9, 129.9, 129.8, 129.1, 128.3, 127.8, 122.7, 120.6, 111.6, 46.4, 22.0; **HRMS** (ESI-TOF) m/z: $[M+H]^+$ calcd for $C_{23}H_{19}ClN_3O_2$, 404.1166, found 404.1164.

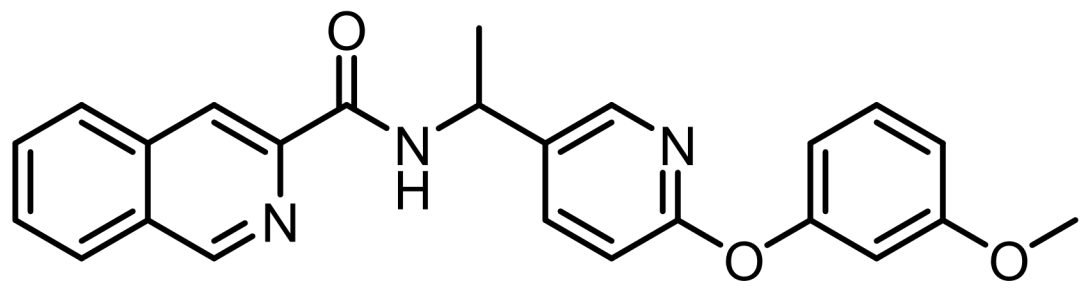

X7

N-(1-(6-(3-methoxyphenoxy)pyridin-3-yl)ethyl)isoquinoline-3-carboxamide (X7)

Yield: 86.64%; White solid; mp 134-135°C ; **^{1}H NMR** (400 MHz, DMSO-d_6) δ 9.39 (s, 1H), 9.30 (d, *J* = 8.4 Hz, 1H), 8.53 (s, 1H), 8.29 – 8.21 (m, 2H), 8.18 (d, *J* = 8.1 Hz, 1H), 7.98 (dd, *J* = 8.6, 2.5 Hz, 1H), 7.90 – 7.84 (m, 1H), 7.80 (t, *J* = 7.6 Hz, 1H), 7.28 (t, *J* = 8.2 Hz, 1H), 6.98 (d, *J* = 8.5 Hz, 1H), 6.77 (dd, *J* = 8.3, 2.5 Hz, 1H), 6.69 – 6.62 (m, 2H), 5.27 (q, *J* = 7.3 Hz, 1H), 3.72 (s, 3H), 1.58 (d, *J* = 7.1 Hz, 3H); **^{13}C NMR** (100 MHz, DMSO) δ 163.5, 161.9, 160.4, 155.2, 151.5, 145.6, 143.6, 138.4, 135.3, 135.1, 131.3, 130.1, 129.2, 129.1, 127.9, 127.8, 119.9, 112.9, 111.1, 110.1, 106.9, 55.3, 45.8, 21.5; **HRMS** (ESI-TOF) m/z: $[M+H]^+$ calcd for $C_{24}H_{22}N_3O_3$, 400.1661, found 400.1658.

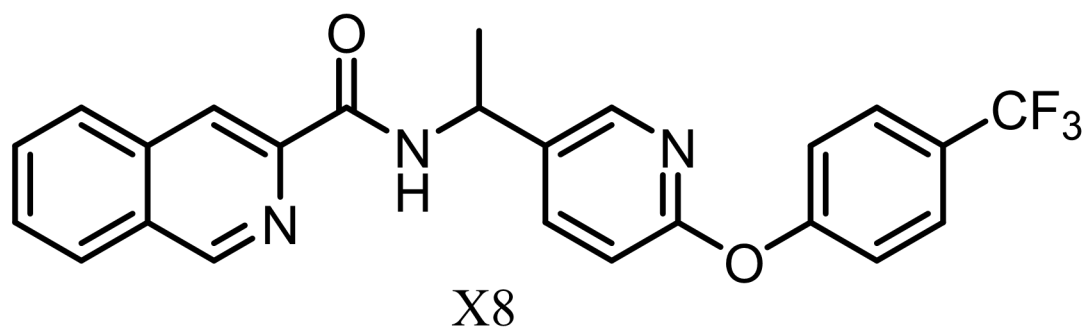

X8

N-(1-(6-(4-(trifluoromethyl)phenoxy)pyridin-3-yl)ethyl)isoquinoline-3-carboxamide (X8)

Yield: 64.66%; Brown solid; mp 122-123 °C ; **^{1}H NMR** (400 MHz,$CDCl_3$) δ 9.14 (s, 1H), 8.59 (s, 1H), 8.53 (d, *J* = 8.1 Hz, 1H), 8.29 (d, *J* = 2.5 Hz, 1H), 8.03 (d, *J* = 8.1 Hz, 1H), 7.97 (d, *J* = 8.1 Hz, 1H), 7.83 (dd, *J* = 8.5, 2.6 Hz, 1H), 7.76 (t, *J* = 7.5 Hz, 1H), 7.70 (t, *J* = 7.5 Hz, 1H), 7.62 (d, *J* = 8.4 Hz, 2H), 7.22 (d, *J* = 8.4 Hz, 2H), 6.96 (d, *J* = 8.4 Hz, 1H), 5.40 (q, *J* = 7.2 Hz, 1H), 1.68 (d, *J* = 7.0 Hz, 3H); **^{13}C NMR** (100 MHz, $CDCl_3$) δ 164.3, 162.2, 157.2, 151.3, 145.8, 143.4, 138.4, 136.1, 134.9,

131.3, 129.9, 129.1, 128.3, 127.8, 127.1, 127.1, 121.2, 120.7, 112.2, 46.5, 22.0; **HRMS** (ESI-TOF) m/z: $[M+H]^+$ calcd for $C_{24}H_{19}F_3N_3O_2$, 438.1429, found 438.1429.

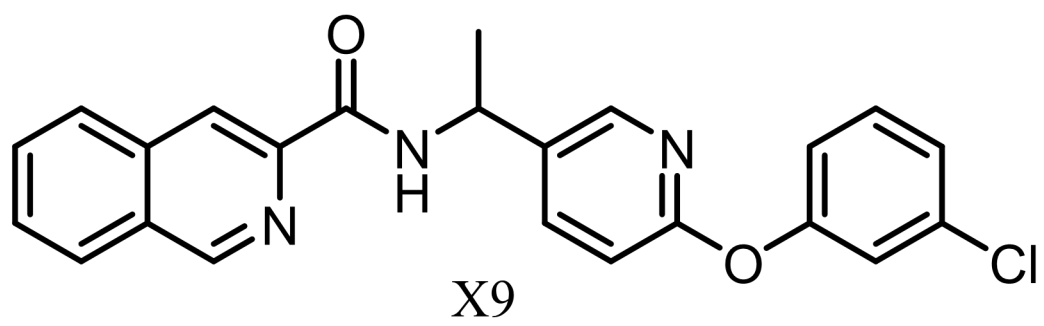

X9

***N*-(1-(6-(3-chlorophenoxy)pyridin-3-yl)ethyl)isoquinoline-3-carboxamiden (X9)**

Yield: 74.43%; White solid; mp 130-131°C ; ^{1}**H NMR** (400 MHz, DMSO-d_6) δ 9.37 (s, 1H), 9.31 (d, J = 8.4 Hz, 1H), 8.53 (s, 1H), 8.27 – 8.19 (m, 2H), 8.14 (d, J = 8.0 Hz, 1H), 8.01 (dd, J = 8.5, 2.5 Hz, 1H), 7.86 – 7.80 (m, 1H), 7.79 – 7.74 (m, 1H), 7.39 (t, J = 8.1 Hz, 1H), 7.26 – 7.17 (m, 2H), 7.05 (t, J = 8.0 Hz, 2H), 5.29 (q, J = 7.2 Hz, 1H), 1.59 (d, J = 7.1 Hz, 3H); ^{13}**C NMR** (100 MHz, DMSO) δ 163.6, 161.5, 154.9, 151.5, 145.5, 143.7, 138.7, 135.6, 135.4, 133.5, 131.3, 131.0, 129.3, 129.1, 127.9, 127.8, 124.4, 121.2, 120.0, 119.8, 111.5, 45.9, 21.5; **HRMS** (ESI-TOF) m/z: $[M+H]^+$ calcd for $C_{23}H_{19}ClN_3O_2$, 404.1166, found 404.1163.

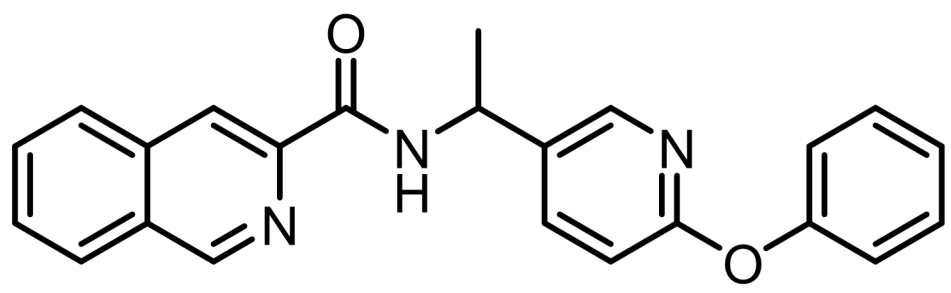

X10

***N*-(1-(6-phenoxypyridin-3-yl)ethyl)isoquinoline-3-carboxamide (X10)**

Yield: 80.21%; White solid; mp 120-121°C ; ^{1}**H NMR** (400 MHz, DMSO-d_6) δ 9.40 (s, 1H), 9.32 (d, J = 8.5 Hz, 1H), 8.54 (s, 1H), 8.26 (d, J = 8.1 Hz, 1H), 8.23 – 8.15 (m, 2H), 7.99 (d, J = 8.5 Hz, 1H), 7.87 (t, J = 7.4 Hz, 1H), 7.81 (t, J = 7.4 Hz, 1H), 7.39 (t, J = 7.6 Hz, 2H), 7.19 (t, J = 7.4 Hz, 1H), 7.09 (d, J = 8.0 Hz, 2H), 7.00 (d, J = 8.5 Hz, 1H), 5.26 (q, J = 7.4 Hz, 1H), 1.58 (d, J = 7.0 Hz, 3H); ^{13}**C NMR** (100 MHz, DMSO) δ 163.4, 162.0, 153.9, 151.5, 145.5, 143.7, 138.4, 135.3, 135.0, 131.3, 129.6, 129.2, 129.1, 127.9, 127.8, 124.3, 120.9, 119.9, 111.1, 45.8, 21.5; **HRMS** (ESI-TOF) m/z: $[M+H]^+$ calcd for $C_{23}H_{20}N_3O_2$, 370.1556, found 370.1556.

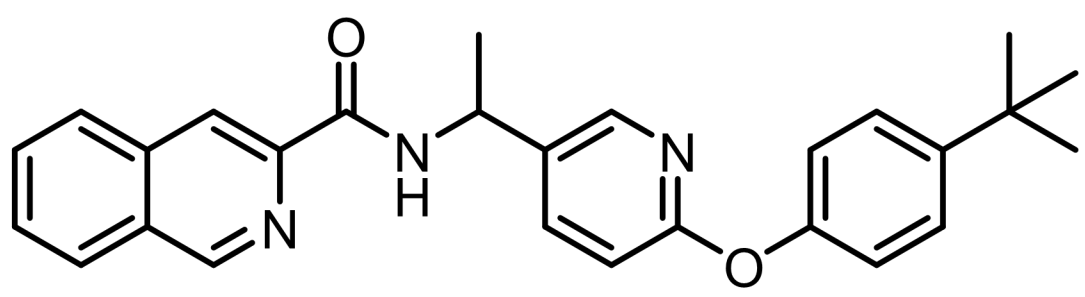

X11

***N*-(1-(6-(4-(tert-butyl)phenoxy)pyridin-3-yl)ethyl)isoquinoline-3-carboxamide (X11)**

Yield: 74.13%; White solid; mp 140-141°C ; ^{1}**H NMR** (400 MHz, DMSO-d_6) δ 9.37 (s, 1H), 9.31 (d, *J* = 8.4 Hz, 1H), 8.57 (s, 1H), 8.30 – 8.17 (m, 2H), 8.14 (d, *J* = 8.1 Hz, 1H), 7.98 (dd, *J* = 8.5, 2.5 Hz, 1H), 7.81 (t, *J* = 7.5 Hz, 1H), 7.74 (t, *J* = 6.9 Hz, 1H), 7.33 (d, *J* = 8.8 Hz, 2H), 6.97 (t, *J* = 8.8 Hz, 3H), 5.31 (p, *J* = 7.3 Hz, 1H), 1.59 (d, *J* = 7.0 Hz, 3H), 1.21 (s, 9H); ^{13}**C NMR** (100 MHz, DMSO) δ 163.4, 162.2, 151.6, 151.4, 146.5, 145.5, 143.7, 138.3, 135.3, 134.8, 131.2, 129.2, 129.0, 127.9, 127.7, 126.2, 120.5, 119.9, 111.0, 45.8, 34.0, 31.1, 21.5; **HRMS** (ESI-TOF) m/z: $[M+H]^+$ calcd for $C_{27}H_{28}N_3O_2$, 426.2182, found 426.2172.

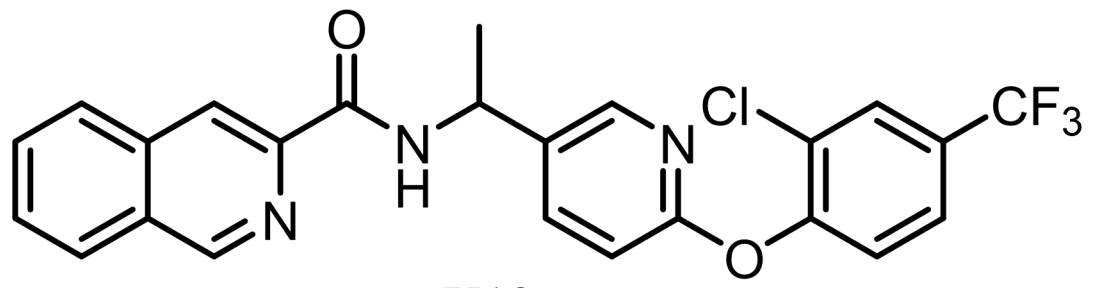

X12

***N*-(1-(6-(2-chloro-4-(trifluoromethyl)phenoxy)pyridin-3-yl)ethyl)isoquinoline-3-carboxamide (X12)**

Yield: 56.87%; Brown solid; mp 142-143°C ; ^{1}**H NMR** (400 MHz, DMSO-d_6) δ 9.39 (d, *J* = 9.1 Hz, 2H), 8.55 – 8.49 (m, 2H), 8.25 (d, *J* = 8.2 Hz, 1H), 8.17 (d, *J* = 8.3 Hz, 1H), 7.97 (dd, *J* = 8.3, 2.6 Hz, 1H), 7.85 (d, *J* = 6.8 Hz, 1H), 7.80 (t, *J* = 6.9 Hz, 1H), 7.48 (d, *J* = 8.3 Hz, 1H), 5.33 – 5.26 (m, 1H), 1.59 (d, *J* = 7.1 Hz, 3H); ^{13}**C NMR** (100 MHz, DMSO) δ 163.7, 151.6, 151.4, 148.6, 148.4, 148.2, 143.6, 139.5, 137.9, 135.4, 131.4, 129.3, 129.2, 128.0, 127.8, 124.0, 120.1, 120.0, 45.9, 21.4. ^{19}**F NMR** (376 MHz, DMSO) δ -55.9, -55.9, -56.0; **HRMS** (ESI-TOF) m/z: $[M+H]^+$ calcd for $C_{24}H_{18}ClF_3N_3O_2$, 472.1040, found 472.1037.

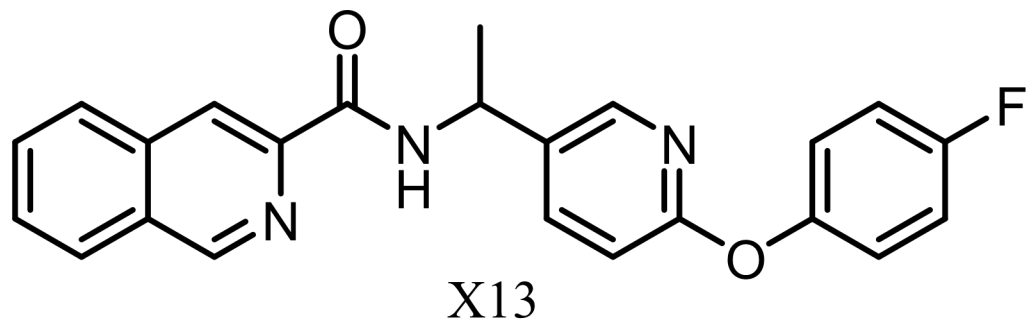

X13

***N*-(1-(6-(4-fluorophenoxy)pyridin-3-yl)ethyl)isoquinoline-3-carboxamide (X13)**

Yield: 70.55%; Gray solid; mp 175-176°C ; ^{1}**H NMR** (400 MHz, DMSO-d_6) δ 9.40 (s, 1H), 9.34 (d, *J* = 8.5 Hz, 1H), 8.54 (s, 1H), 8.27 – 8.22 (m, 2H), 8.17 (d, *J* = 8.2 Hz, 1H), 8.03 (d, *J* = 8.5 Hz, 1H), 7.88 – 7.82 (m, 1H), 7.82 – 7.76 (m, 1H), 7.41 (q, *J* = 8.6 Hz, 1H), 7.09 – 7.01 (m, 3H), 6.95 (d, *J* = 8.4 Hz, 1H), 5.29 (p, *J* = 7.3 Hz, 1H), 1.59 (d, *J* = 7.0 Hz, 3H); ^{13}**C NMR** (100 MHz, DMSO) δ 163.7, 163.5, 161.4, 161.3, 155.2, 151.5, 145.5, 143.6, 138.6, 135.5, 135.3, 131.3, 130.8, 130.7, 129.2, 129.1,

127.9, 127.7, 119.9, 117.0, 111.4, 111.2, 111.0, 108.7, 108.5, 45.8, 21.5; **^{19}F NMR** (376 MHz, DMSO) δ -118.8; **HRMS** (ESI-TOF) m/z: $[M+H]^+$ calcd for $C_{23}H_{19}FN_3O_2$, 388.1461, found 388.1455.

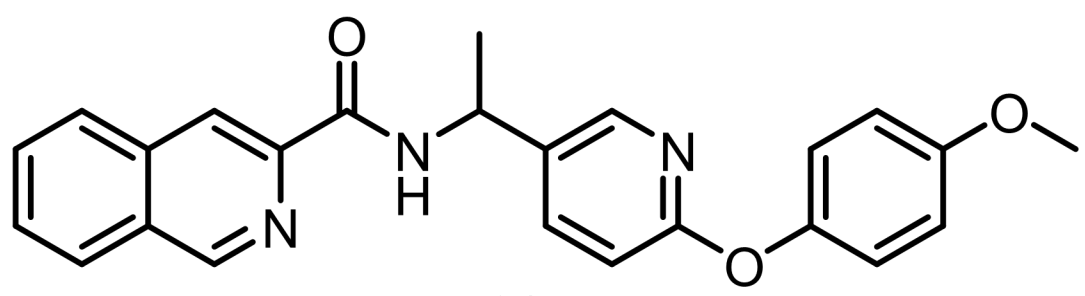

X14

***N*-(1-(6-(4-methoxyphenoxy)pyridin-3-yl)ethyl)isoquinoline-3-carboxamide (X14)**

Yield: 74.13%; White solid; mp 160-161°C ; **^{1}H NMR** (400 MHz, DMSO-d_6) δ 9.48 – 9.20 (m, 2H), 8.54 (s, 1H), 8.30 – 8.09 (m, 3H), 7.96 (s, 1H), 7.88 – 7.71 (m, 2H), 7.11 – 6.87 (m, 5H), 5.39 – 5.17 (m, 1H), 3.73 (s, 3H), 1.58 (d, J = 7.0 Hz, 3H); **^{13}C NMR** (100 MHz, DMSO) δ 163.4, 162.6, 156.0, 151.4, 145.4, 143.6, 138.1, 135.3, 134.5, 131.2, 129.2, 129.0, 127.8, 127.7, 122.2, 119.8, 114.6, 110.5, 55.3, 45.8, 21.4; **HRMS** (ESI-TOF) m/z: $[M+H]^+$ calcd for $C_{24}H_{22}N_3O_3$, 400.1661, found 400.1660.

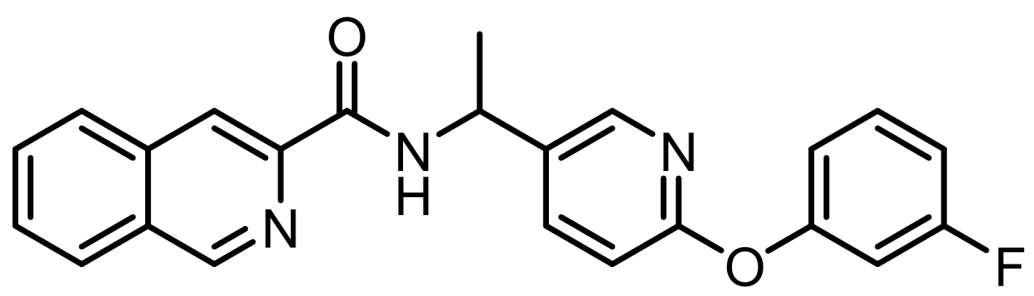

X15

***N*-(1-(6-(3-fluorophenoxy)pyridin-3-yl)ethyl)isoquinoline-3-carboxamide (X15)**

Yield: 50.61%; Brown solid; mp 141-142°C ; **^{1}H NMR** (400 MHz, DMSO-d_6) δ 9.39 (s, 1H), 9.29 (d, J = 8.4 Hz, 1H), 8.54 (s, 1H), 8.25 (d, J = 9.0 Hz, 2H), 8.17 (d, J = 8.1 Hz, 1H), 8.02 (dd, J = 8.5, 2.5 Hz, 1H), 7.89 – 7.83 (m, 1H), 7.80 (t, J = 7.5 Hz, 1H), 7.45 – 7.38 (m, 1H), 7.04 (t, J = 9.7 Hz, 3H), 6.95 (d, J = 9.2 Hz, 1H), 5.29 (p, J = 7.2 Hz, 1H), 1.59 (d, J = 7.0 Hz, 3H); **^{13}C NMR** (100 MHz, DMSO) δ 163.8, 163.5, 161.5, 161.3, 155.3, 155.2, 151.5, 145.5, 143.7, 138.6, 135.6, 135.4, 131.3, 130.8, 130.7, 129.2, 129.1, 127.9, 127.8, 120.0, 117.0, 117.0, 111.4, 111.3, 111.0, 108.8, 108.5, 45.8, 21.5; **^{19}F NMR** (376 MHz, DMSO) δ -111.5; **HRMS** (ESI-TOF) m/z: $[M+H]^+$ calcd for $C_{23}H_{19}FN_3O_2$, 388.1461, found 388.1461.

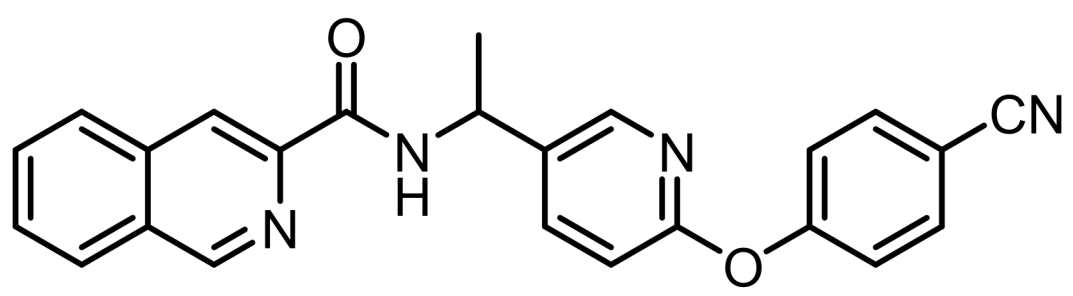

X16

***N*-(1-(6-(4-cyanophenoxy)pyridin-3-yl)ethyl)isoquinoline-3-carboxamide (X16)**

Yield: 70.72%; White solid; mp 155-156°C ; **^{1}H NMR** (400 MHz, DMSO-d_6) δ 9.45 – 9.27 (m, 2H), 8.54 (s, 1H), 8.30 (d, J = 2.4 Hz, 1H), 8.22 (d, J = 8.1 Hz, 1H), 8.14 (d, J = 8.1 Hz, 1H), 8.06 (dd, J =

8.5, 2.5 Hz, 1H), 7.90 – 7.71 (m, 4H), 7.26 (d, *J* = 8.7 Hz, 2H), 7.12 (d, *J* = 8.5 Hz, 1H), 5.38 – 5.26 (m, 1H), 1.60 (d, *J* = 7.1 Hz, 3H); **^{13}C NMR** (100 MHz, DMSO) δ 163.5, 160.7, 158.0, 151.4, 145.7, 143.6, 138.8, 136.3, 135.3, 134.1, 131.2, 129.2, 129.0, 127.9, 127.7, 121.3, 119.9, 118.5, 112.1, 106.5, 45.8, 21.4; **HRMS** (ESI-TOF) m/z: $[M+H]^+$ calcd for $C_{24}H_{19}N_4O_2$, 395.1508, found 395.1511.

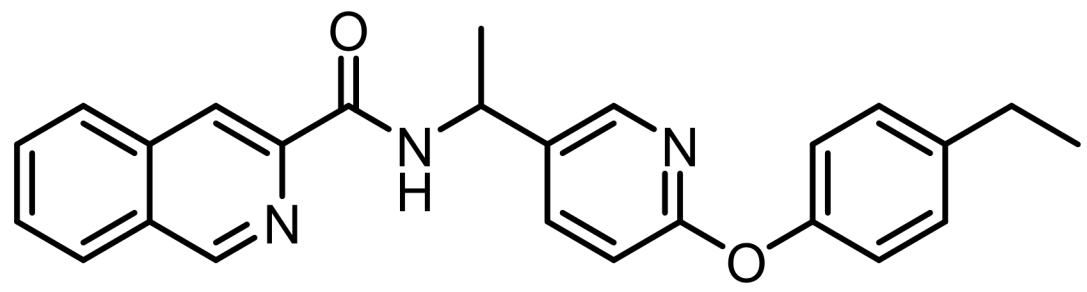

X17

***N*-(1-(6-(4-ethylphenoxy)pyridin-3-yl)ethyl)isoquinoline-3-carboxamide (X17)**

Yield: 80.86%; White solid; mp 130-131°C ; **^{1}H NMR** (400 MHz, DMSO-*d*$_6$) δ 9.37 (s, 1H), 9.29 (d, *J* = 8.4 Hz, 1H), 8.55 (s, 1H), 8.29 – 8.16 (m, 2H), 8.13 (d, *J* = 8.1 Hz, 1H), 7.97 (dd, *J* = 8.5, 2.5 Hz, 1H), 7.81 (t, *J* = 6.8 Hz, 1H), 7.78 – 7.69 (m, 1H), 7.16 (d, *J* = 8.5 Hz, 2H), 7.04 – 6.90 (m, 3H), 5.29 (p, *J* = 7.2 Hz, 1H), 2.53 (q, *J* = 7.6 Hz, 2H), 1.58 (d, *J* = 7.0 Hz, 3H), 1.12 (t, *J* = 7.6 Hz, 3H); **^{13}C NMR** (100 MHz, DMSO) δ 163.4, 162.2, 151.9, 151.4, 145.4, 143.6, 139.7, 138.2, 135.3, 134.7, 131.2, 129.2, 129.0, 128.7, 127.8, 127.7, 120.8, 119.9, 110.9, 45.8, 27.4, 21.4, 15.5; **HRMS** (ESI-TOF) m/z: $[M+H]^+$ calcd for $C_{25}H_{24}N_3O_2$, 398.1869, found 398.1867.

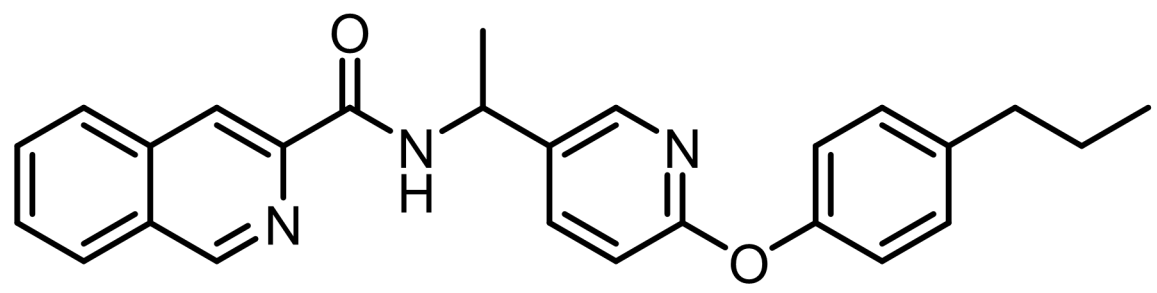

X18

***N*-(1-(6-(4-propylphenoxy)pyridin-3-yl)ethyl)isoquinoline-3-carboxamide (X18)**

Yield: 76.52%; White solid; mp 136-137°C ; **^{1}H NMR** (400 MHz, DMSO-*d*$_6$) δ 9.38 (s, 1H), 9.29 (d, *J* = 8.4 Hz, 1H), 8.56 (s, 1H), 8.27 – 8.19 (m, 2H), 8.15 (d, *J* = 8.2 Hz, 1H), 7.98 (dd, *J* = 8.5, 2.5 Hz, 1H), 7.82 (d, *J* = 7.9 Hz, 1H), 7.76 (t, *J* = 7.5 Hz, 1H), 7.14 (d, *J* = 8.4 Hz, 2H), 7.02 – 6.92 (m, 3H), 5.30 (p, *J* = 7.2 Hz, 1H), 2.48 (t, *J* = 7.5 Hz, 2H), 1.59 (d, *J* = 7.1 Hz, 3H), 1.52 (q, *J* = 7.5 Hz, 2H), 0.84 (t, *J* = 7.1 Hz, 3H); **^{13}C NMR** (100 MHz, DMSO) δ 163.4, 162.2, 152.0, 151.4, 145.5, 143.6, 138.2, 138.0, 135.3, 134.7, 131.2, 129.2, 129.0, 127.8, 127.7, 120.6, 119.8, 110.9, 45.8, 36.5, 24.0, 21.4, 13.5; **HRMS** (ESI-TOF) m/z: $[M+H]^+$ calcd for $C_{26}H_{26}N_3O_2$, 412.2025, found 412.2027.

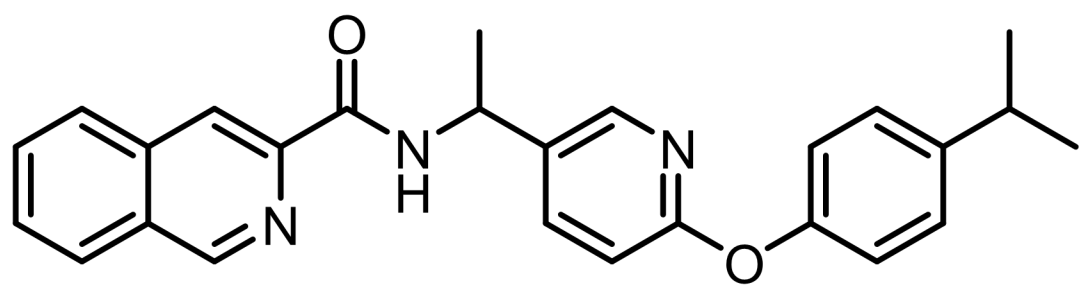

X19

N-(1-(6-(4-isopropylphenoxy)pyridin-3-yl)ethyl)isoquinoline-3-carboxamide (X19)

Yield: 69.20%; White solid; mp 122-123°C ; ^{1}**H NMR** (400 MHz, DMSO-d_6) δ 9.38 (s, 1H), 9.29 (d, *J* = 8.7 Hz, 1H), 8.55 (s, 1H), 8.23 (d, *J* = 6.6 Hz, 2H), 8.16 (d, *J* = 8.1 Hz, 1H), 7.98 (dd, *J* = 8.6, 2.4 Hz, 1H), 7.87 – 7.81 (m, 1H), 7.81 – 7.74 (m, 1H), 7.26 – 7.17 (m, 2H), 7.03 – 6.91 (m, 3H), 5.34 – 5.22 (m, 1H), 1.58 (d, *J* = 6.5 Hz, 3H), 1.16 (dd, *J* = 7.0, 1.8 Hz, 6H); ^{13}**C NMR** (100 MHz, DMSO) δ 163.4, 162.2, 151.9, 151.4, 145.4, 144.3, 143.6, 138.2, 135.3, 134.7, 131.2, 129.2, 129.0, 127.9, 127.7, 127.2, 120.7, 119.8, 110.9, 45.8, 32.7, 23.8, 21.4; **HRMS** (ESI-TOF) m/z: $[M+H]^+$ calcd for $C_{26}H_{26}N_3O_2$, 412.2025, found 412.2023.

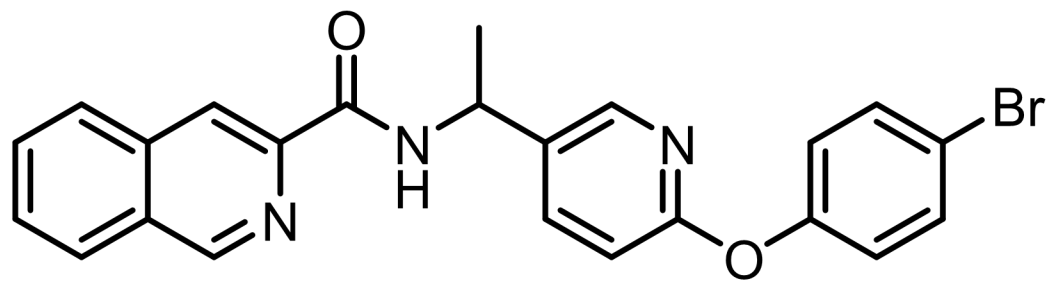

X20

N-(1-(6-(4-bromophenoxy)pyridin-3-yl)ethyl)isoquinoline-3-carboxamide (X20)

Yield: 70.05%; Bright yellow solid; mp 145-146°C ; ^{1}**H NMR** (400 MHz, DMSO-d_6) δ 9.38 (s, 1H), 9.30 (d, *J* = 8.5 Hz, 1H), 8.54 (s, 1H), 8.29 – 8.19 (m, 2H), 8.16 (d, *J* = 8.2 Hz, 1H), 8.06 – 7.96 (m, 1H), 7.89 – 7.74 (m, 2H), 7.54 (d, *J* = 8.8 Hz, 2H), 7.15 – 6.97 (m, 3H), 5.28 (p, *J* = 7.2 Hz, 1H), 1.58 (d, *J* = 7.1 Hz, 3H); ^{13}**C NMR** (100 MHz, DMSO) δ 163.4, 161.5, 153.3, 151.4, 145.4, 143.6, 138.5, 135.3, 132.3, 131.3, 129.2, 129.0, 127.9, 127.7, 123.3, 123.2, 119.9, 116.2, 111.3, 45.8, 21.4; **HRMS** (ESI-TOF) m/z: $[M+H]^+$ calcd for $C_{23}H_{19}BrN_3O_2$, 448.0661, found 448.0669.

4.Crystal data of the target compound X9

Table S2 Crystal data and structure refinement for **X9**.

Identification code	1
Empirical formula	$C_{23}H_{18}ClN_3O_2$
Formula weight	403.85
Temperature/K	296.15
Crystal system	monoclinic
Space group	$P2_1/n$
a/Å	5.7311(9)
b/Å	15.323(2)
c/Å	22.011(4)
α/°	90
β/°	95.178(3)
γ/°	90
Volume/Å^3	1925.0(5)
Z	4
ρ_{calc}g/cm^3	1.393
μ/mm^{-1}	0.224
F(000)	840.0
Crystal size/mm^3	0.26 × 0.18 × 0.16
Radiation	MoKα (λ = 0.71073)
2Θ range for data collection/°	5.318 to 49.996
Index ranges	$-6 \le h \le 6$, $-16 \le k \le 18$, $-26 \le l \le 25$
Reflections collected	9688
Independent reflections	3389 [R_{int} = 0.0494, R_{sigma} = 0.0594]
Data/restraints/parameters	3389/0/263
Goodness-of-fit on F^2	0.977
Final R indexes [I>=2σ (I)]	R_1 = 0.0491, wR_2 = 0.1010
Final R indexes [all data]	R_1 = 0.0989, wR_2 = 0.1142
Largest diff. peak/hole / e Å^{-3}	0.21/-0.28

Table S3. Fractional Atomic Coordinates (×10^4) and Equivalent Isotropic Displacement Parameters (Å^2×10^3) for 1. U_{eq} is defined as 1/3 of of the trace of the orthogonalised U_{IJ} tensor.

**Atom**	***x***	***y***	***z***	**U(eq)**
Cl1	2515.9(15)	7504.6(5)	2554.1(3)	71.3(3)
O1	7400(3)	5667.2(11)	4118.3(9)	52.9(5)
O2	2626(3)	1884.3(12)	4653.2(8)	51.6(5)
N1	5021(3)	4488.8(13)	3853.7(9)	36.6(5)
N3	7182(3)	675.7(13)	5476.6(9)	38.2(5)
N2	6349(4)	1609.8(12)	4453.2(9)	37.9(5)
C10	6597(4)	3031.9(16)	3945.6(10)	32.1(6)
C15	5104(4)	1120.8(14)	5403.9(10)	31.7(6)
C11	4820(4)	3617.3(16)	3799.3(10)	35.5(6)
C17	4206(4)	805.2(15)	6424.0(11)	35.0(6)
C7	7094(4)	4786.8(17)	4059.3(11)	35.8(6)
C12	6200(4)	2057.7(15)	3867.3(10)	35.2(6)
C16	3614(4)	1181.1(15)	5847.6(11)	36.8(6)
C14	4565(4)	1566.7(15)	4804.6(11)	35.9(6)
C22	6341(4)	336.0(15)	6507.3(11)	35.6(6)
C9	8764(4)	3382.7(17)	4163.0(11)	38.6(6)
C8	9031(4)	4266.8(16)	4220.2(11)	39.7(6)
C5	5429(4)	6190.0(16)	3976.5(12)	38.9(6)
C1	3093(5)	7054.7(16)	3275.0(11)	41.6(7)
C23	7704(4)	293.2(16)	6002.8(11)	40.7(7)
C6	5046(4)	6538.7(16)	3404.4(12)	41.1(7)
C2	1576(5)	7223.1(16)	3708.4(12)	45.1(7)
C21	7036(5)	-43.3(17)	7077.1(12)	46.5(7)
C13	7892(5)	1648.8(17)	3458.6(12)	49.6(7)
C18	2849(5)	878.0(17)	6925.9(12)	46.2(7)
C4	3958(5)	6367.3(17)	4422.7(12)	47.7(7)
C19	3587(5)	514.7(18)	7473.5(12)	52.5(8)
C3	2021(5)	6884.9(17)	4281.4(13)	51.3(7)
C20	5703(5)	50.9(19)	7552.9(12)	53.6(8)

Table S4. Hydrogen Bond Lengths (Å) and Bond Angles (°)

Donor–H···Acceptor	D–H	H···A	D···A	D–H···A
N(2)–H(2)···N(3)	0.86	2.27	2.6752	109
C(9)–H(9)···O(2)	0.93	2.39	3.3020	167

5. NMR and HRMS spectrum of the target compounds

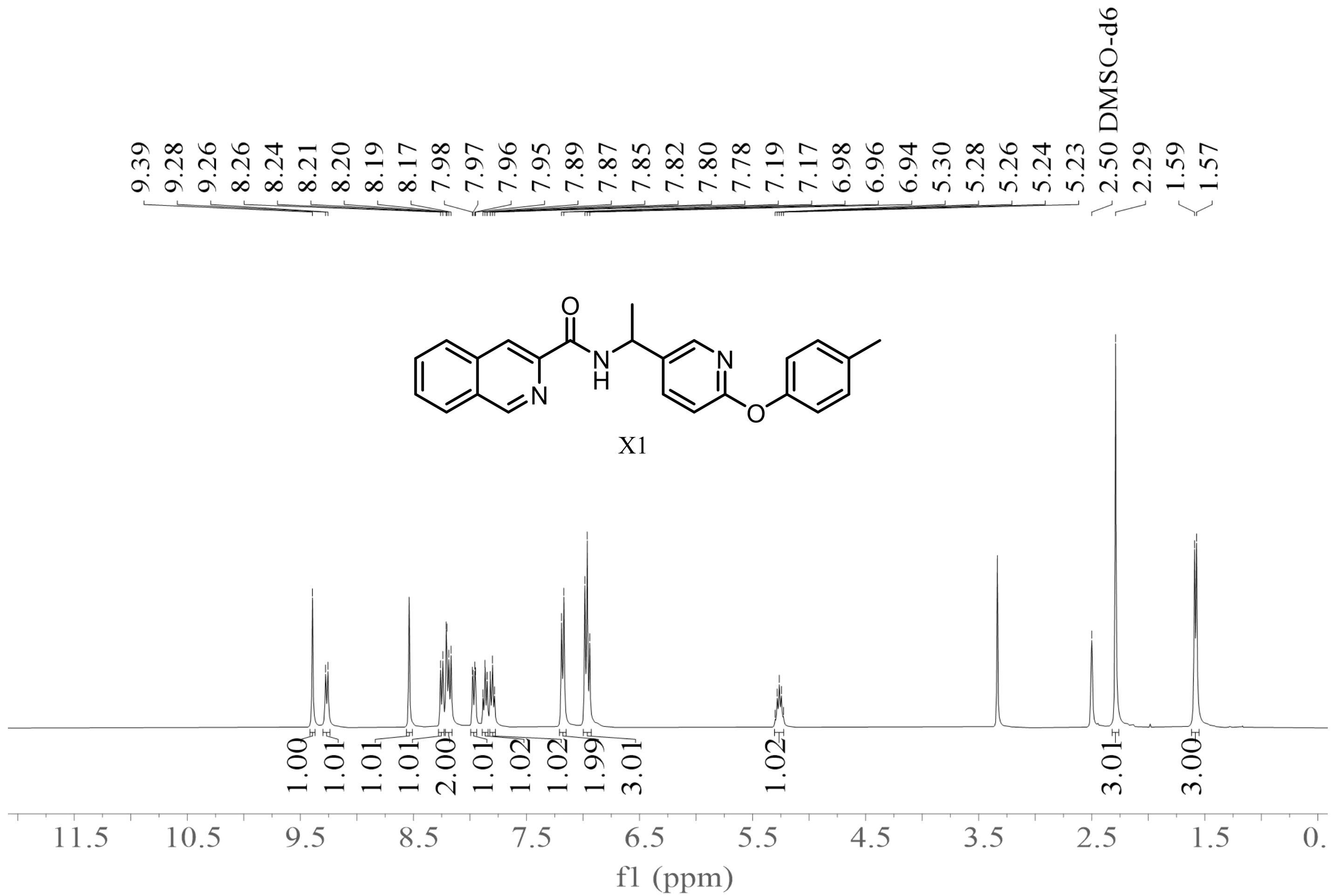

¹H NMR spectrum of the compound X1

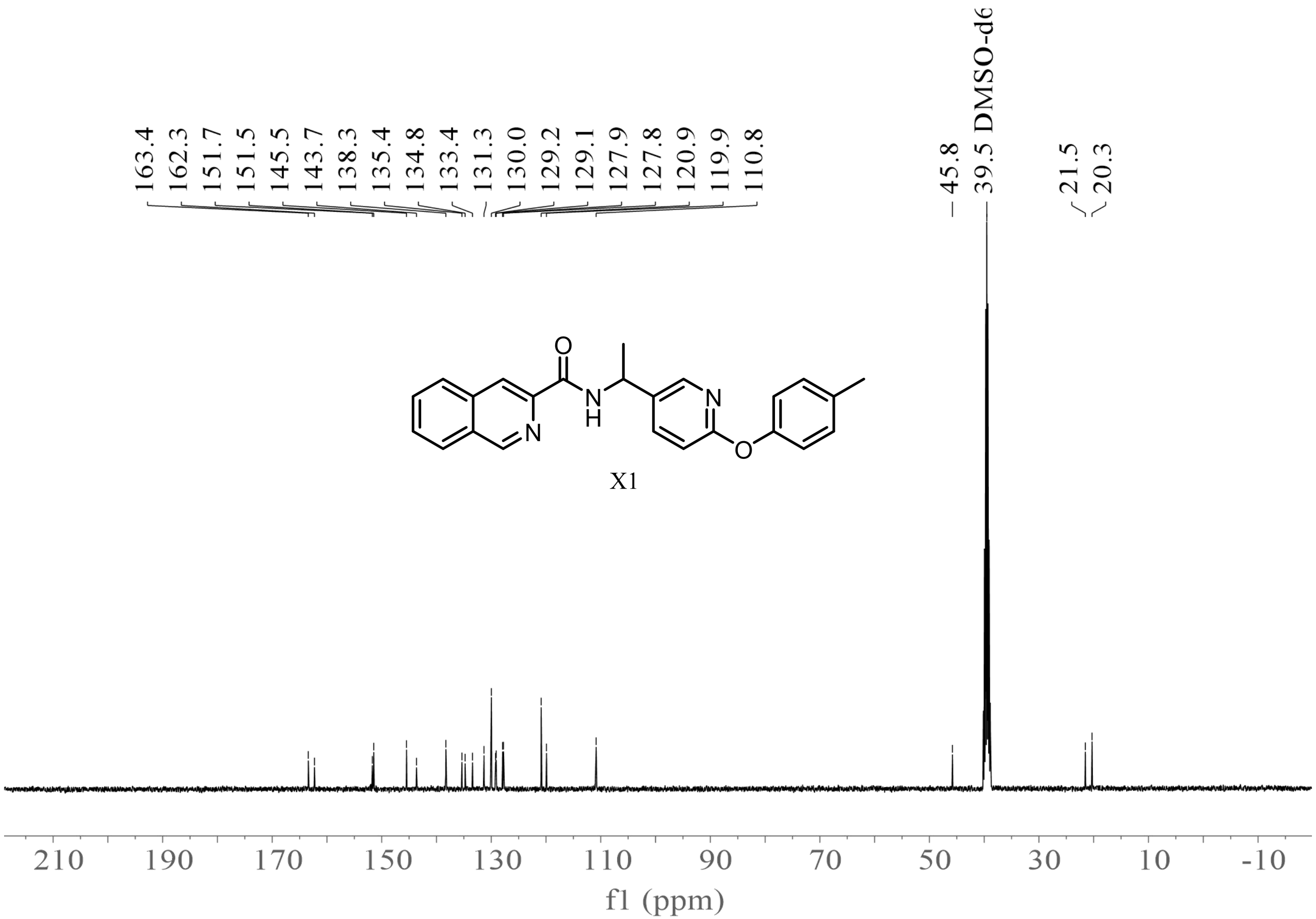

^{13}C NMR spectrum of the compound X1

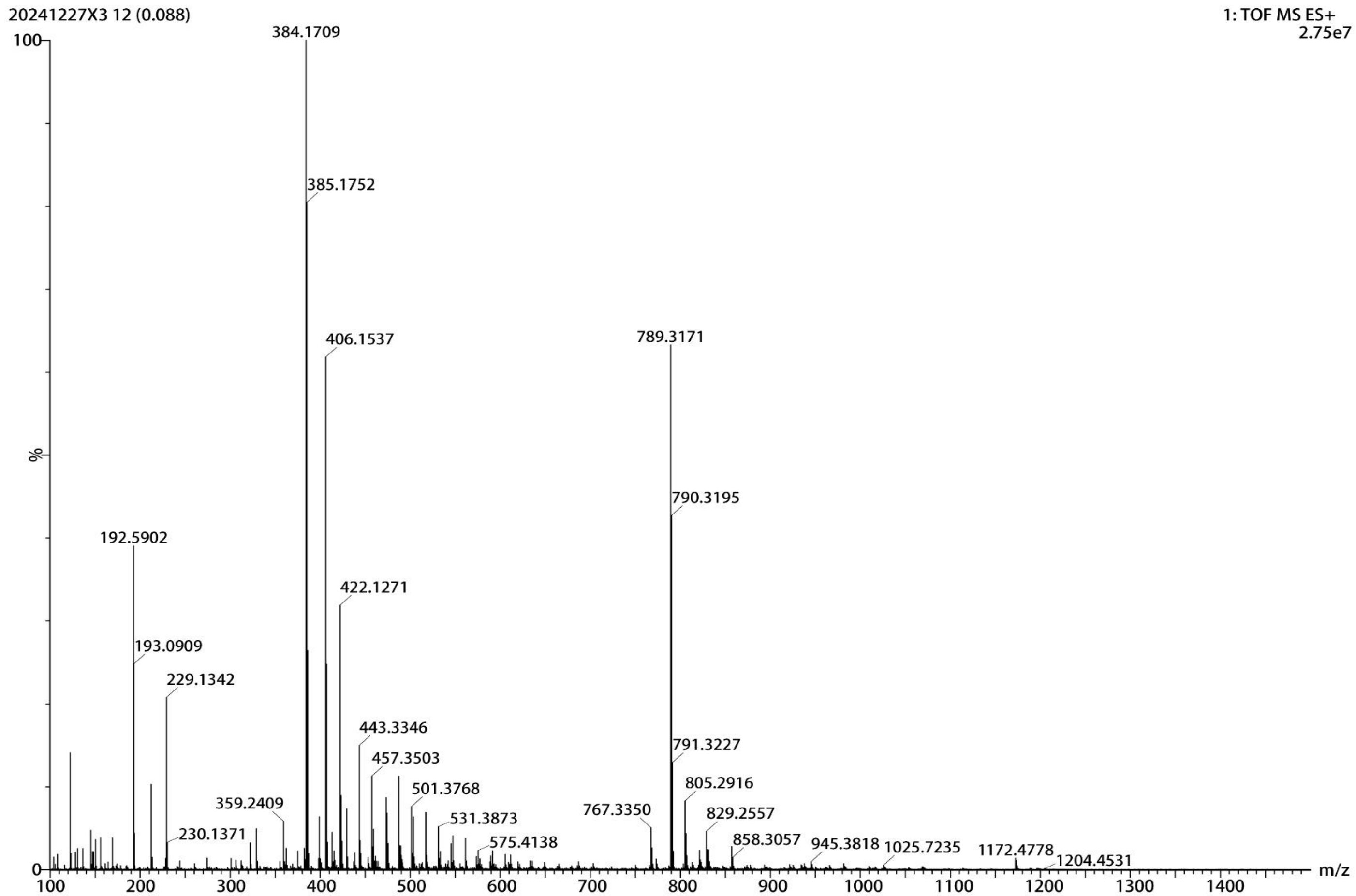

ESI-MS spectrum of the compound X1

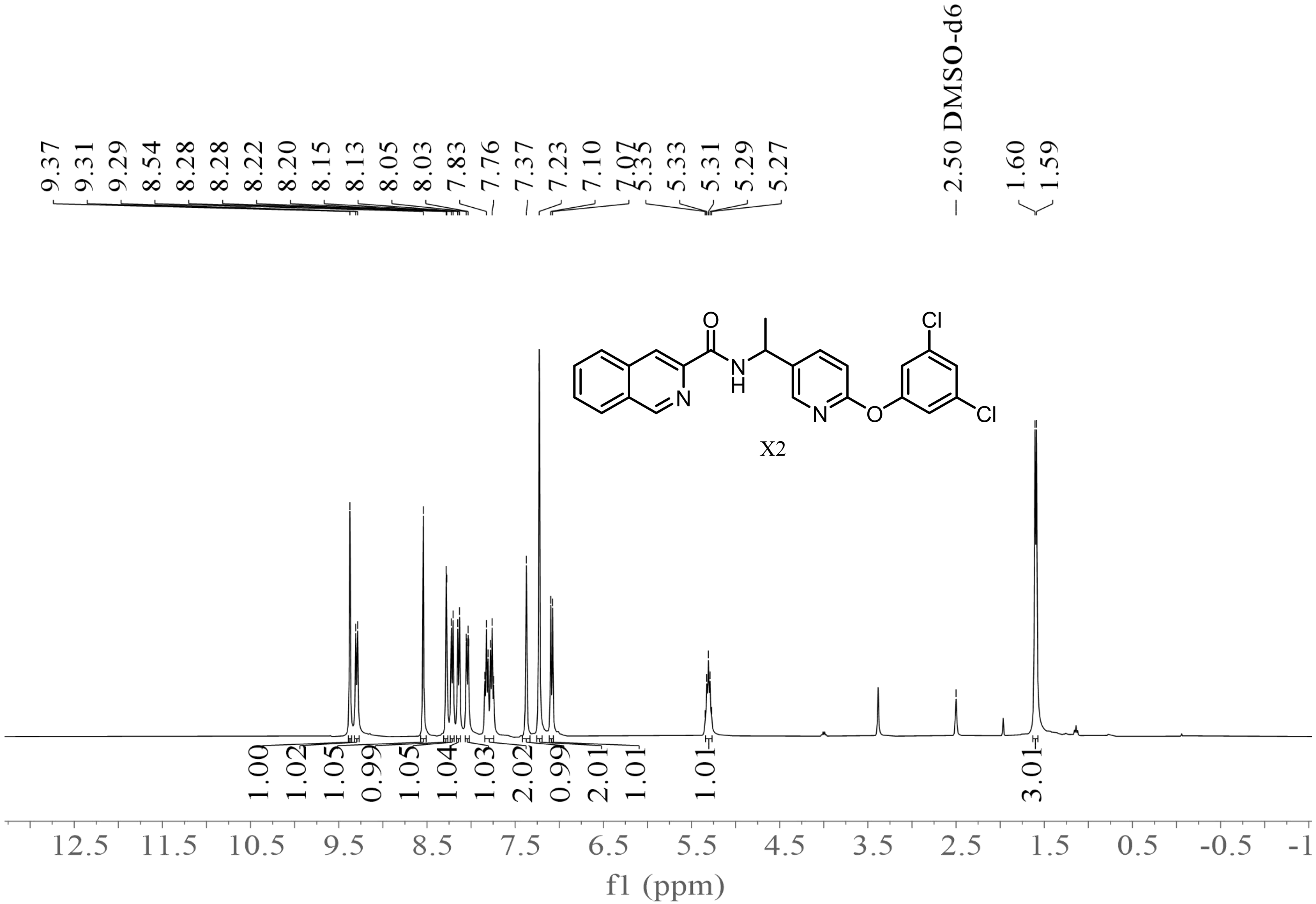

^{1}H NMR spectrum of the compound X2

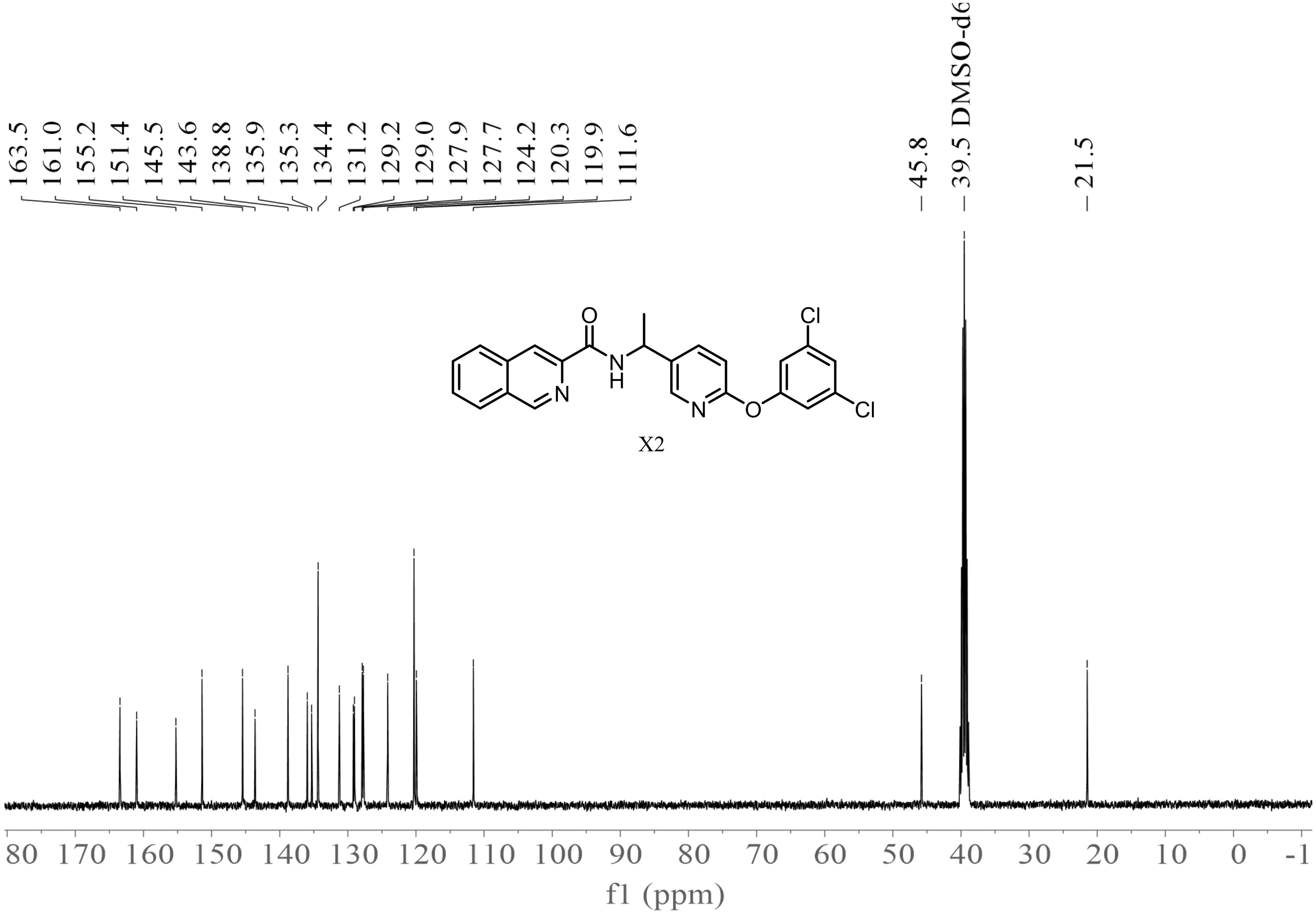

^{13}C NMR spectrum of the compound X2

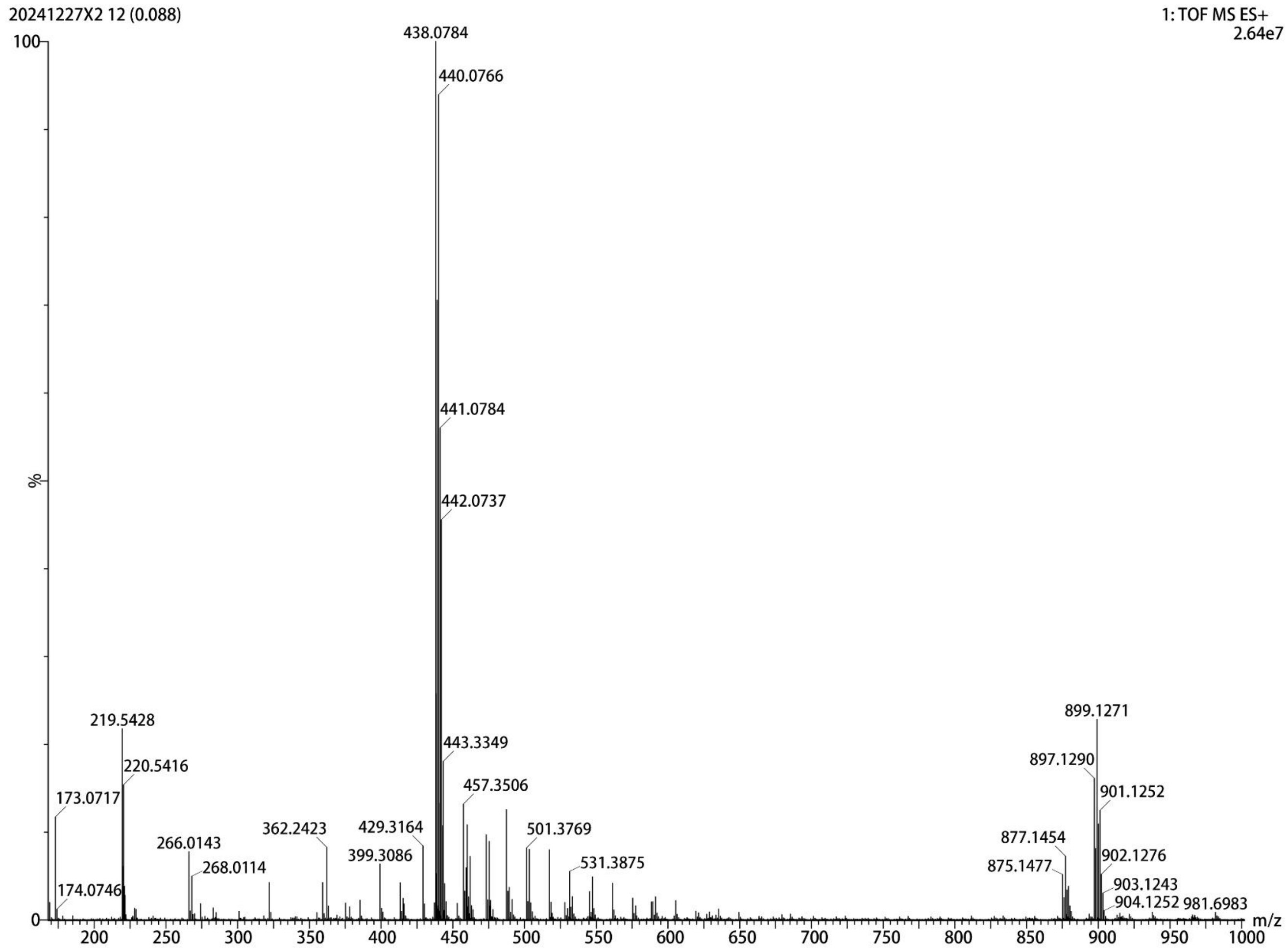

ESI-MS spectrum of the compound X2

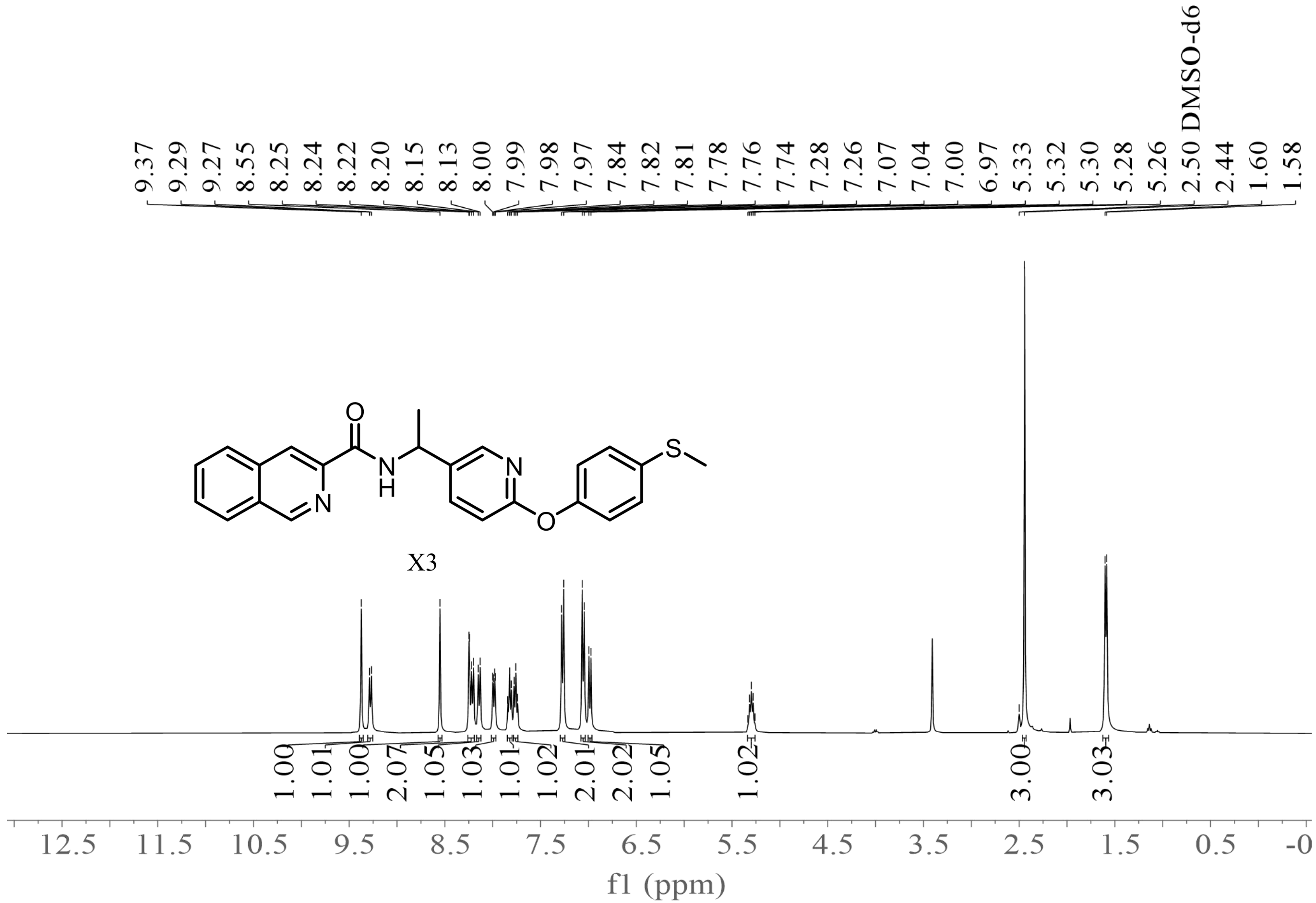

^{1}H NMR spectrum of the compound X3

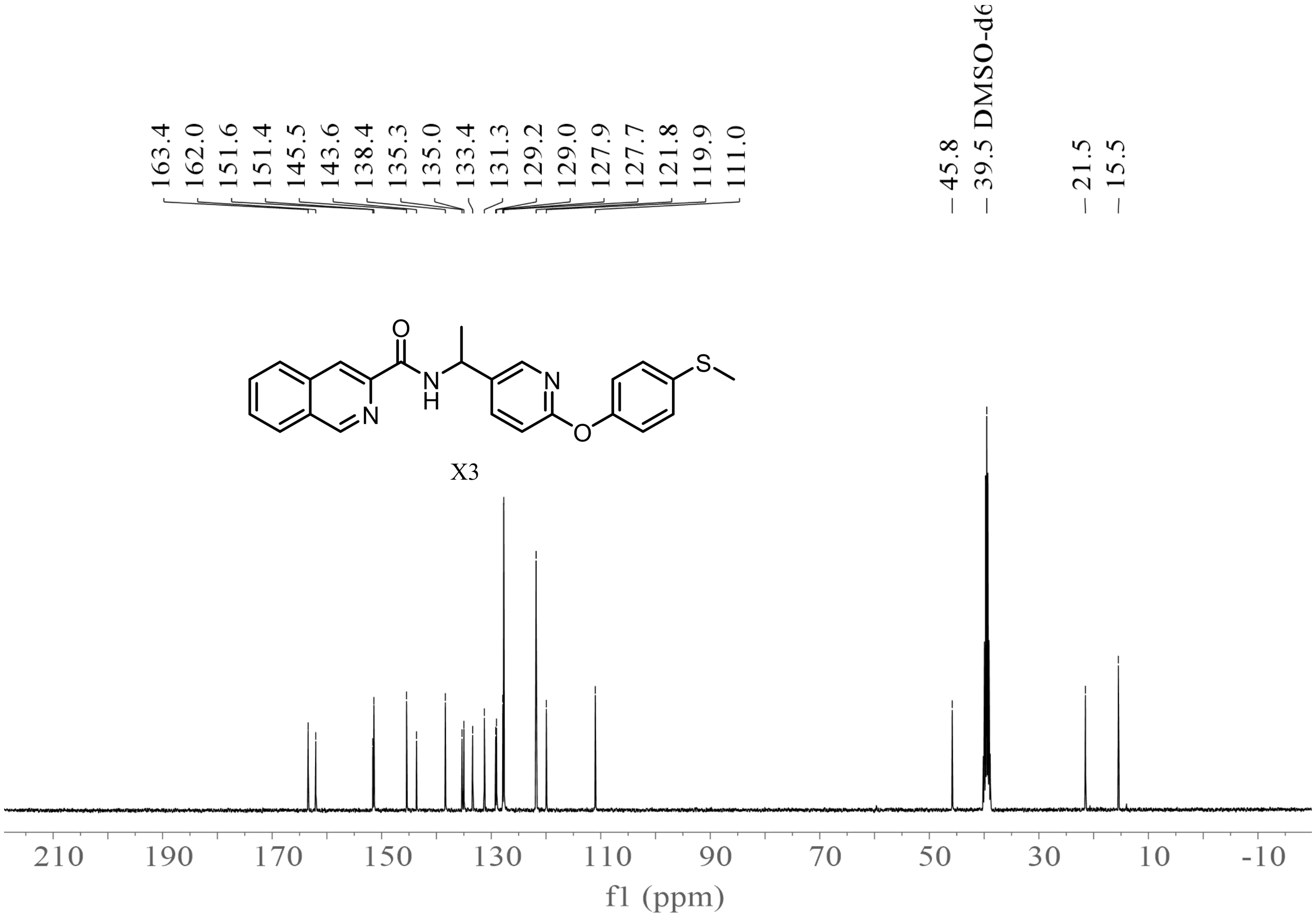

^{13}C NMR spectrum of the compound X3

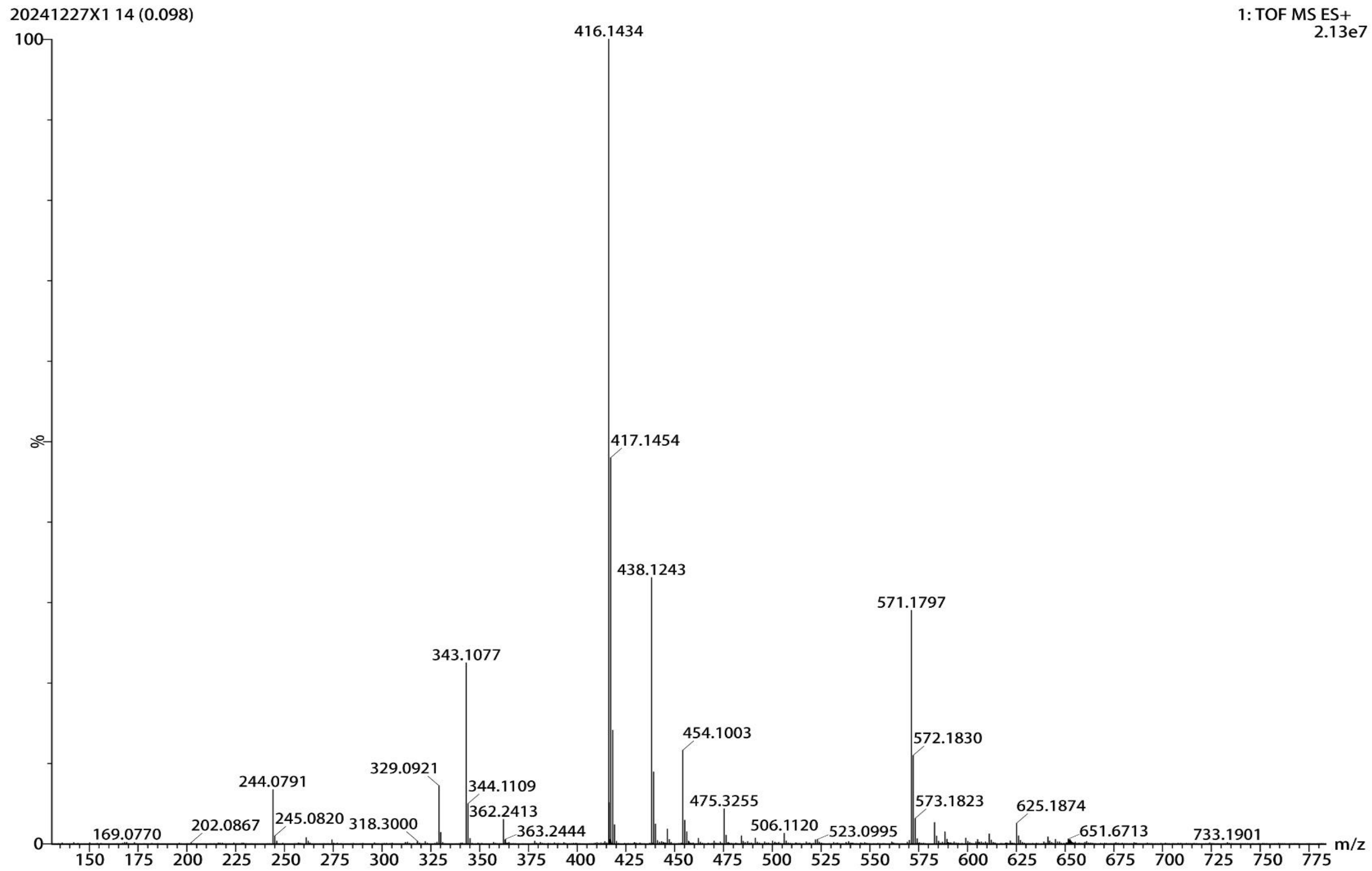

ESI-MS spectrum of the compound X3

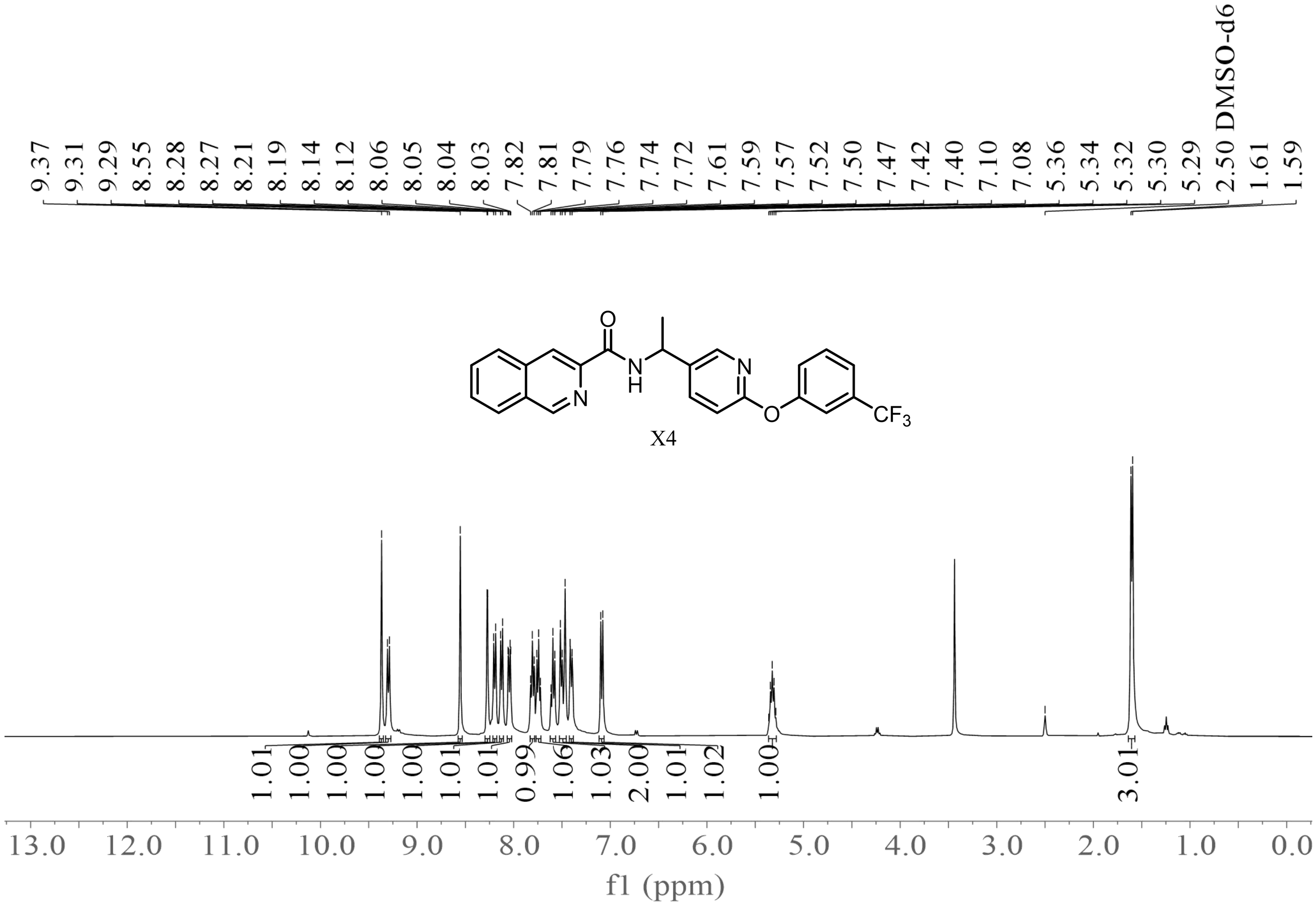

X4

^{1}H NMR spectrum of the compound X4

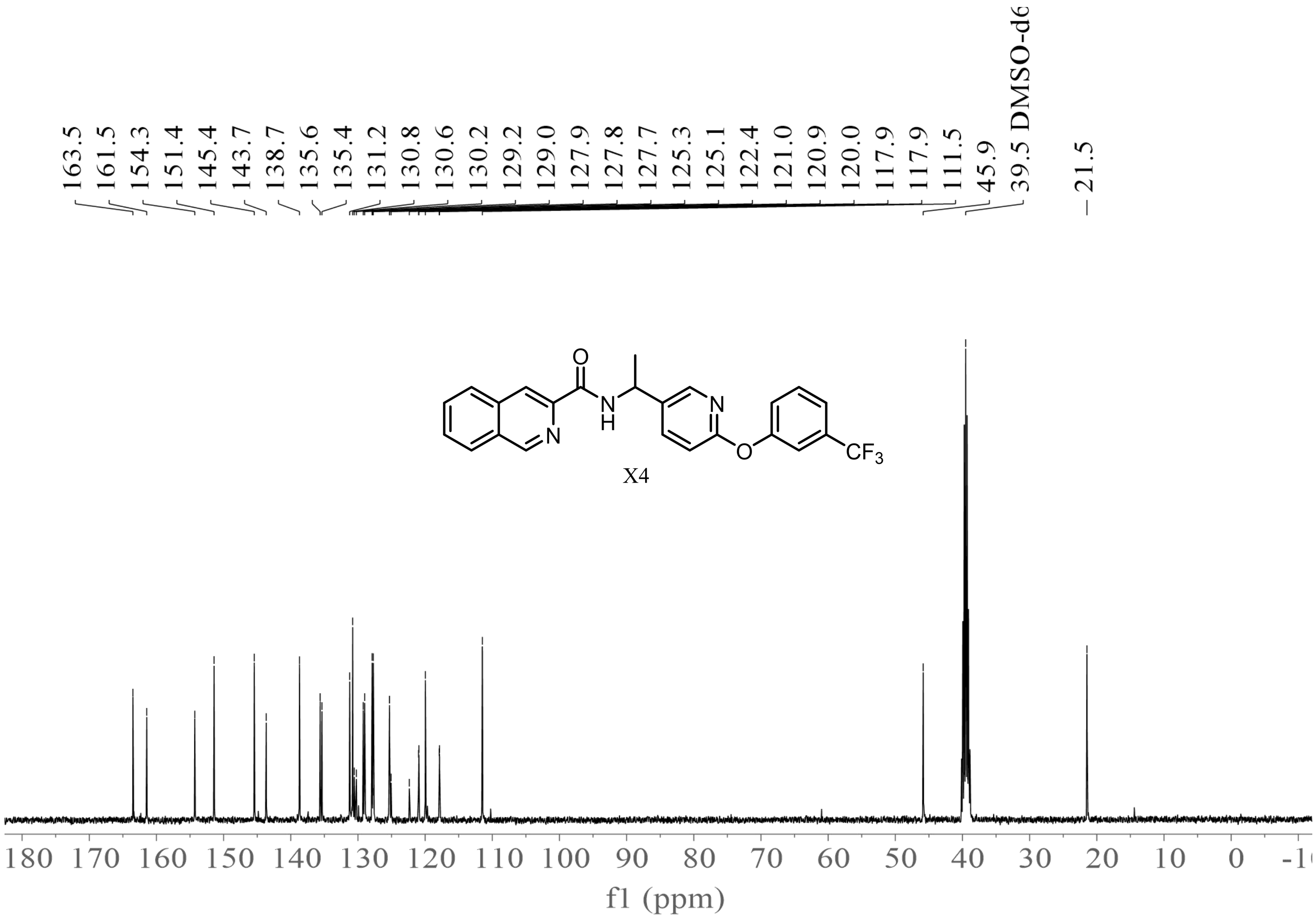

^{13}C NMR spectrum of the compound X4

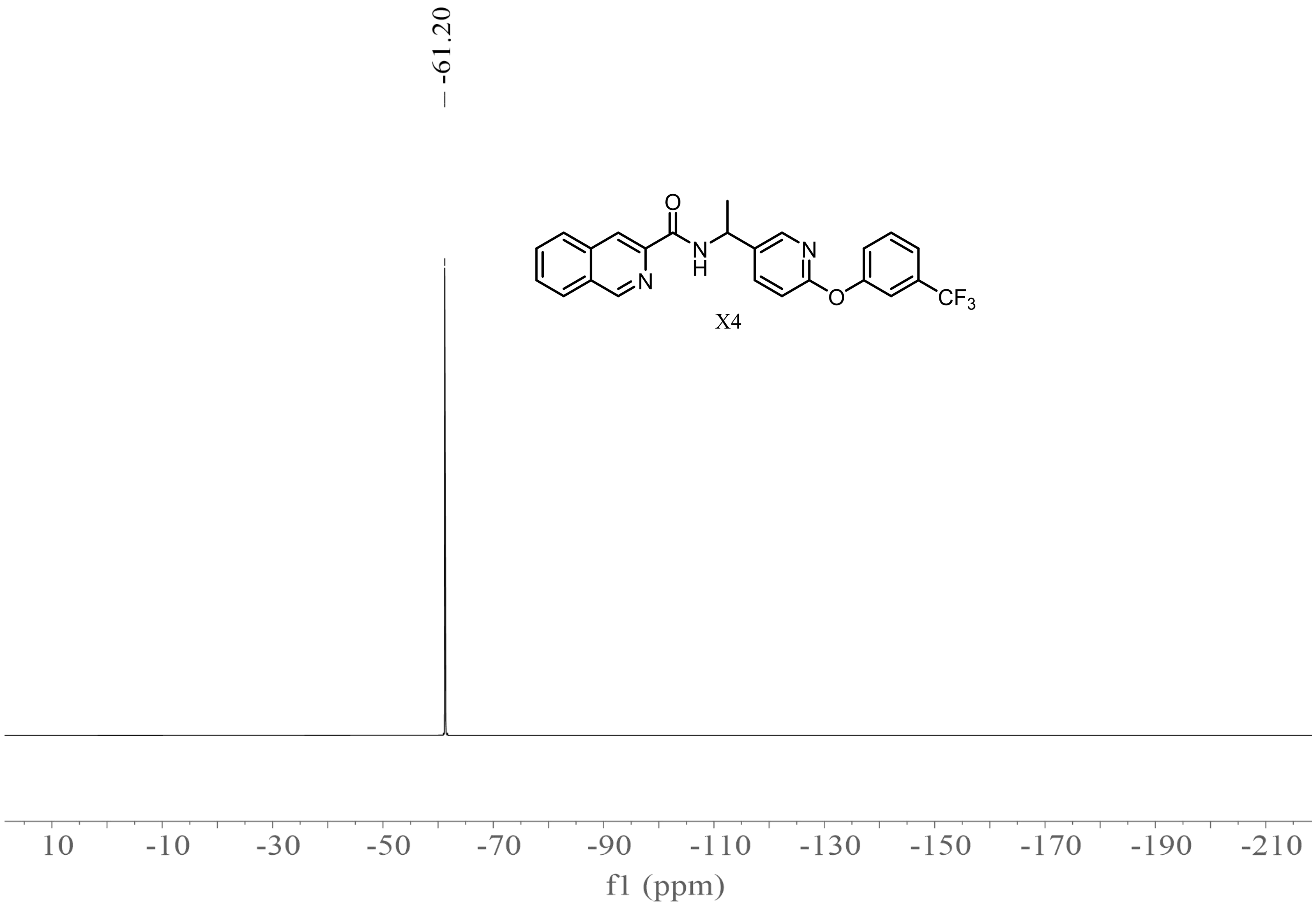

^{19}F NMR spectrum of the compound X4

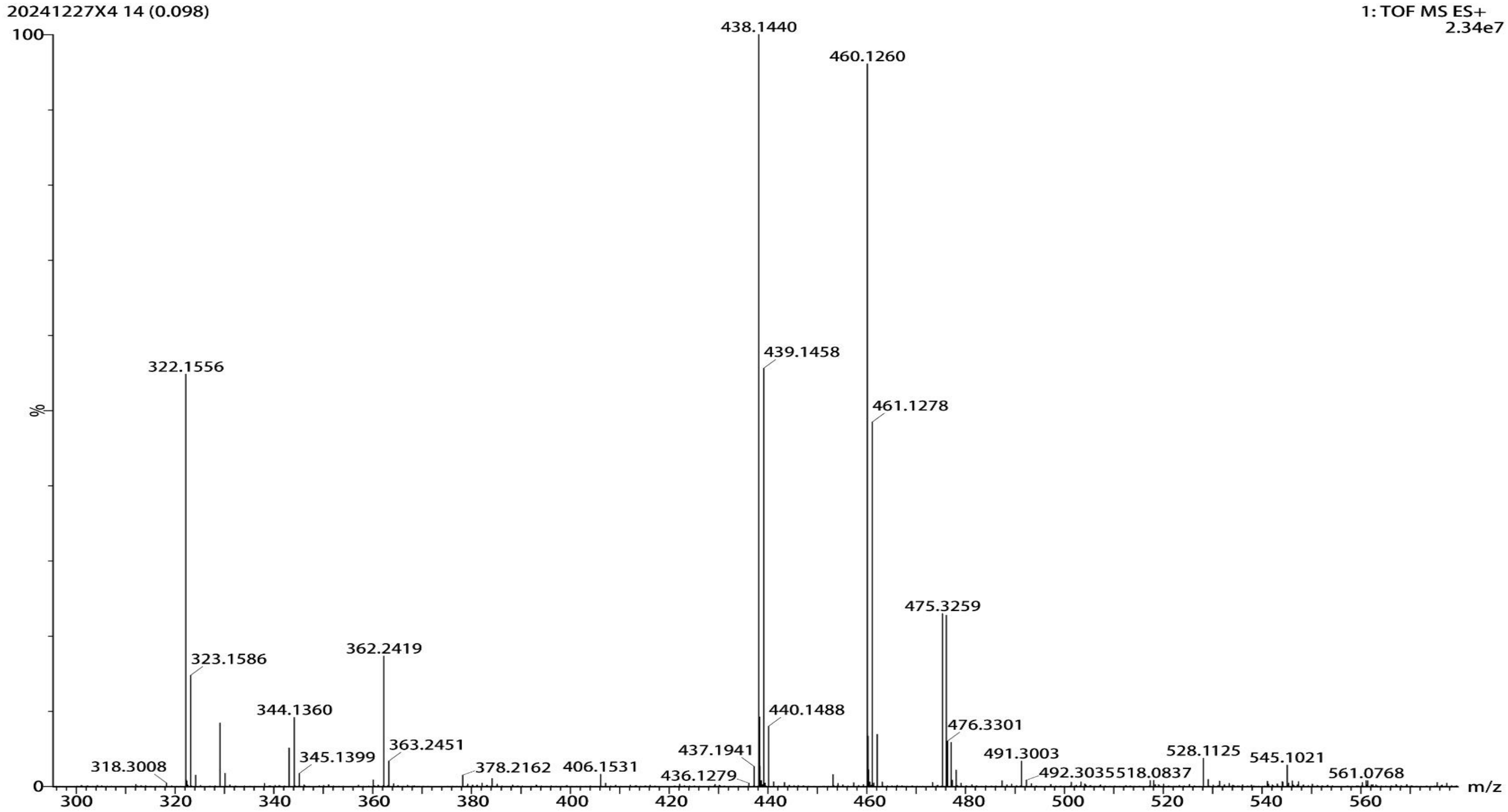

ESI-MS spectrum of the compound X4

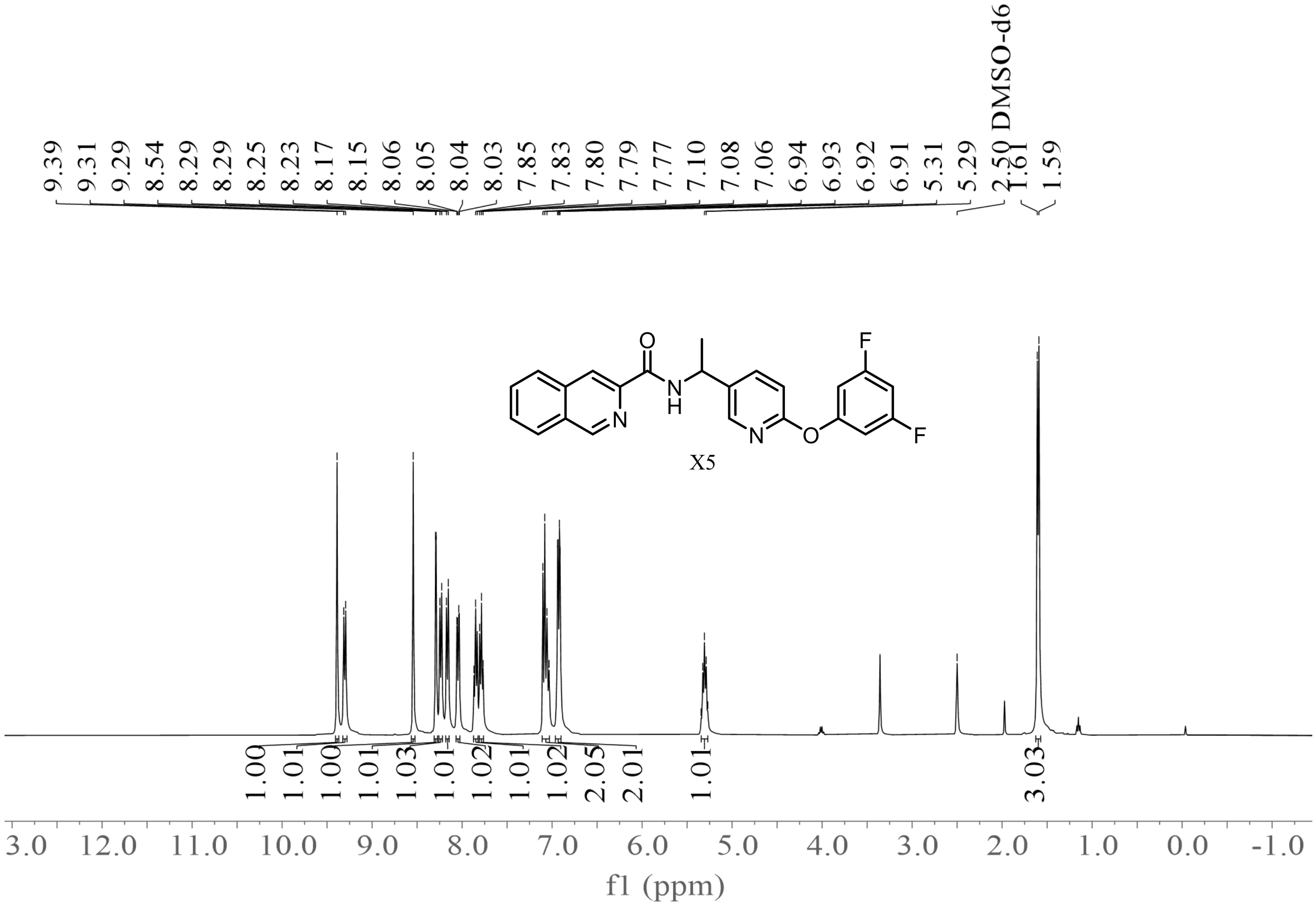

^{1}H NMR spectrum of the compound X5

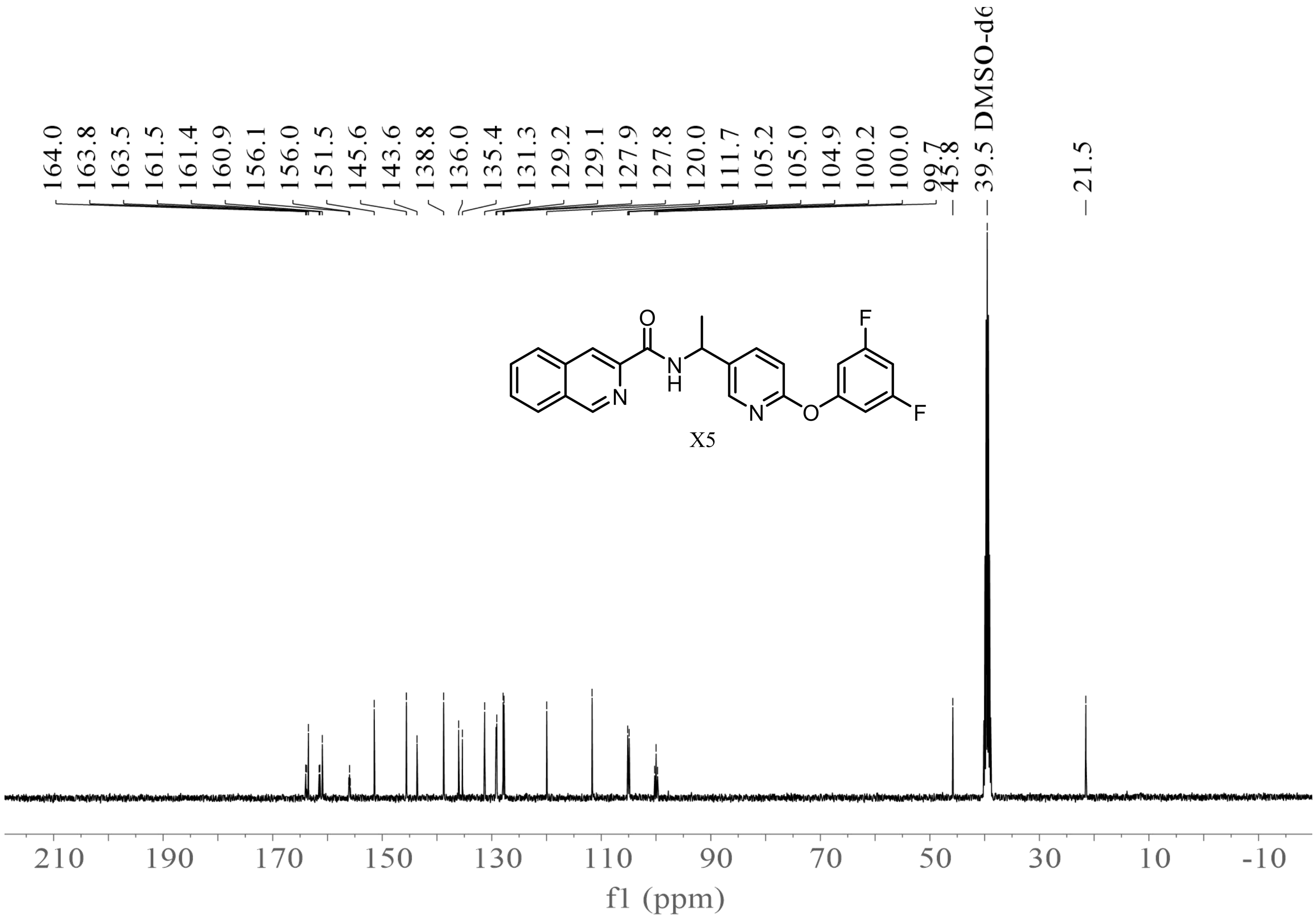

^{13}C NMR spectrum of the compound X5

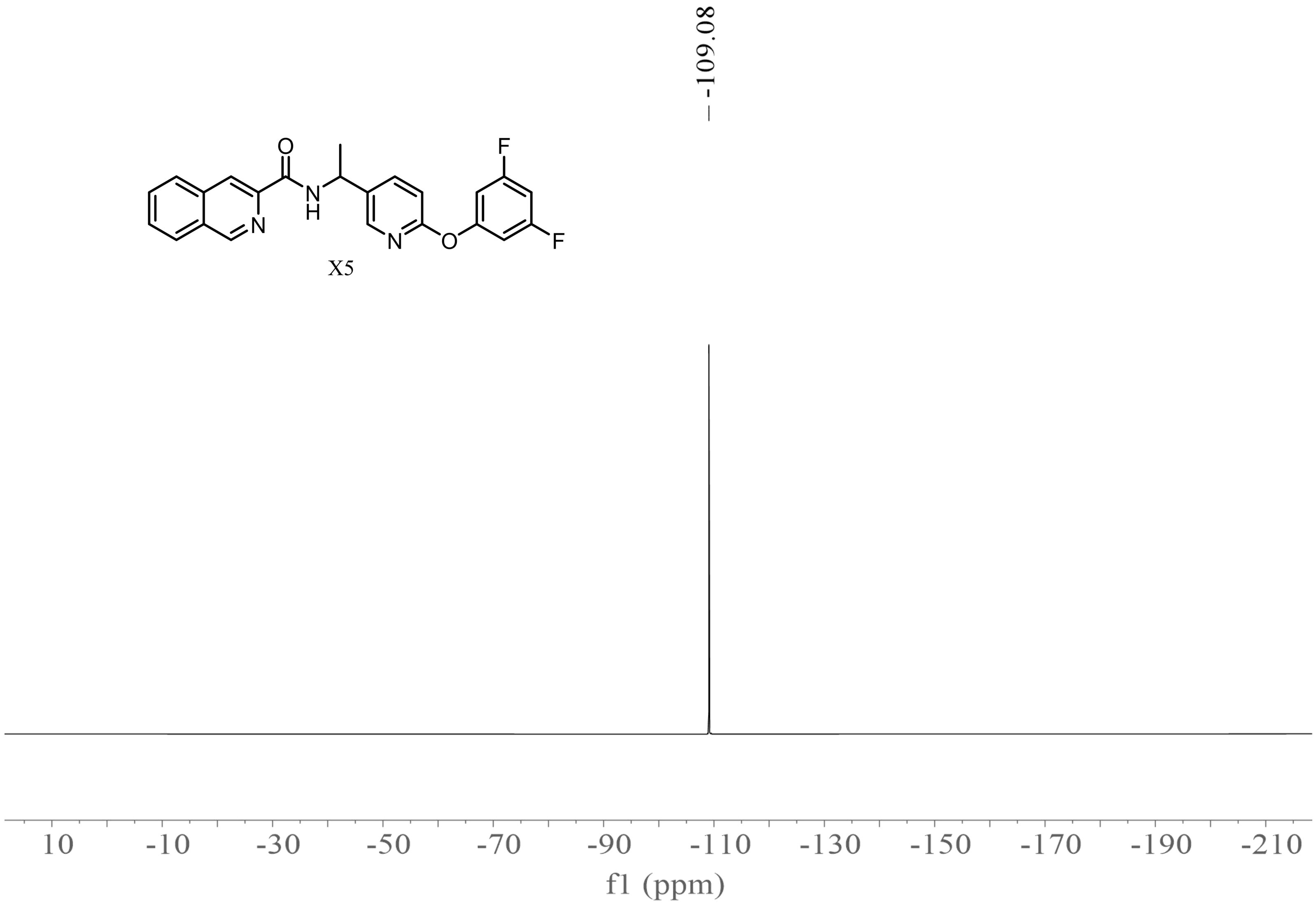

^{19}F NMR spectrum of the compound X5

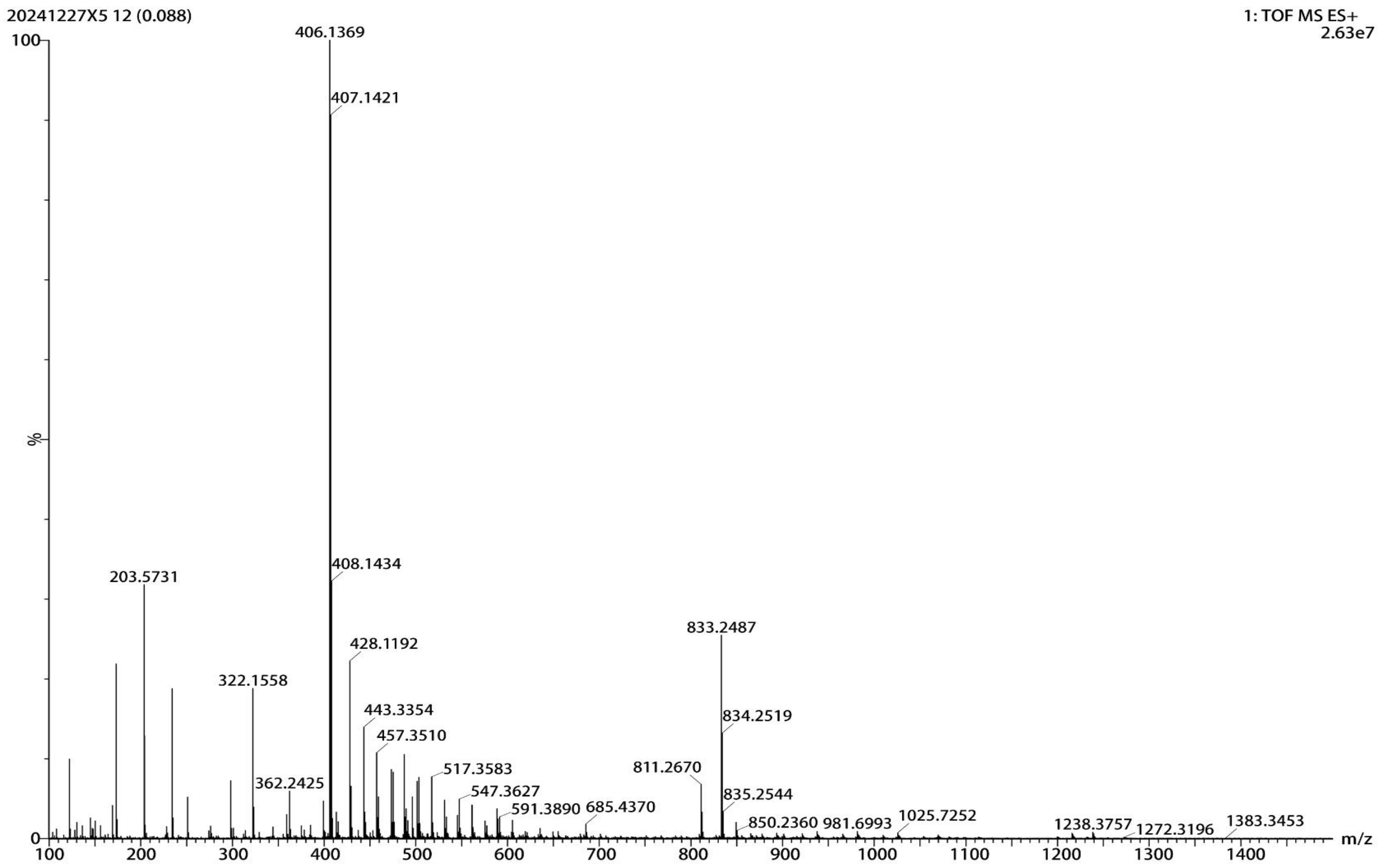

ESI-MS spectrum of the compound X5

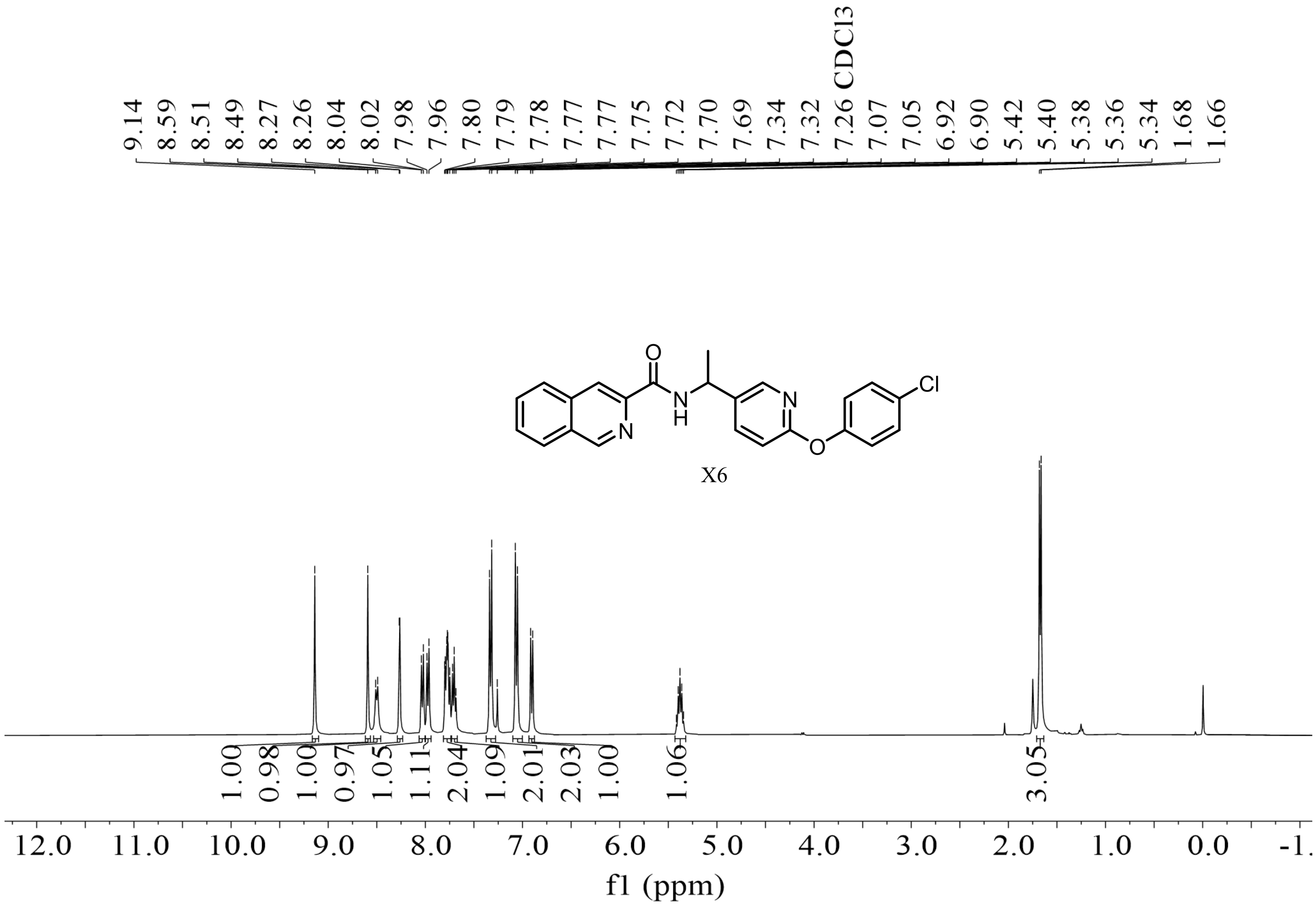

^{1}H NMR spectrum of the compound X6

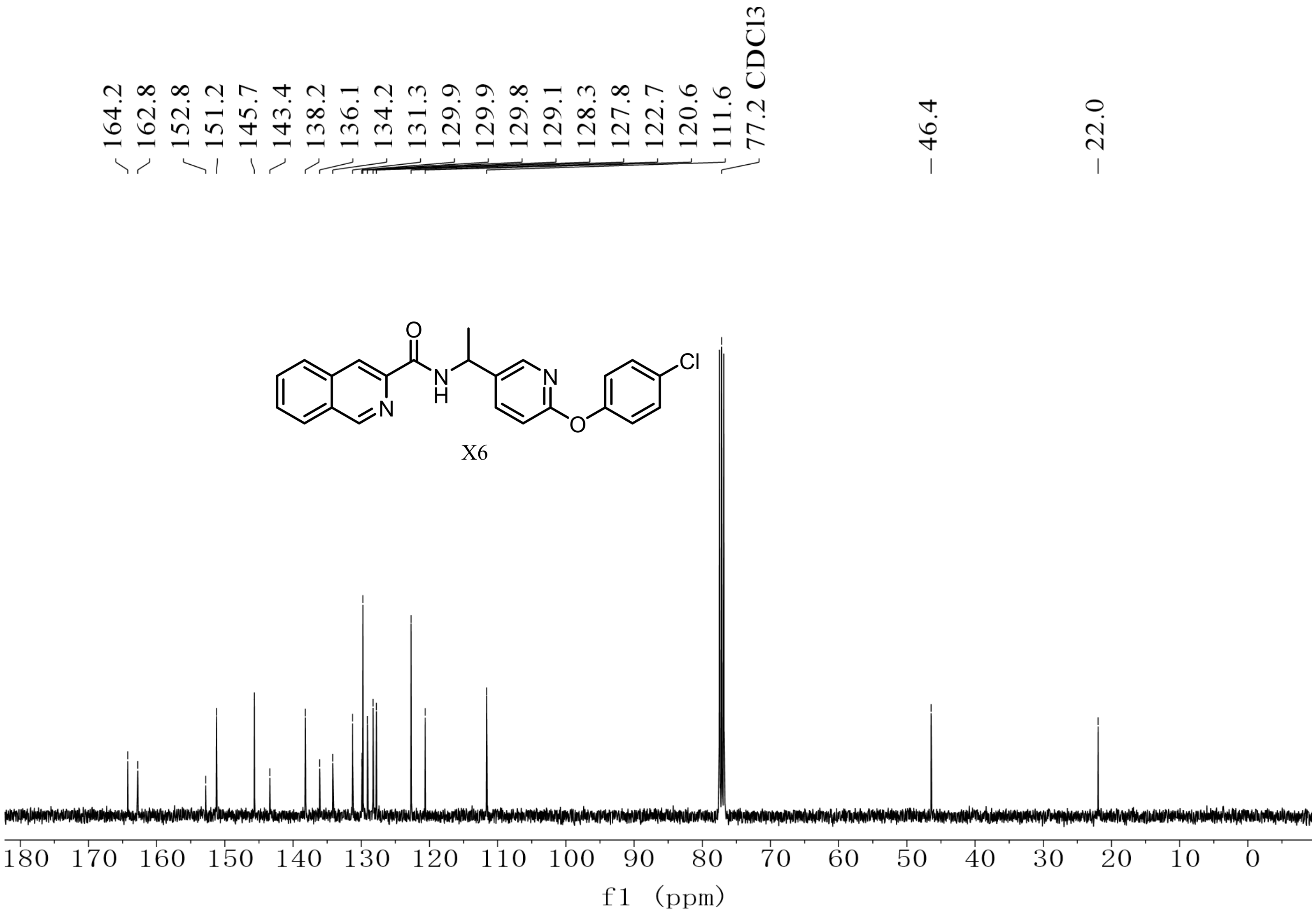

^{13}C NMR spectrum of the compound X6

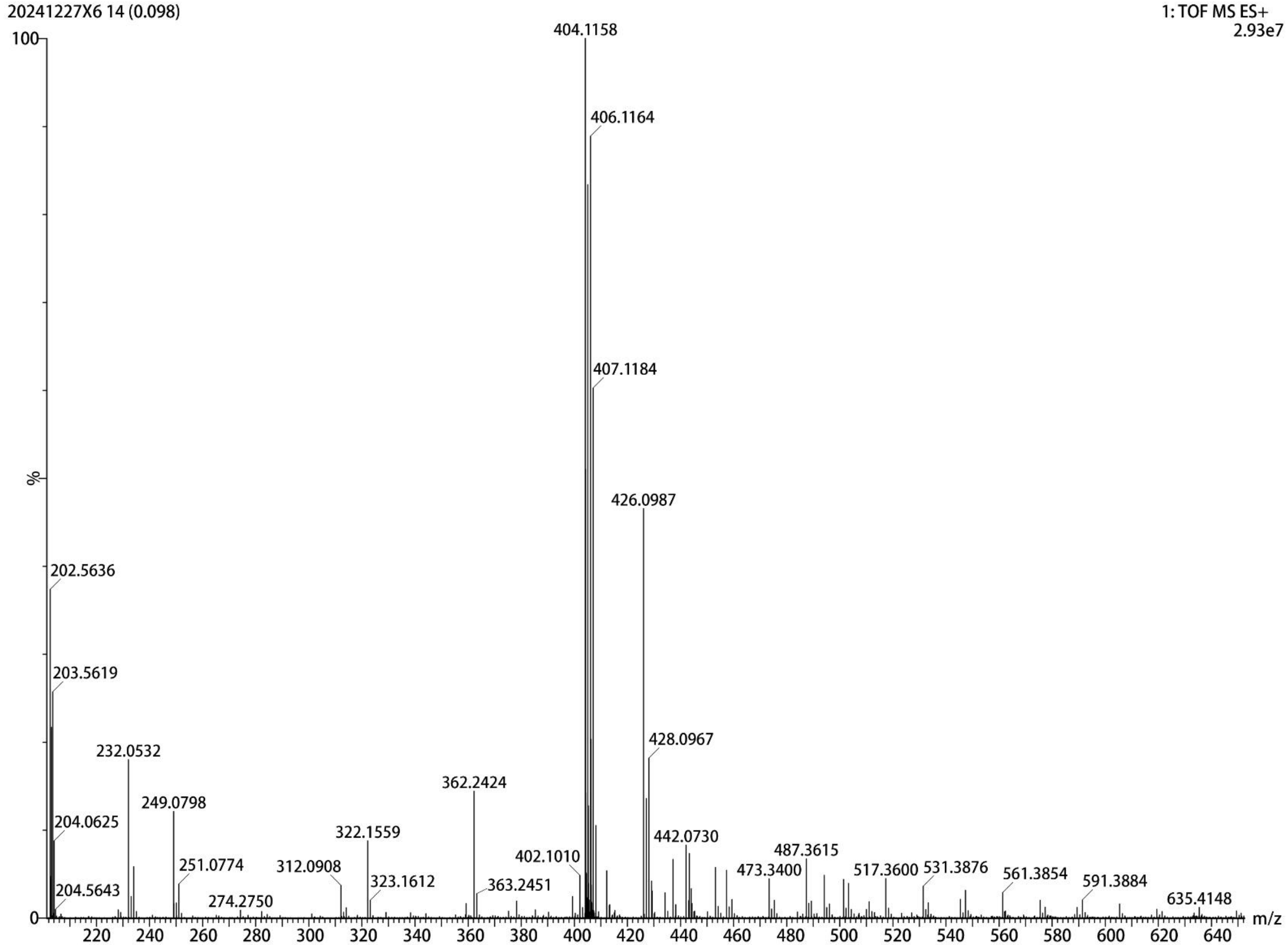

ESI-MS spectrum of the compound X6

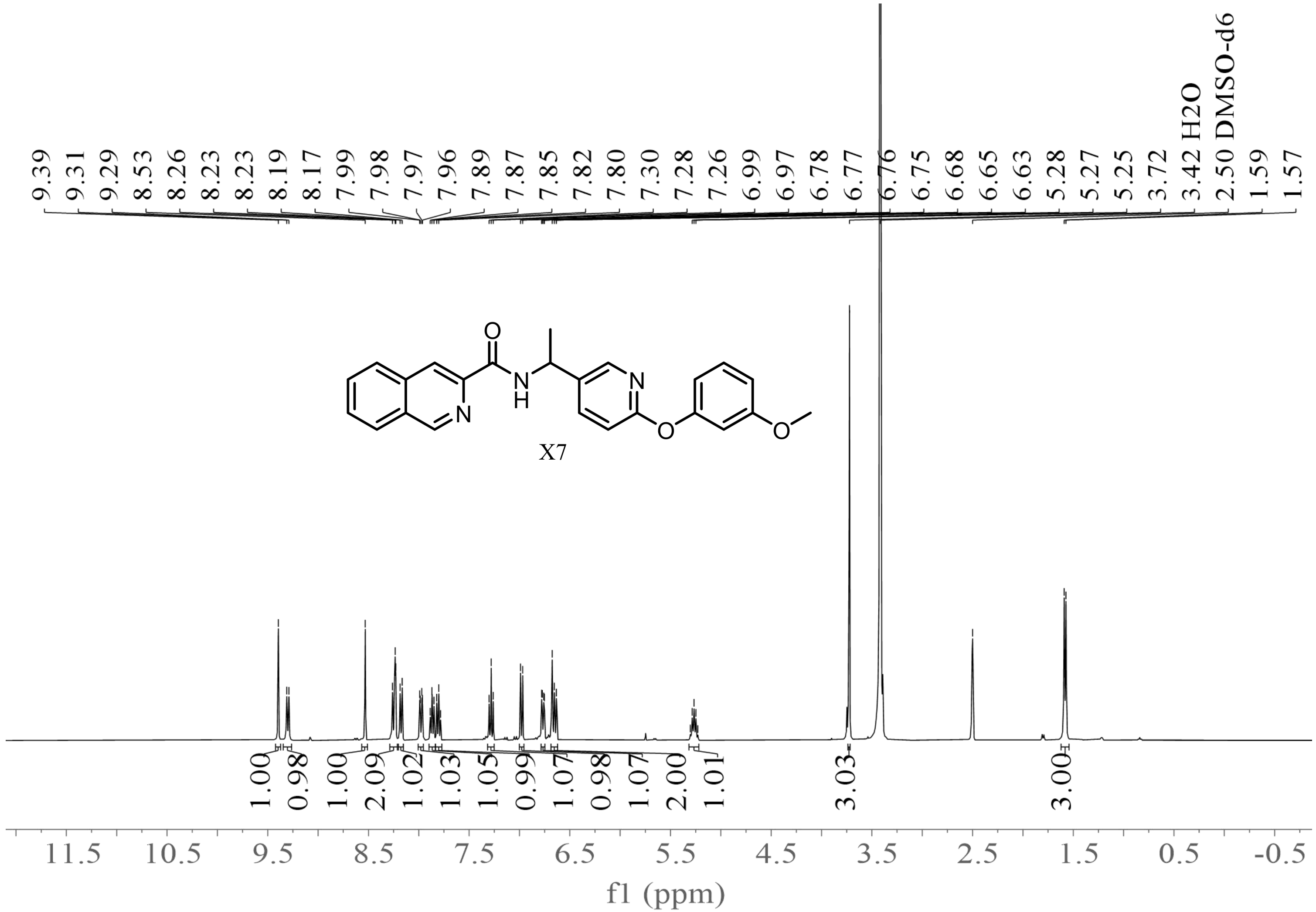

^{1}H NMR spectrum of the compound X7

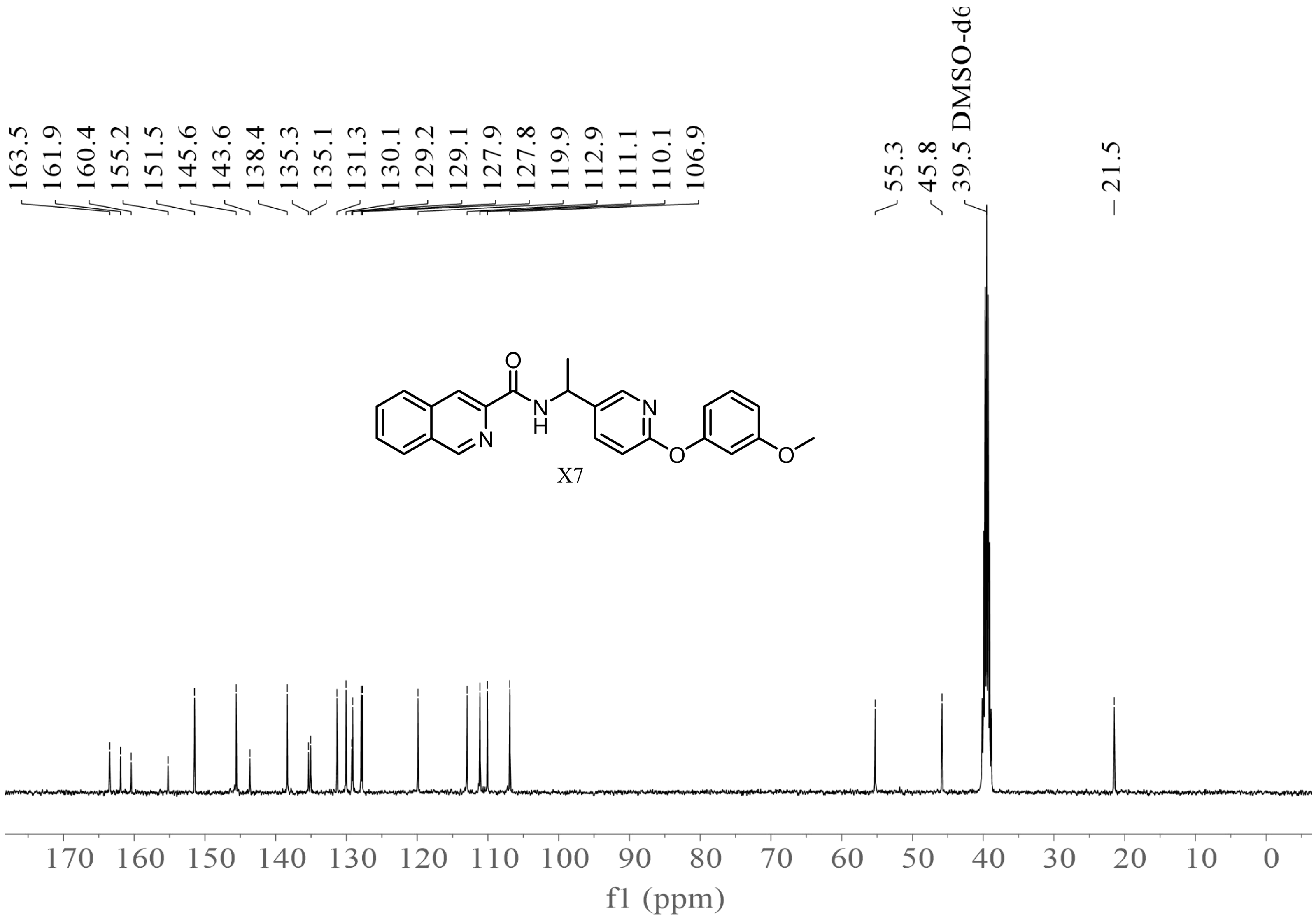

^{13}C NMR spectrum of the compound X7

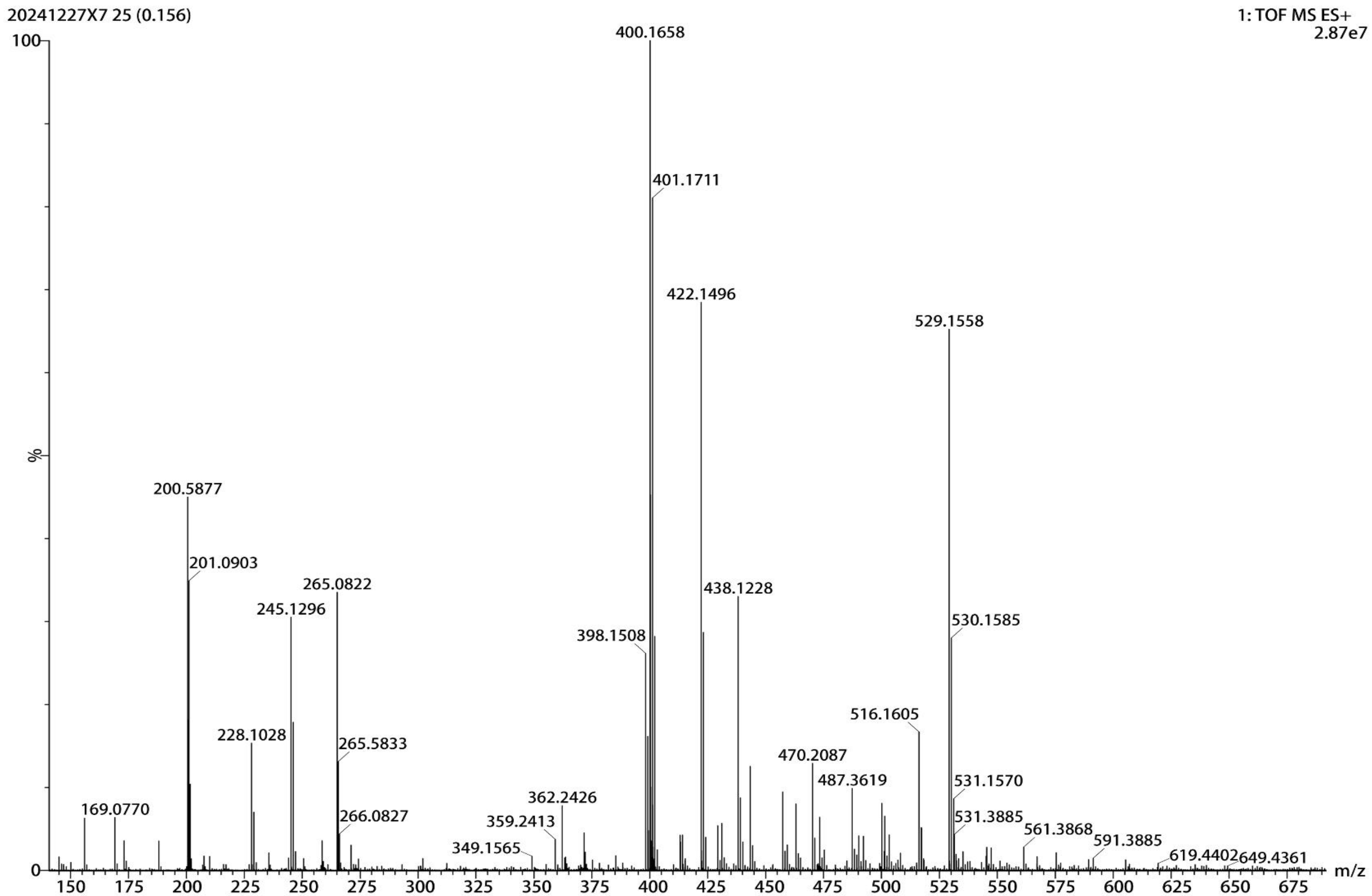

ESI-MS spectrum of the compound X7

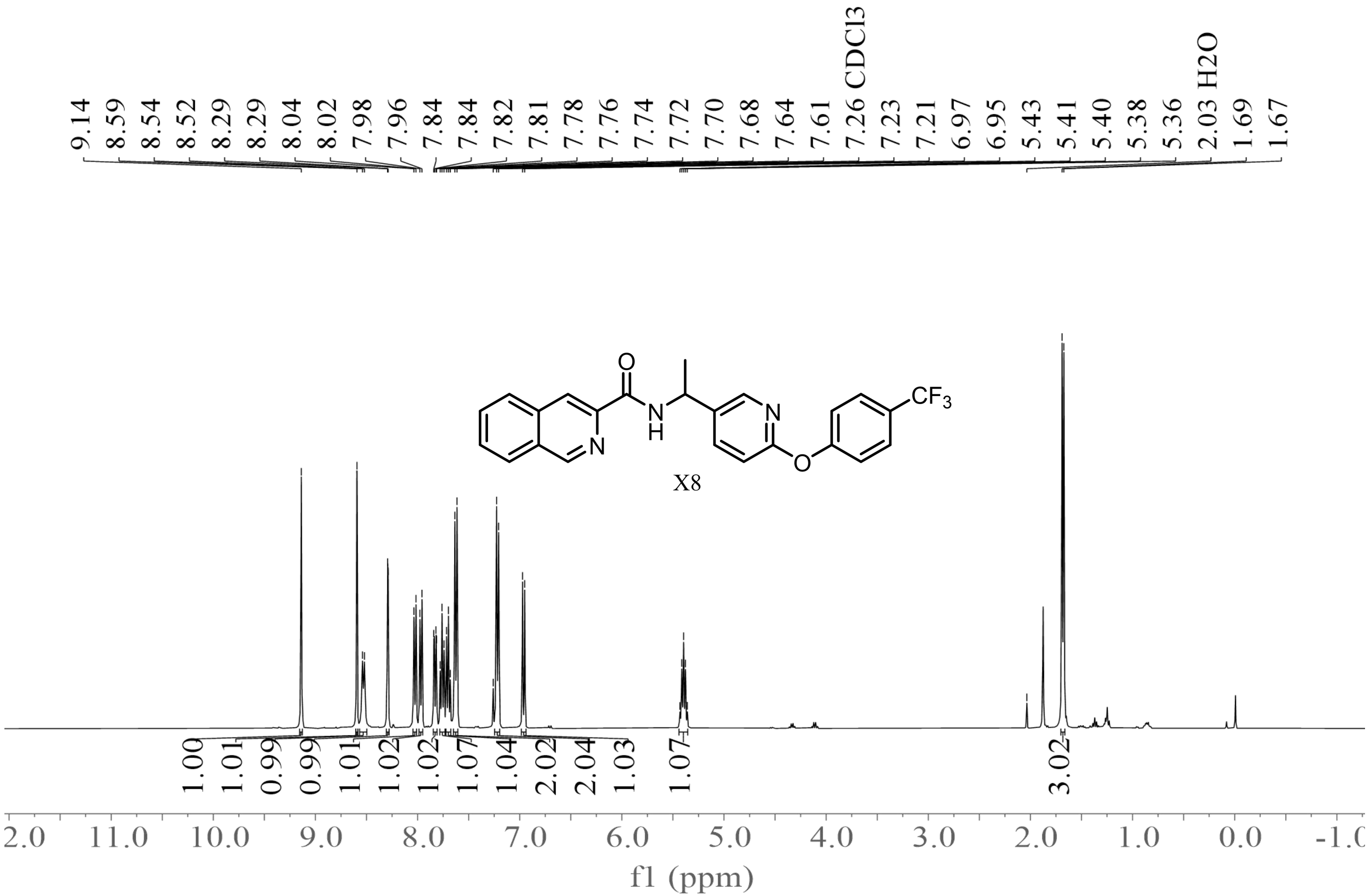

^{1}H NMR spectrum of the compound X8

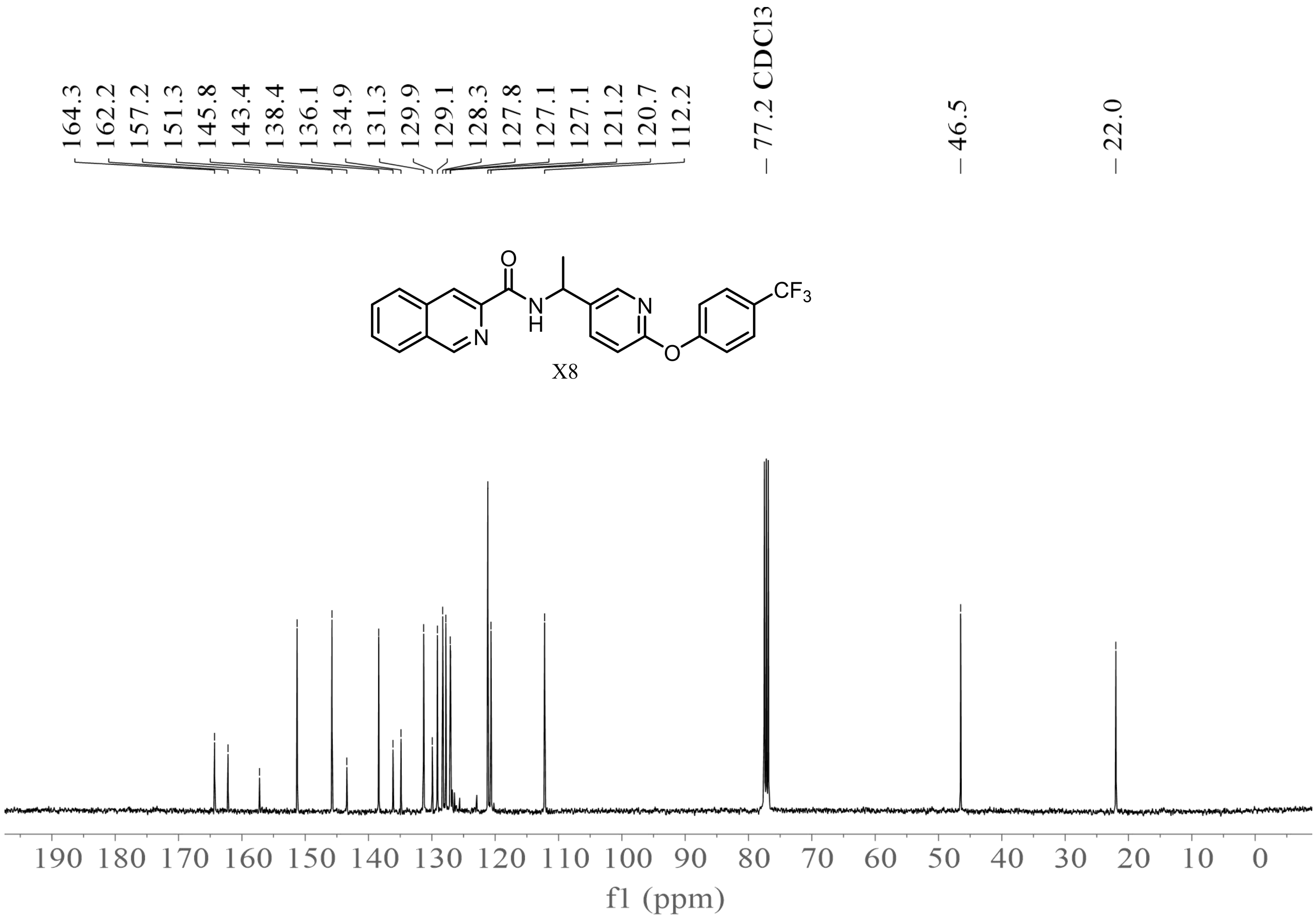

^{13}C NMR spectrum of the compound X8

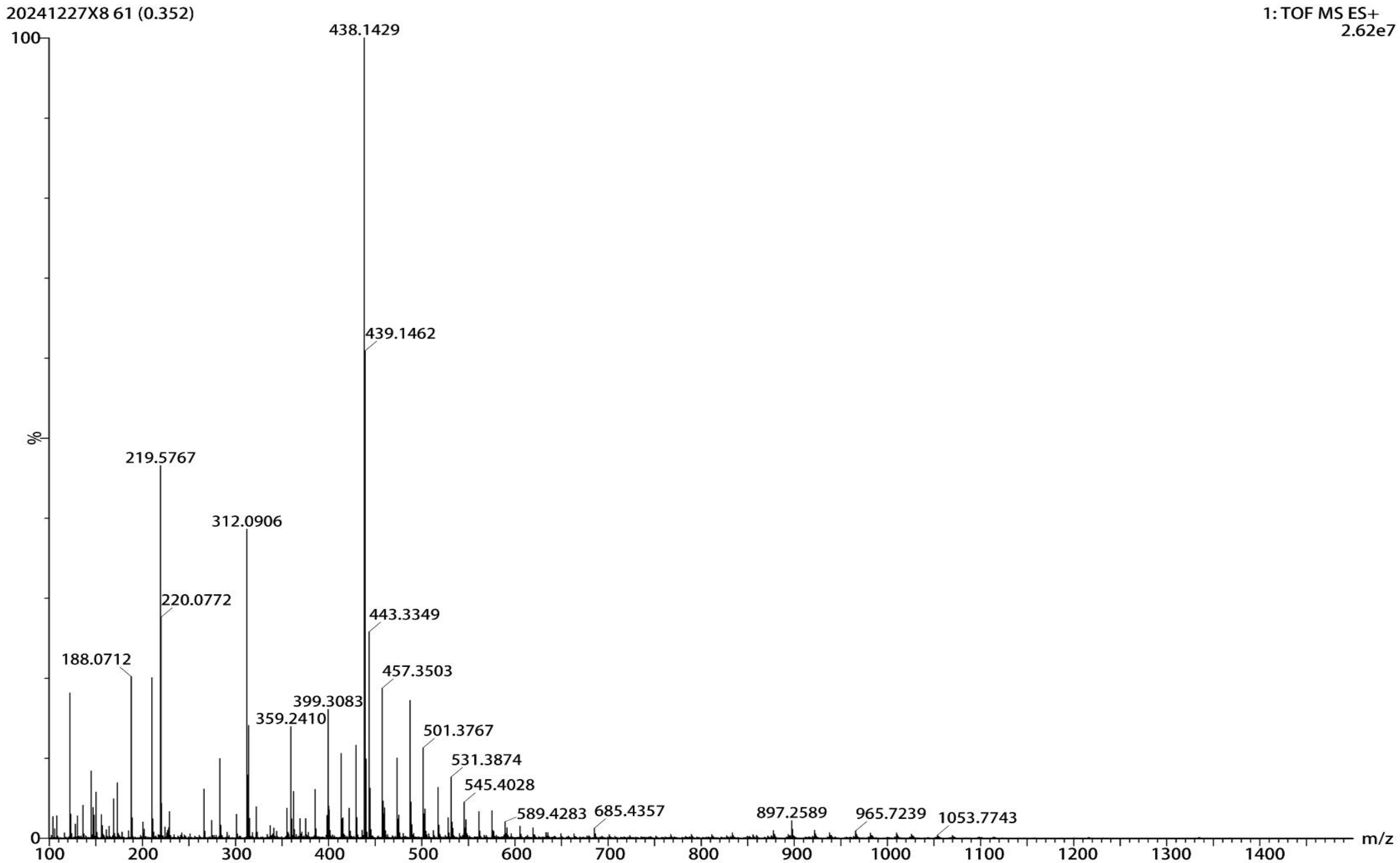

ESI-MS spectrum of the compound X8

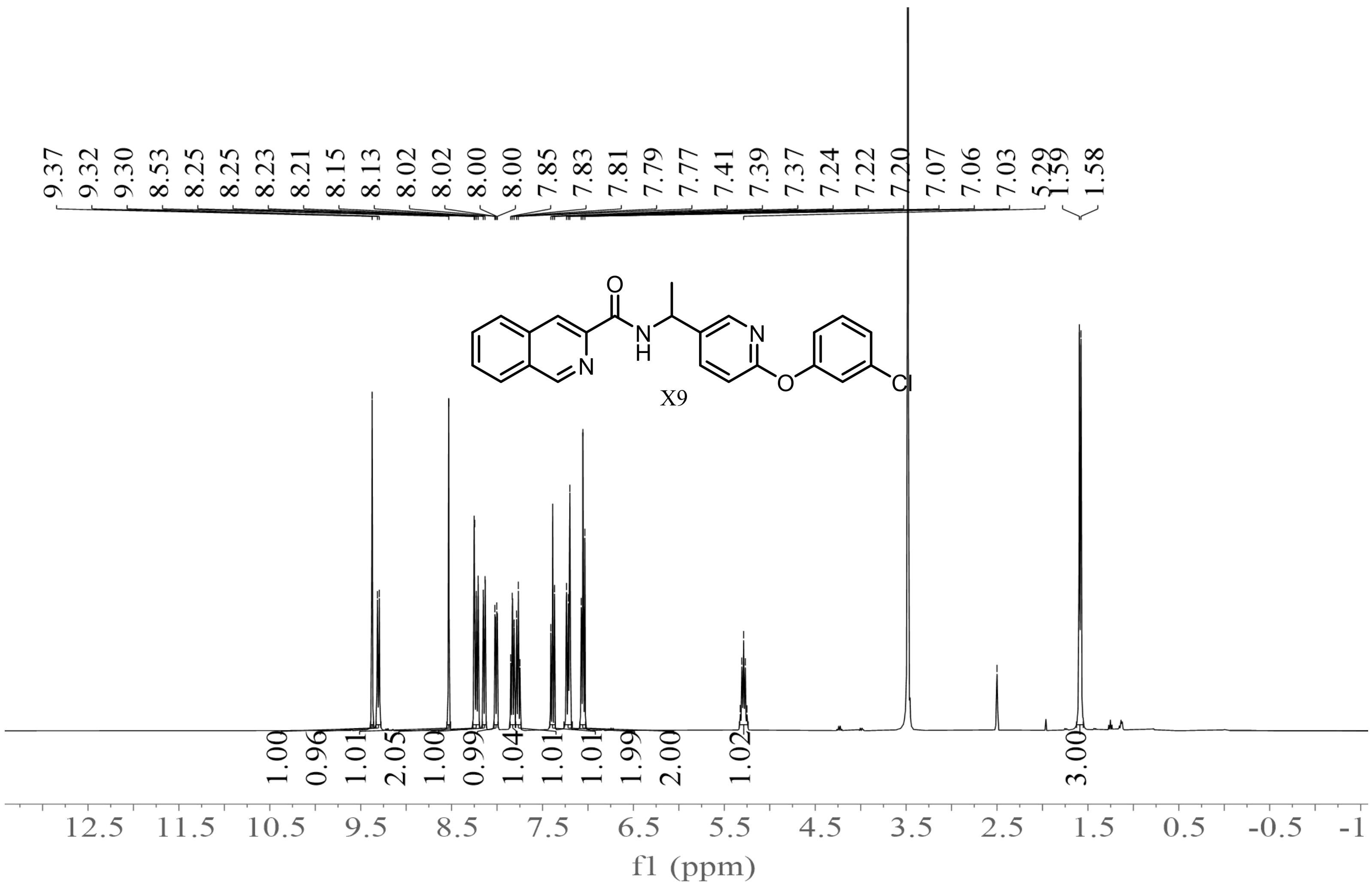

^{1}H NMR spectrum of the compound X9

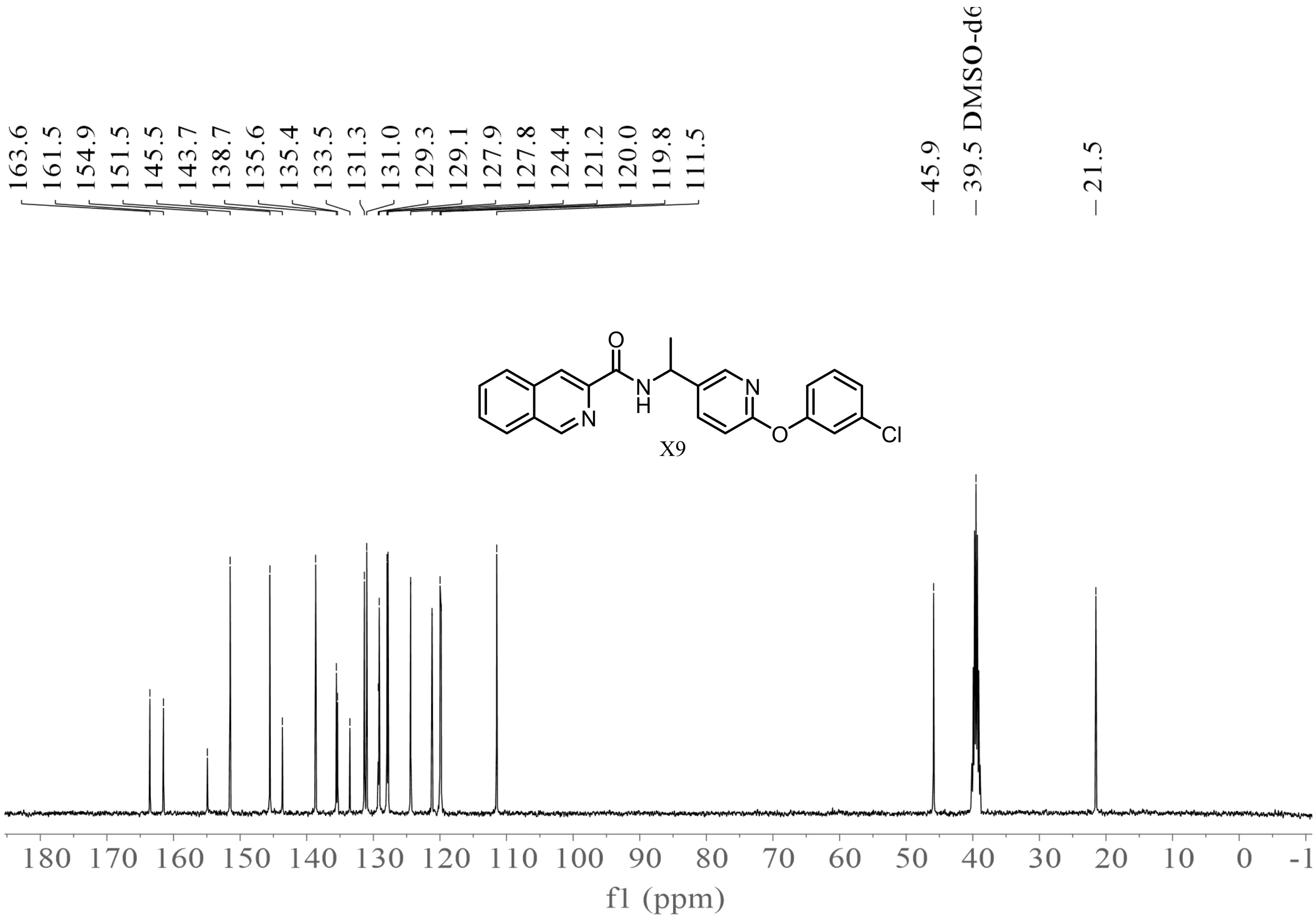

^{13}C NMR spectrum of the compound X9

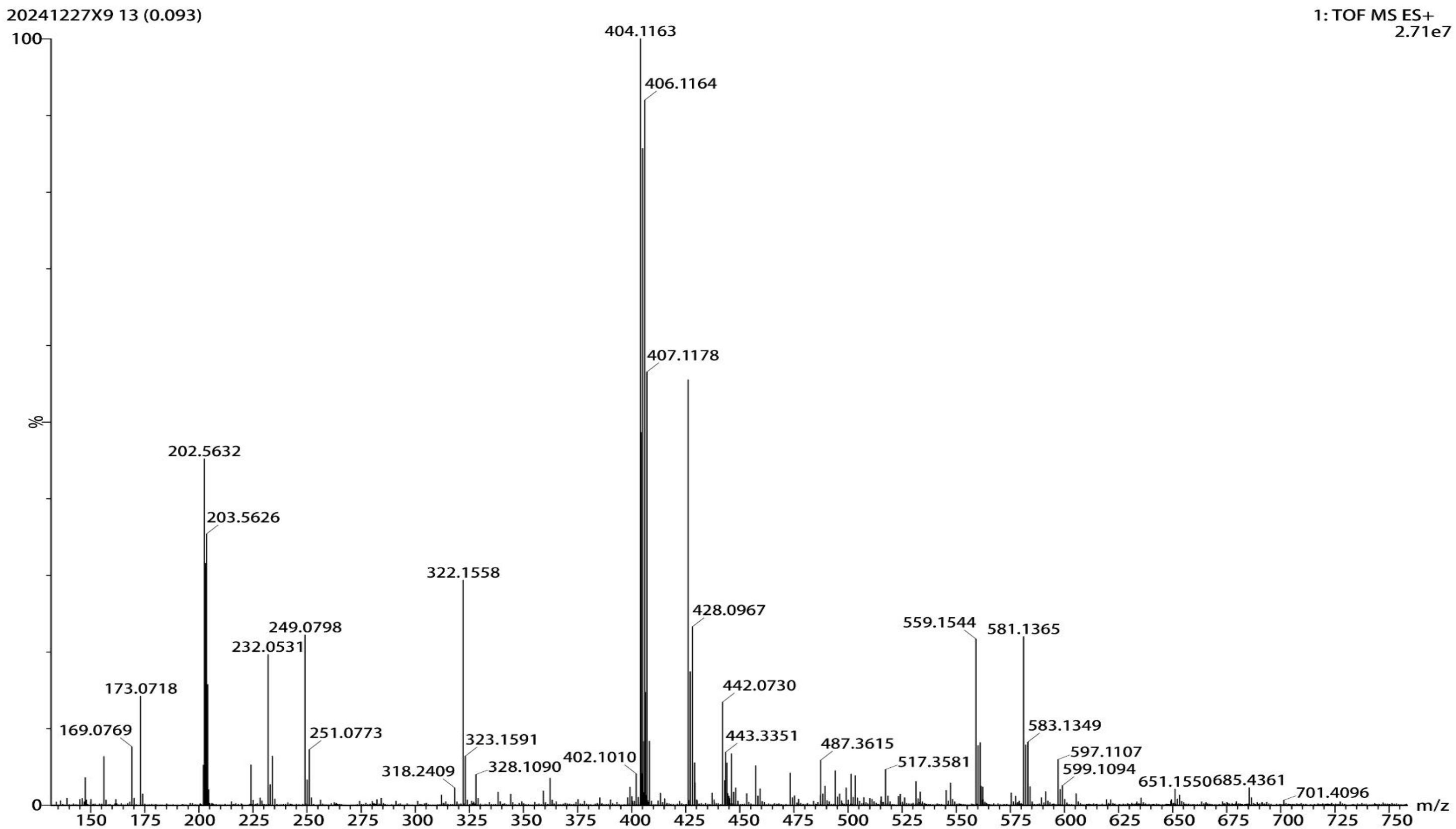

ESI-MS spectrum of the compound X9

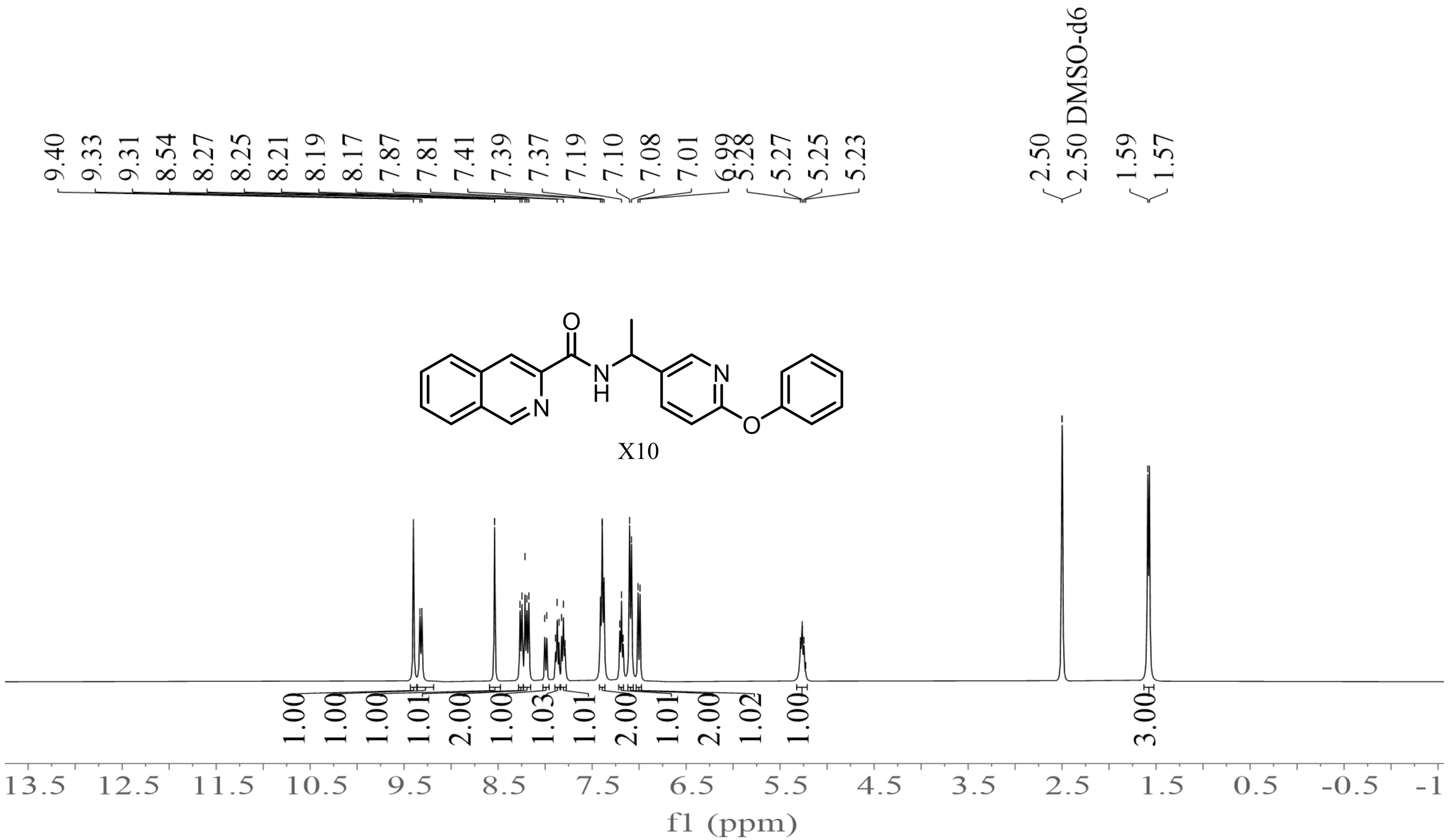

^{1}H NMR spectrum of the compound X10

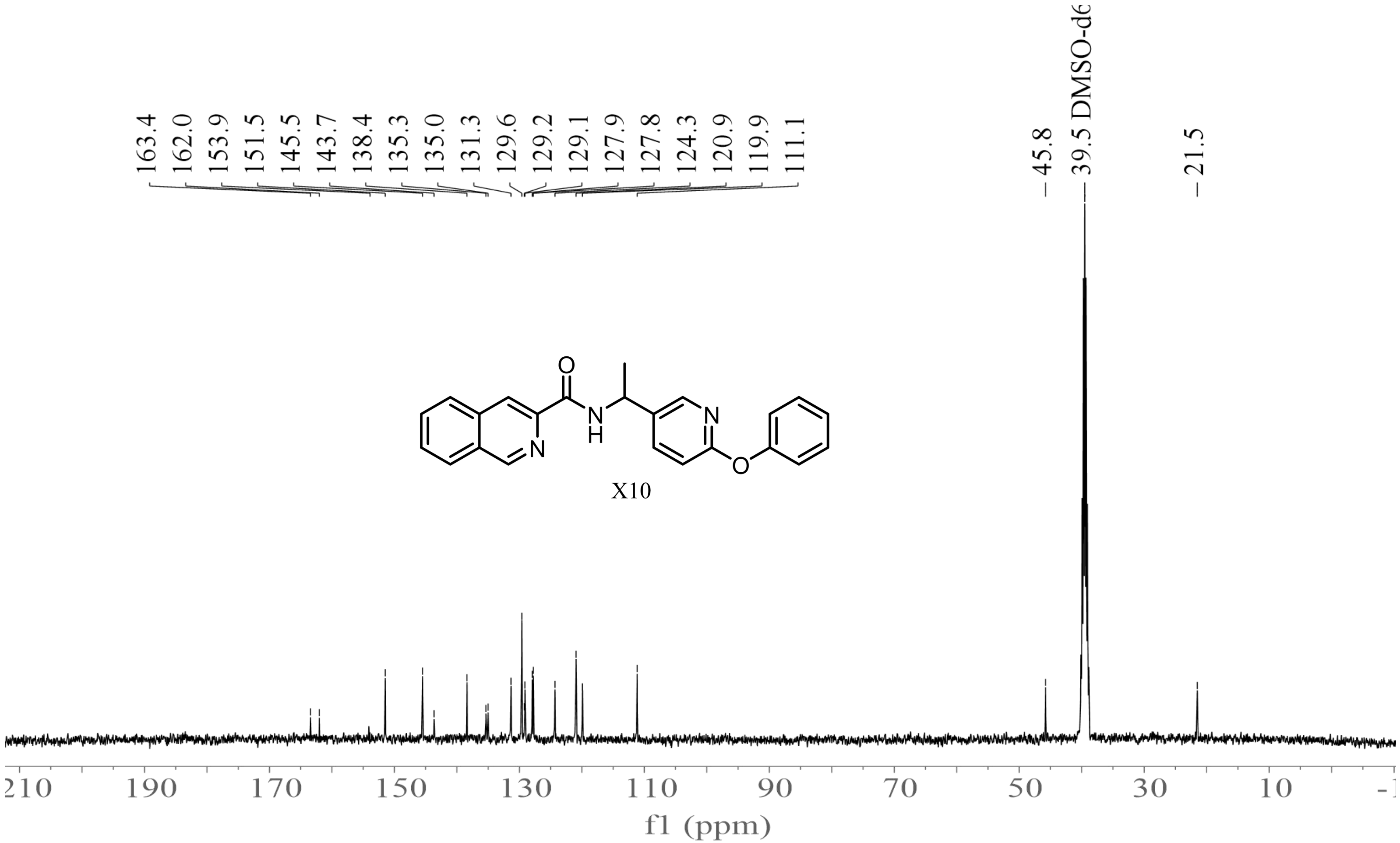

^{13}C NMR spectrum of the compound X10

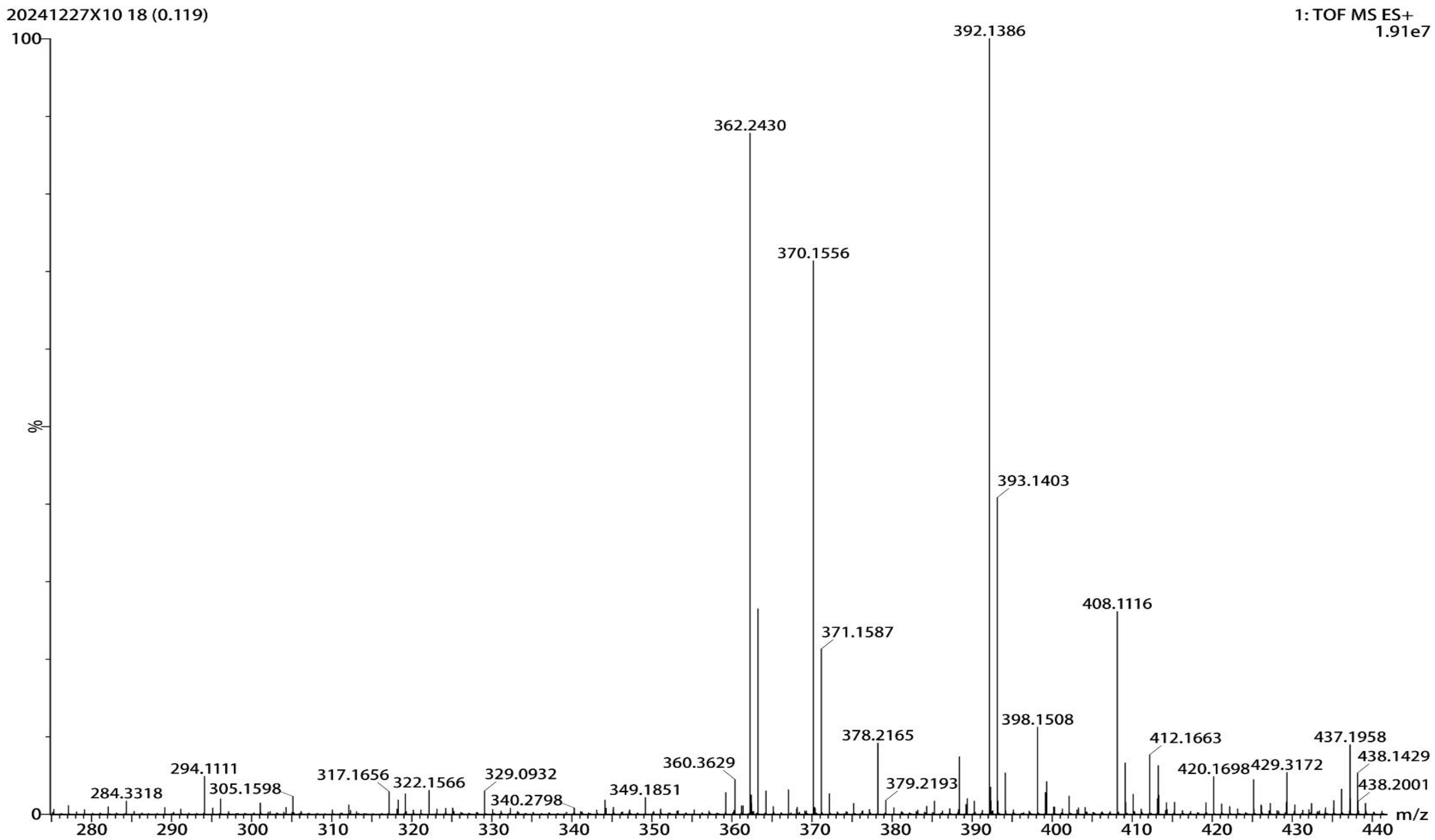

ESI-MS spectrum of the compound X10

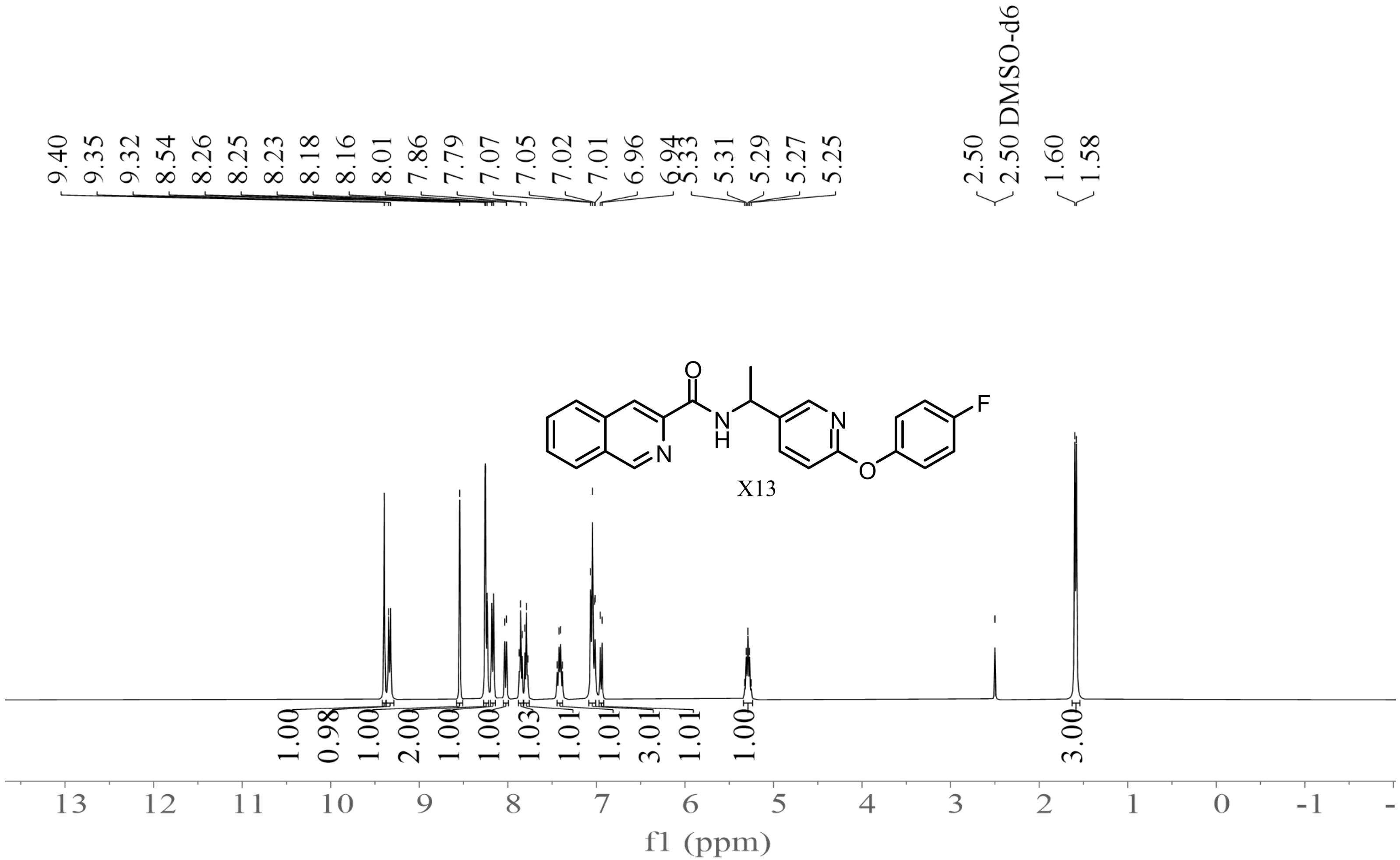

¹H NMR spectrum of the compound X13

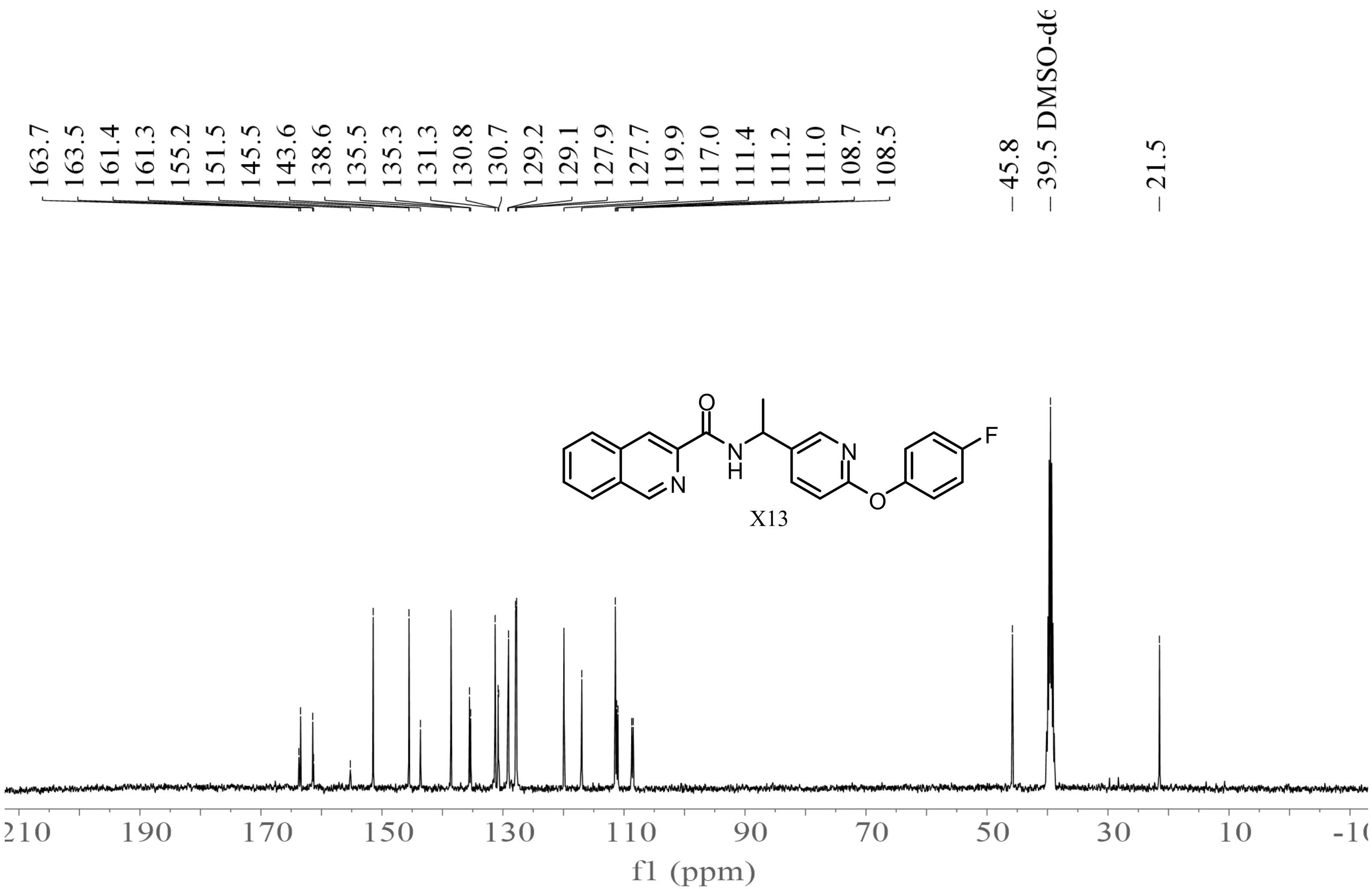

^{13}C NMR spectrum of the compound X13

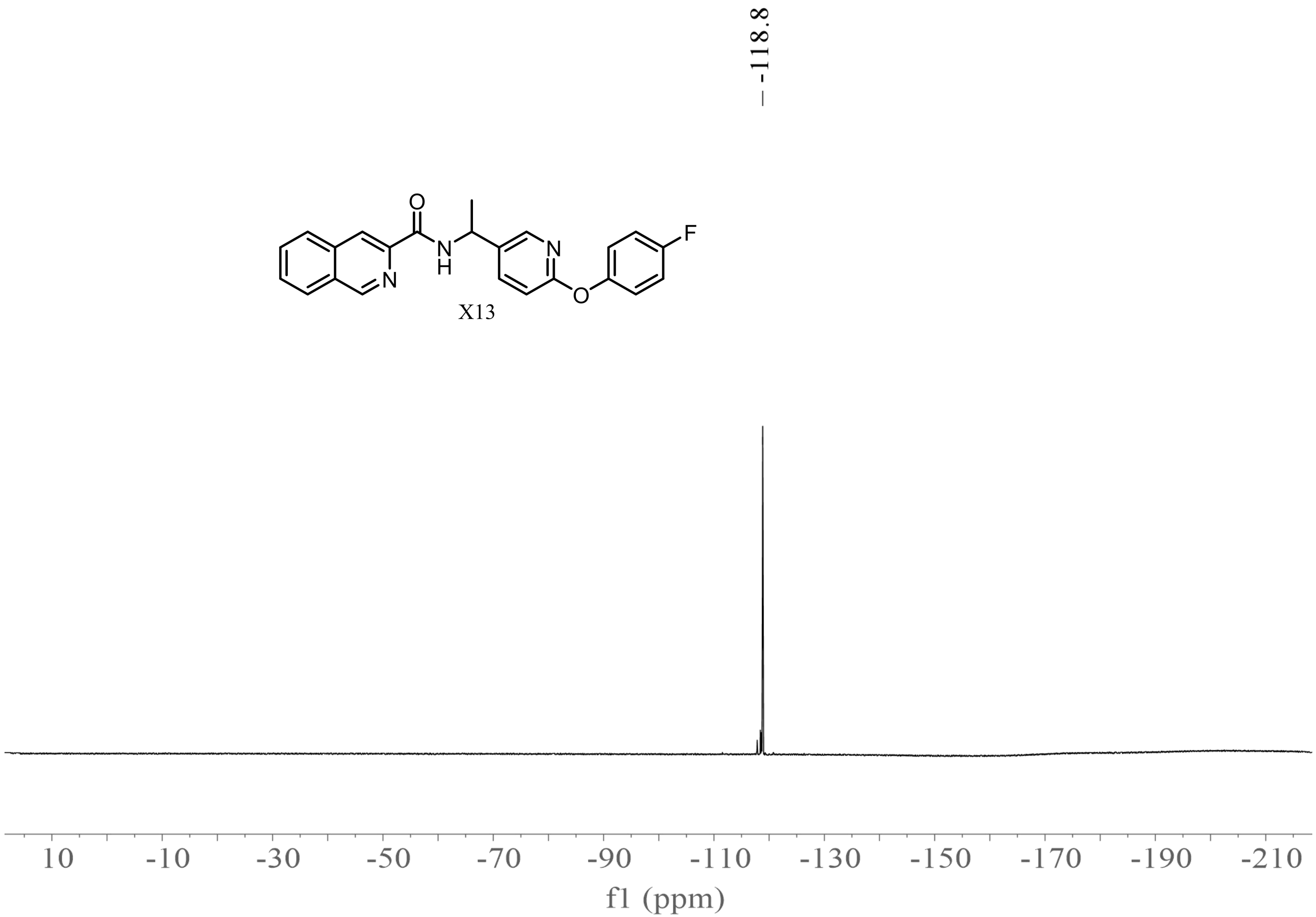

^{19}F NMR spectrum of the compound X13

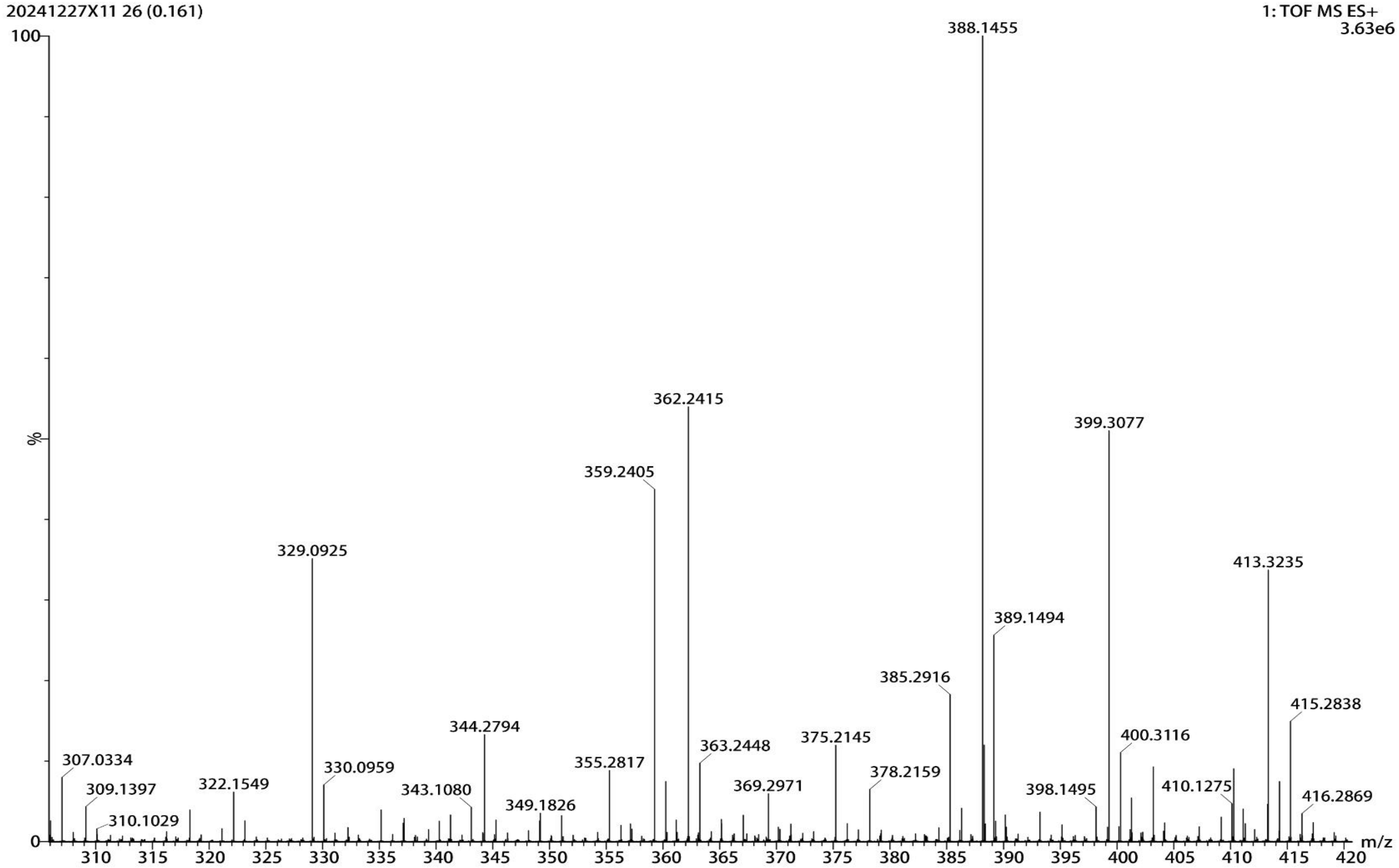

ESI-MS spectrum of the compound X13

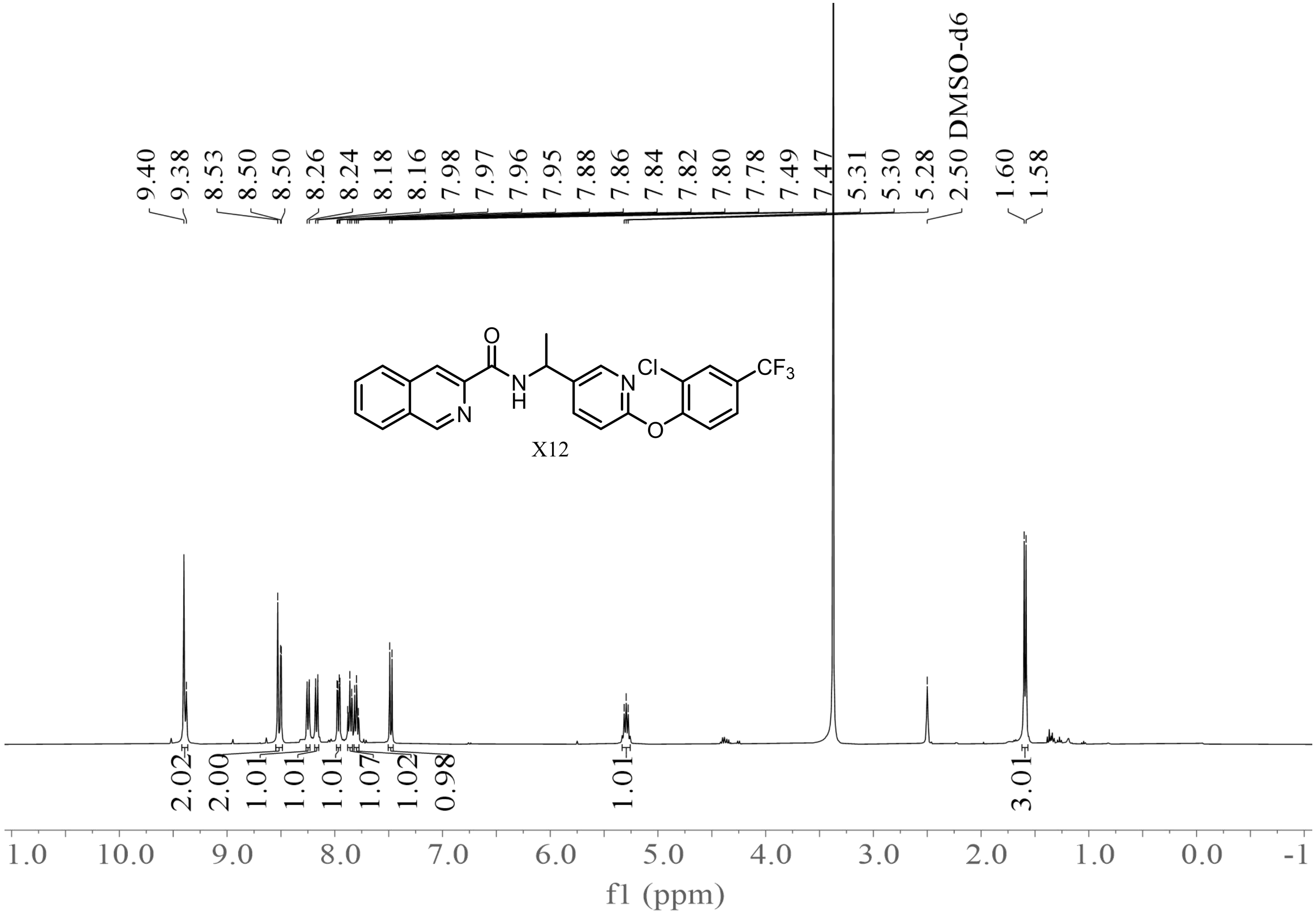

^{1}H NMR spectrum of the compound X12

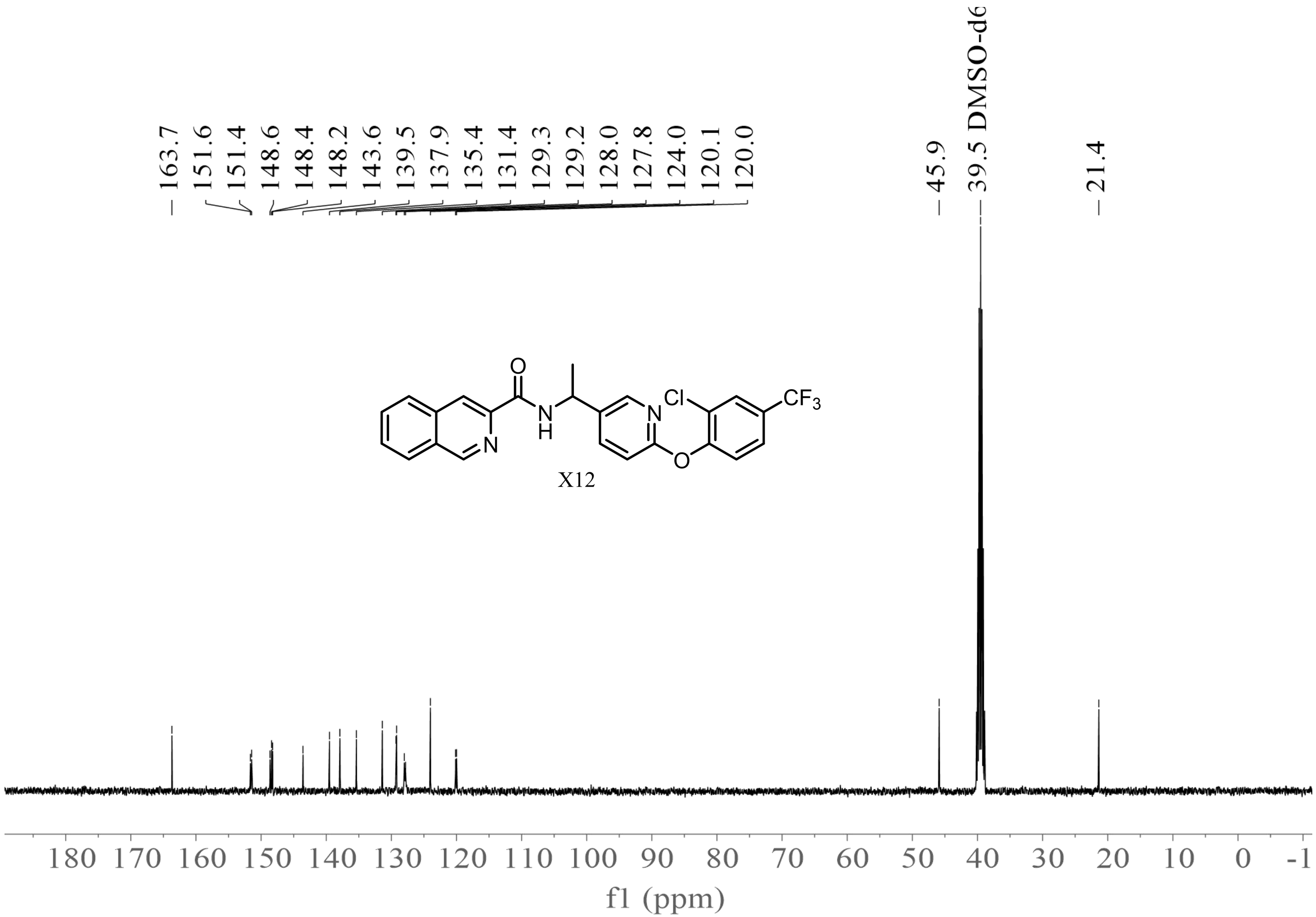

^{13}C NMR spectrum of the compound X12

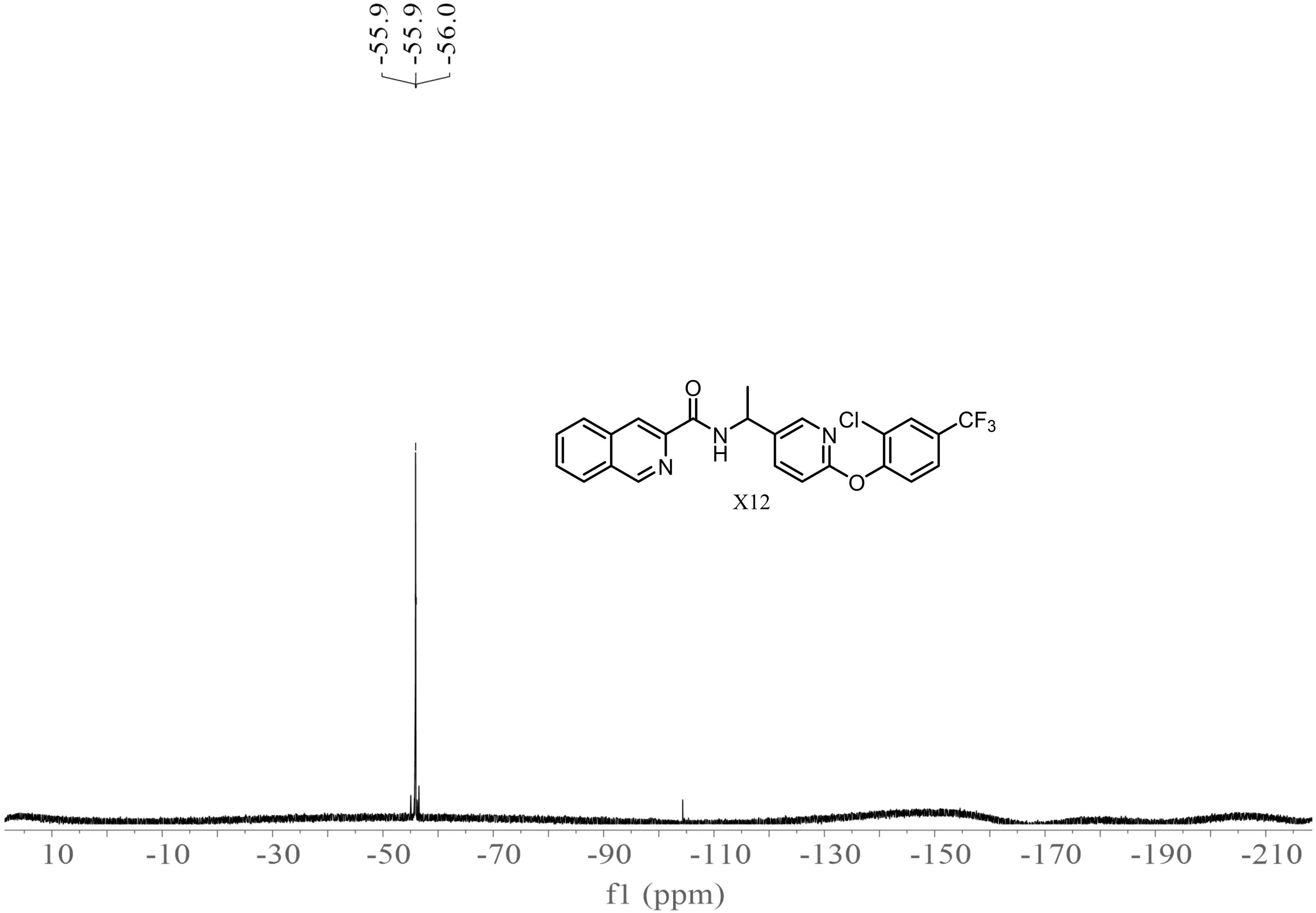

^{19}F NMR spectrum of the compound X12

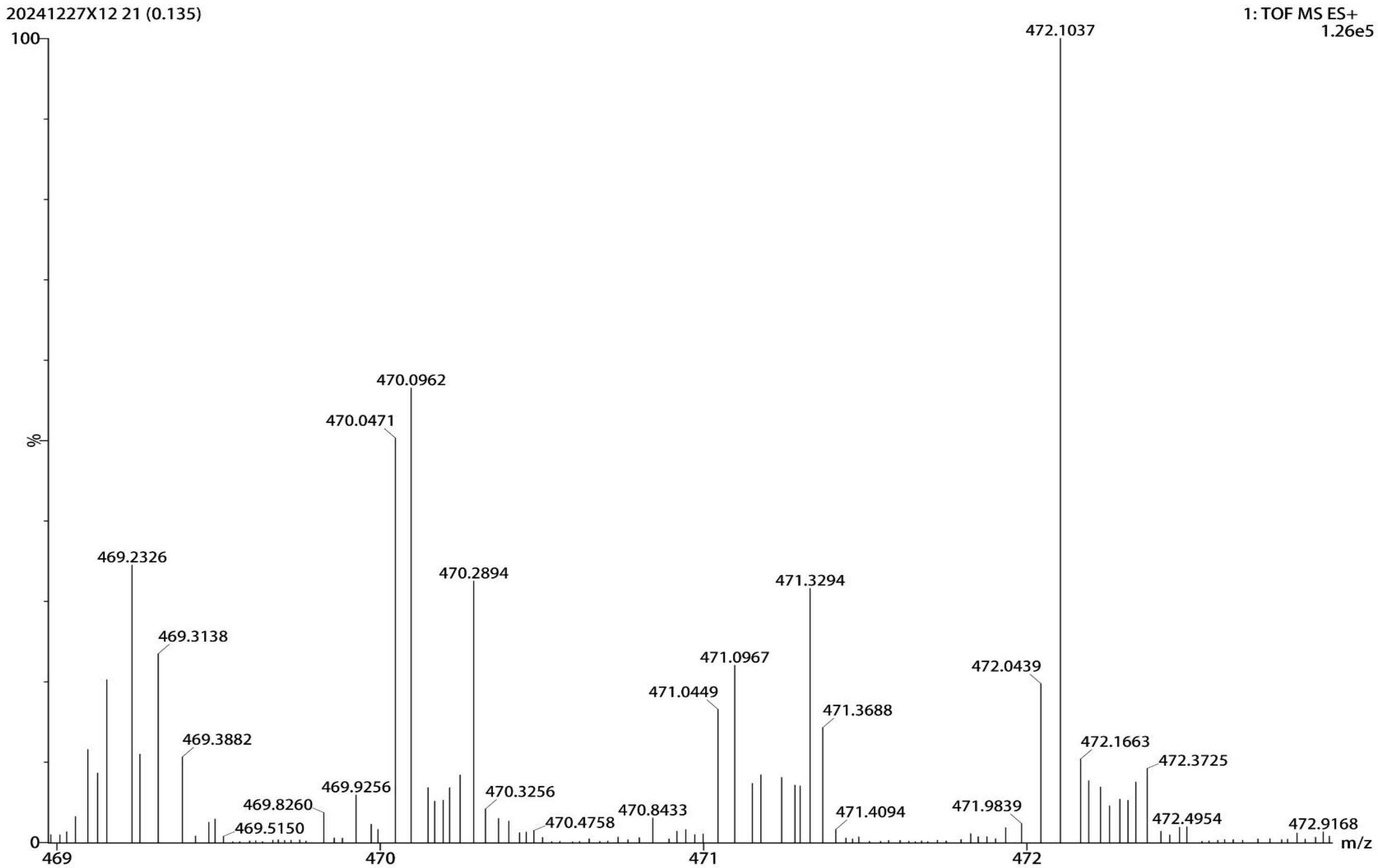

ESI-MS spectrum of the compound X12

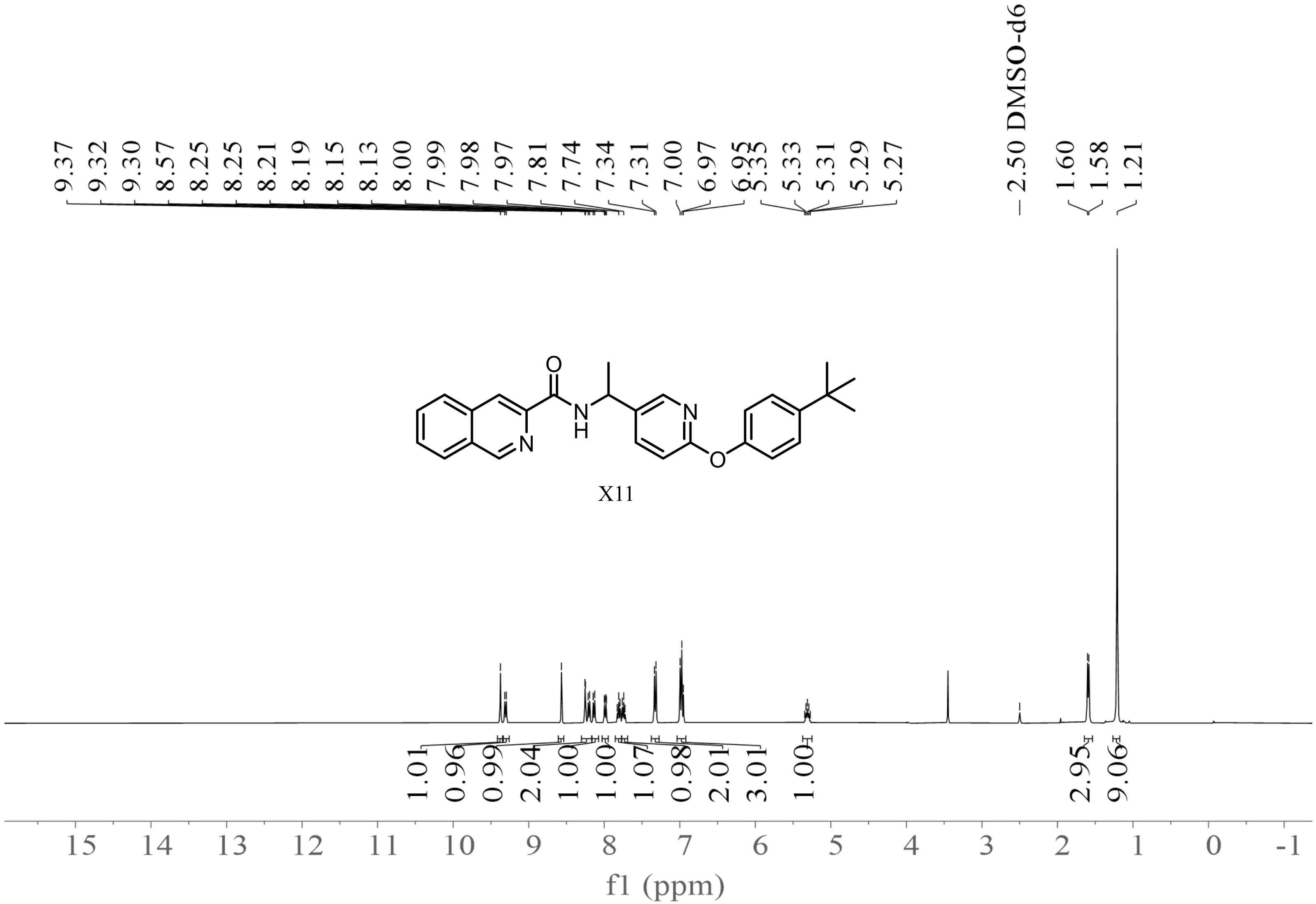

^{1}H NMR spectrum of the compound X11

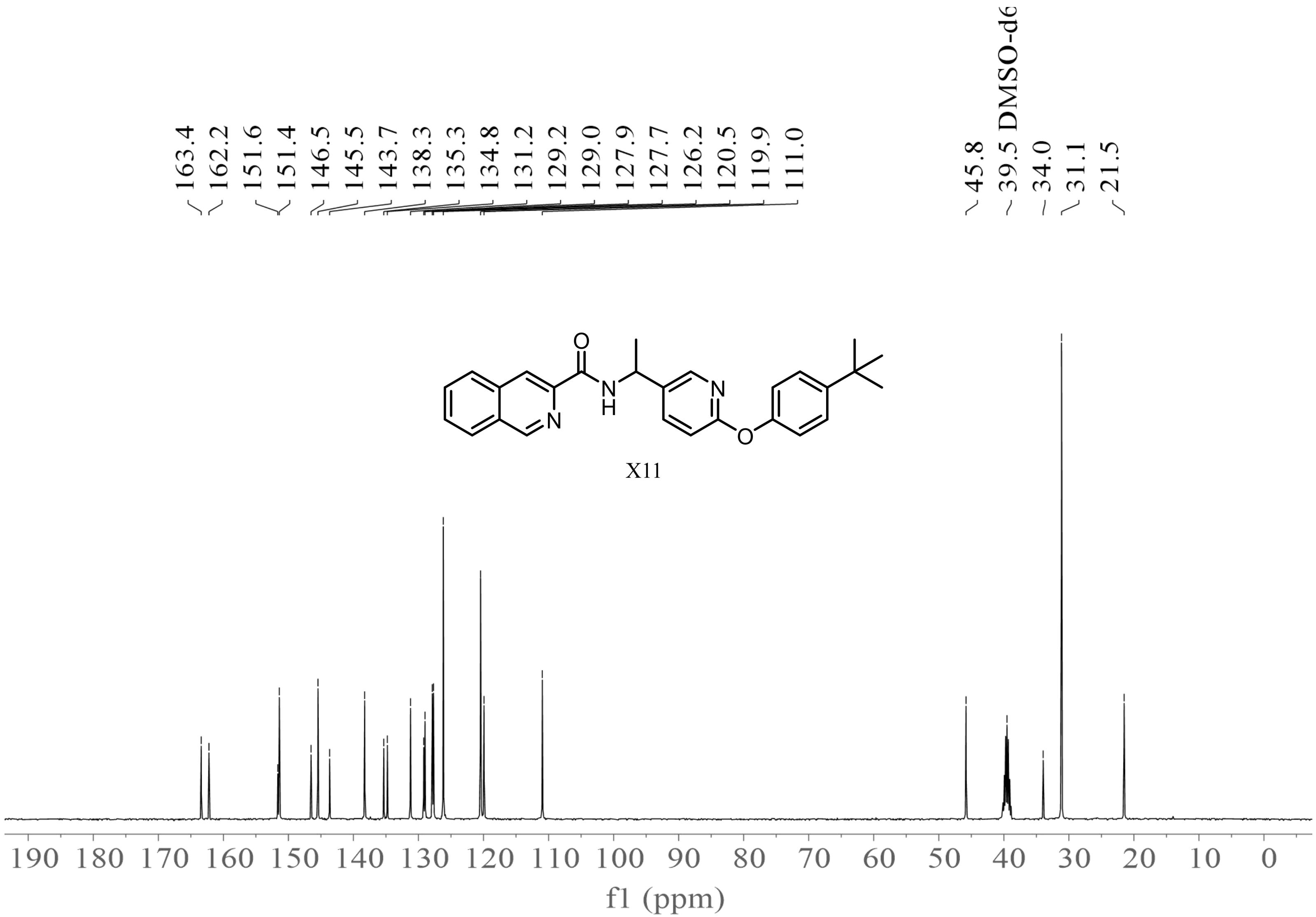

^{13}C NMR spectrum of the compound X11

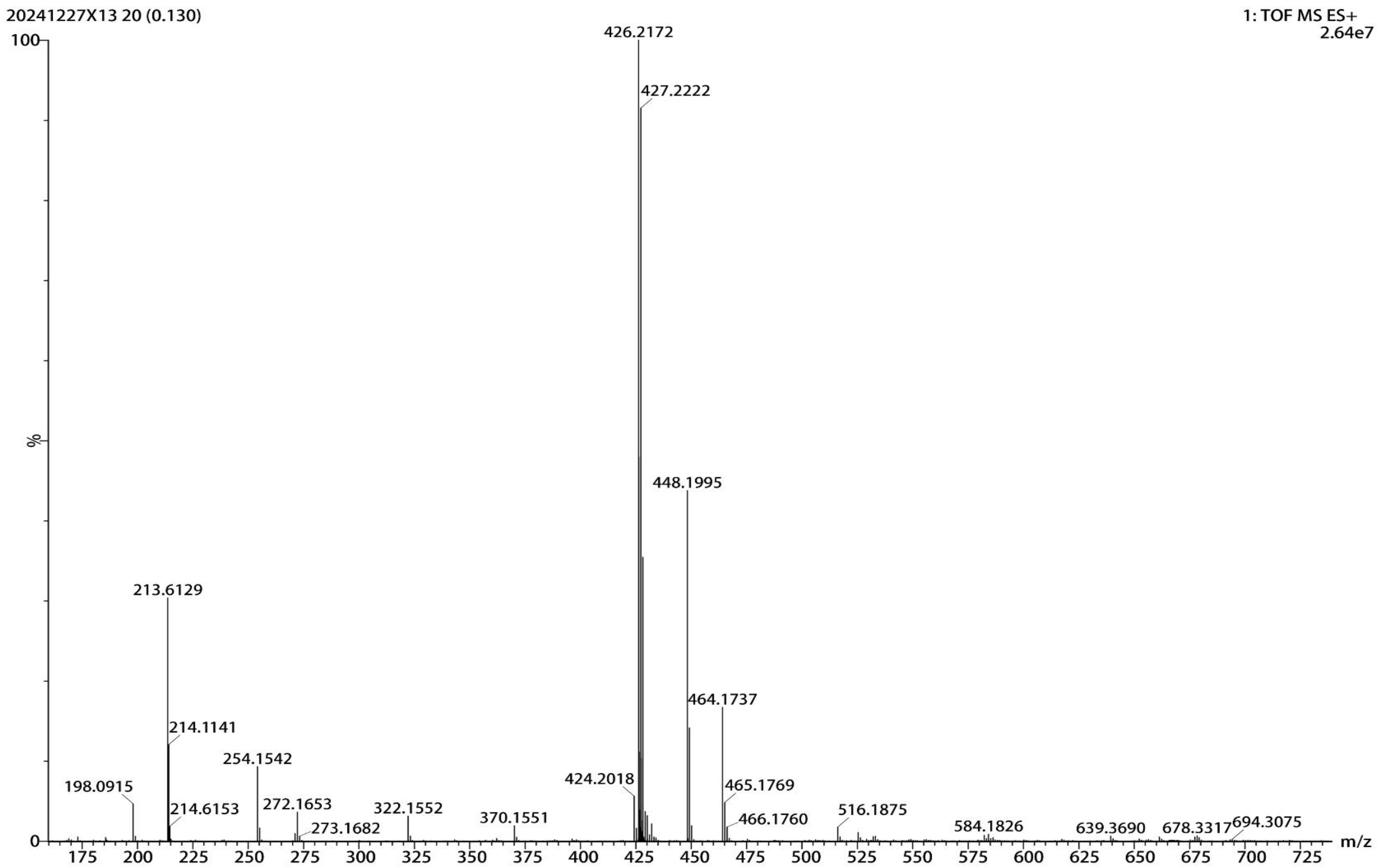

ESI-MS spectrum of the compound X11

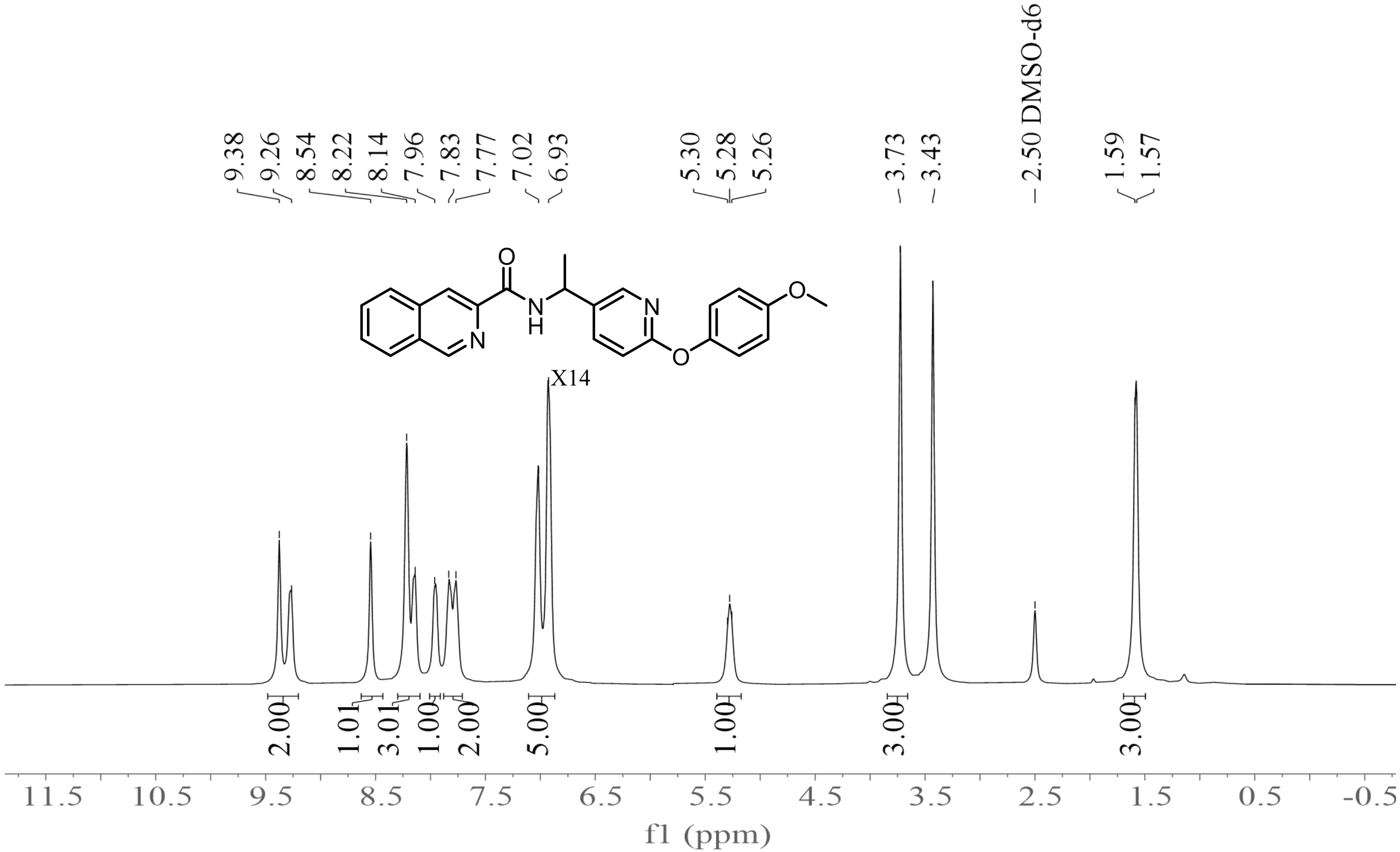

^{1}H NMR spectrum of the compound X14

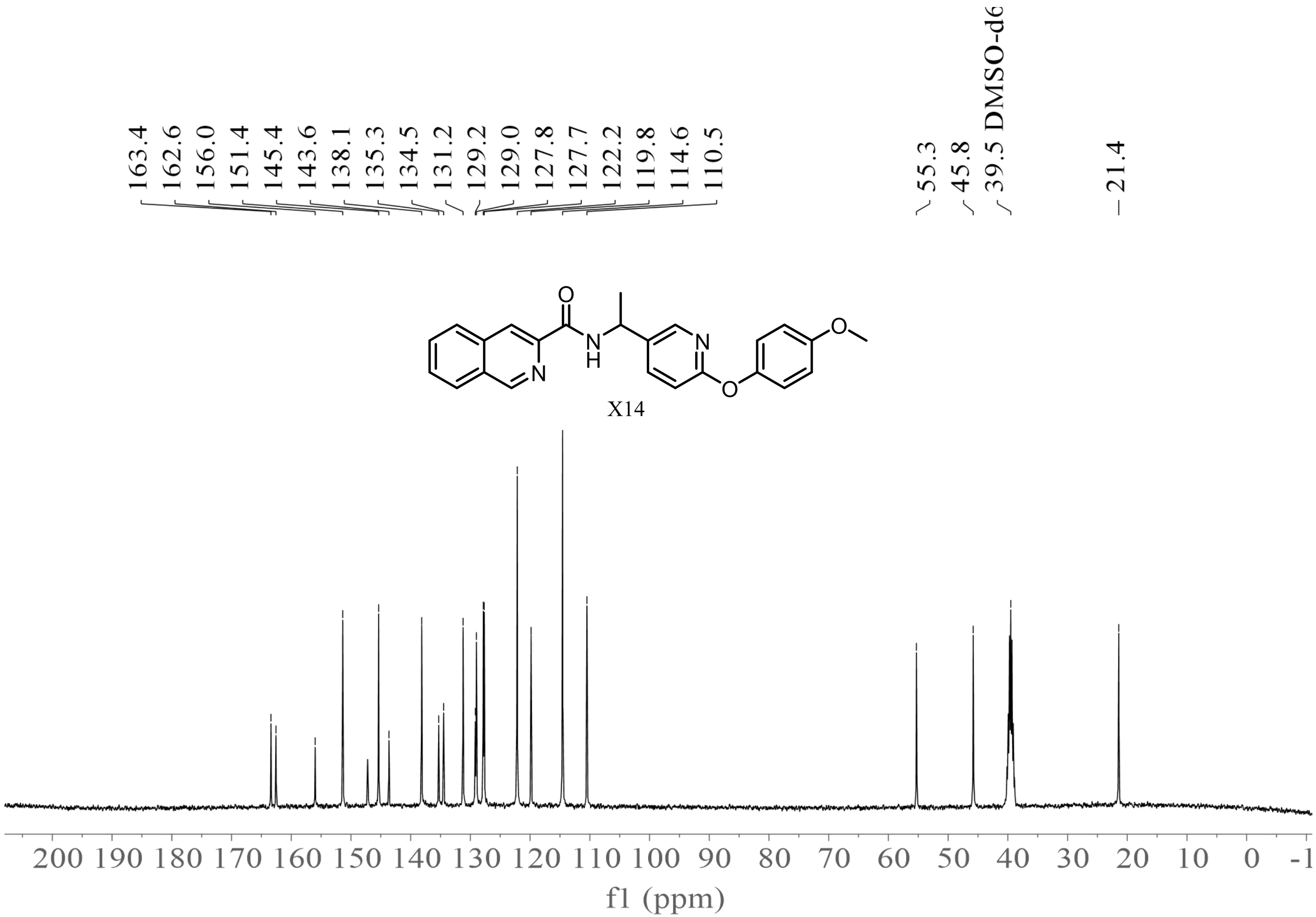

^{13}C NMR spectrum of the compound X14

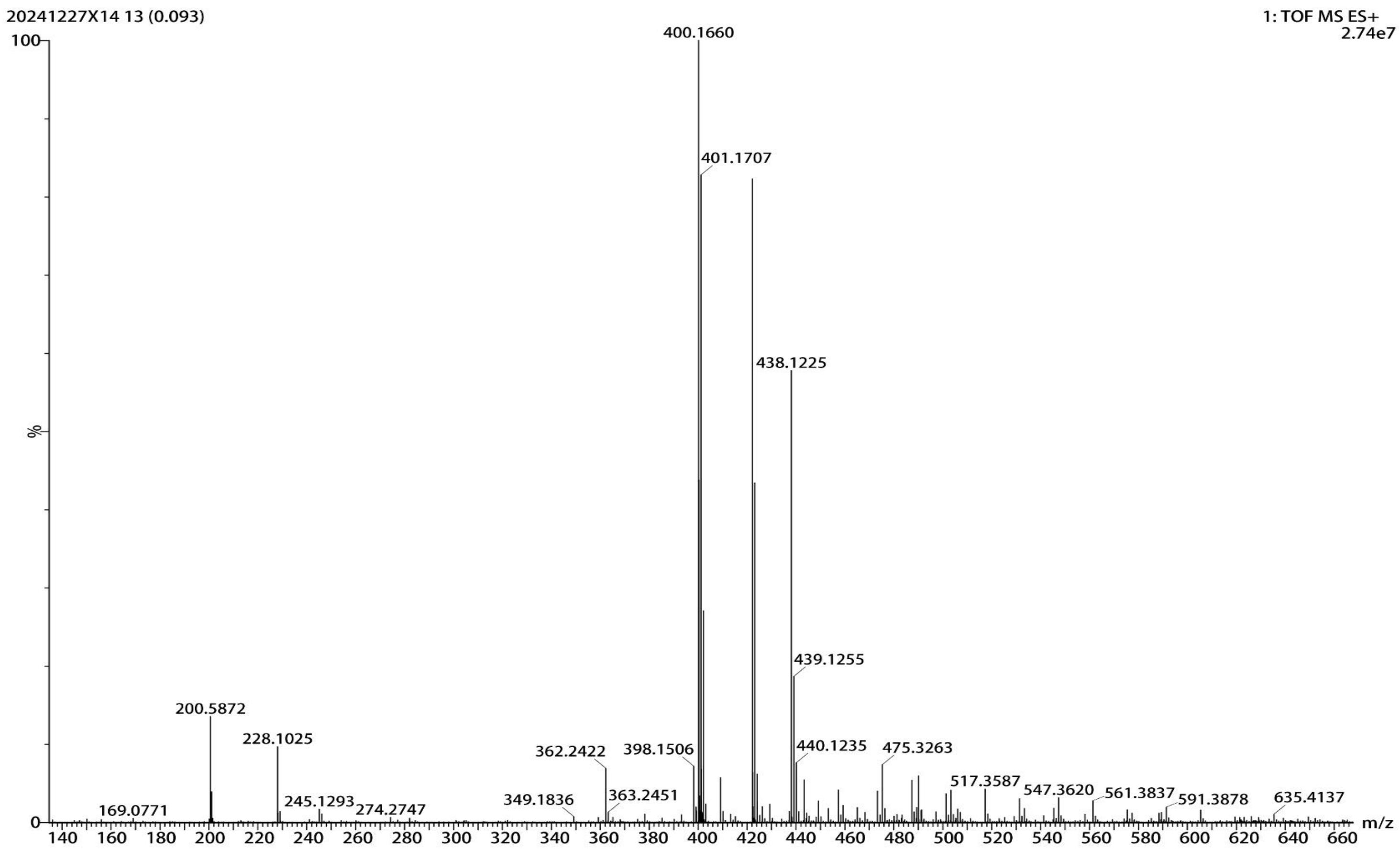

ESI-MS spectrum of the compound X14

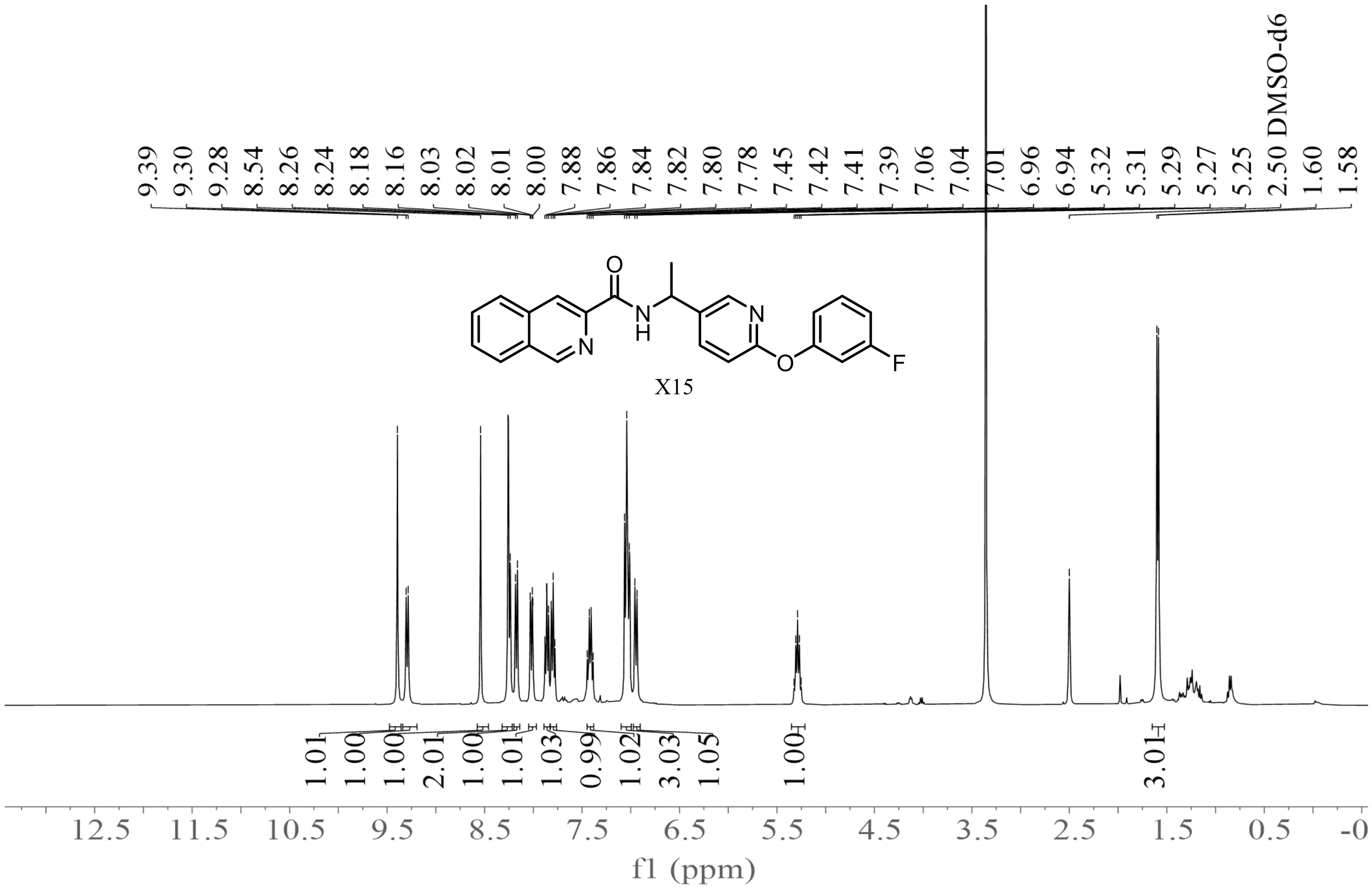

^{1}H NMR spectrum of the compound X15

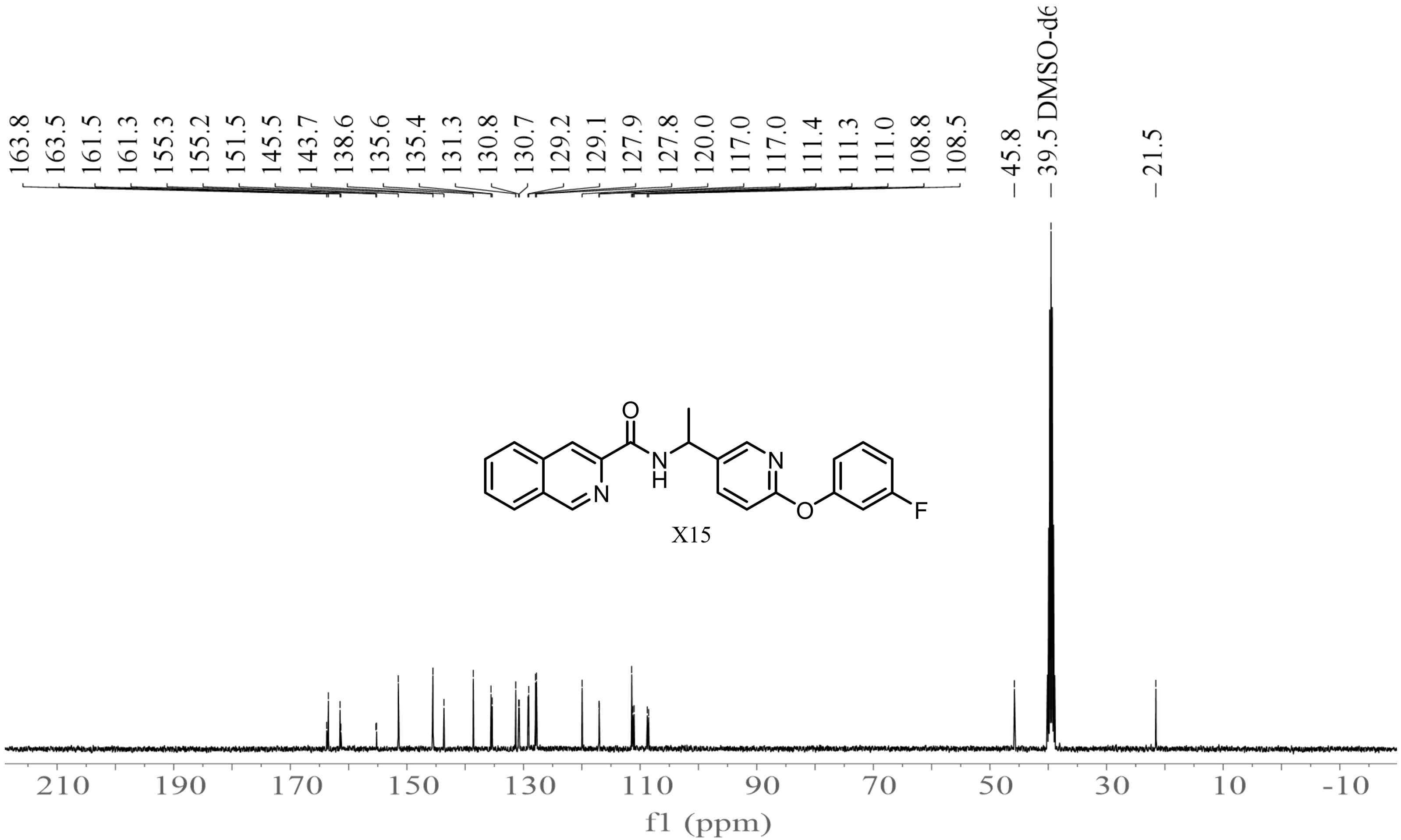

^{13}C NMR spectrum of the compound X15

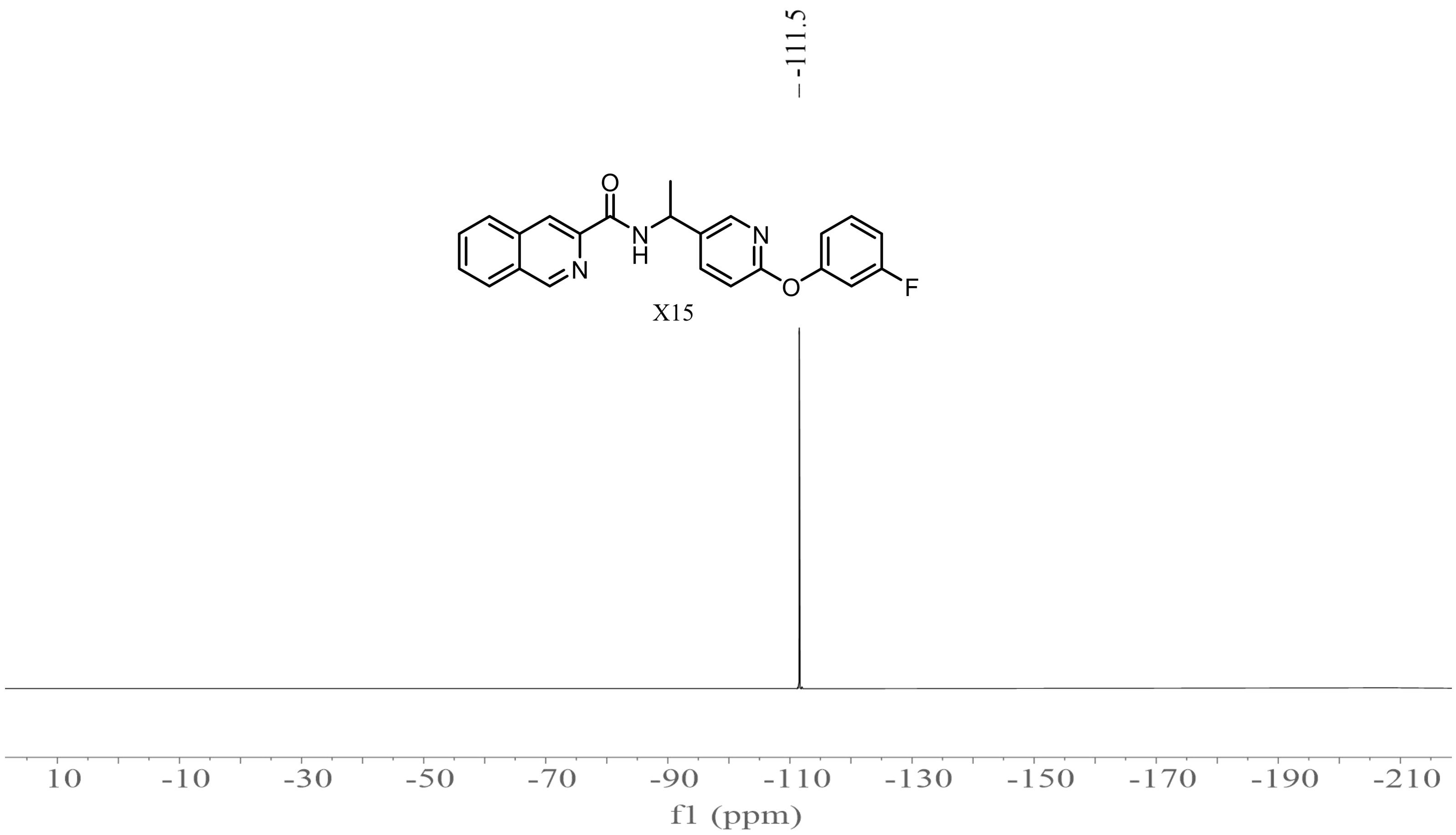

^{19}F NMR spectrum of the compound X15

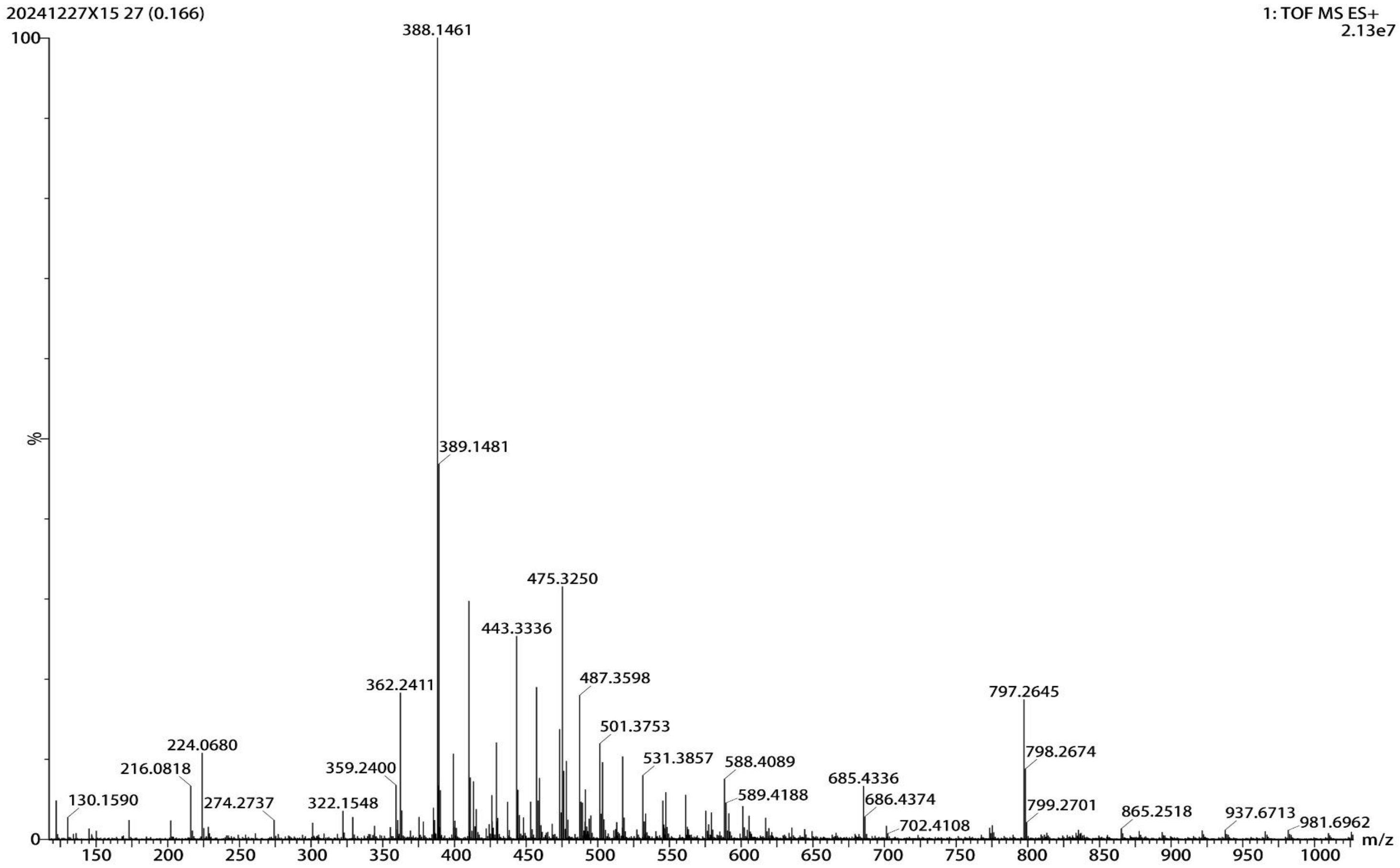

ESI-MS spectrum of the compound X15

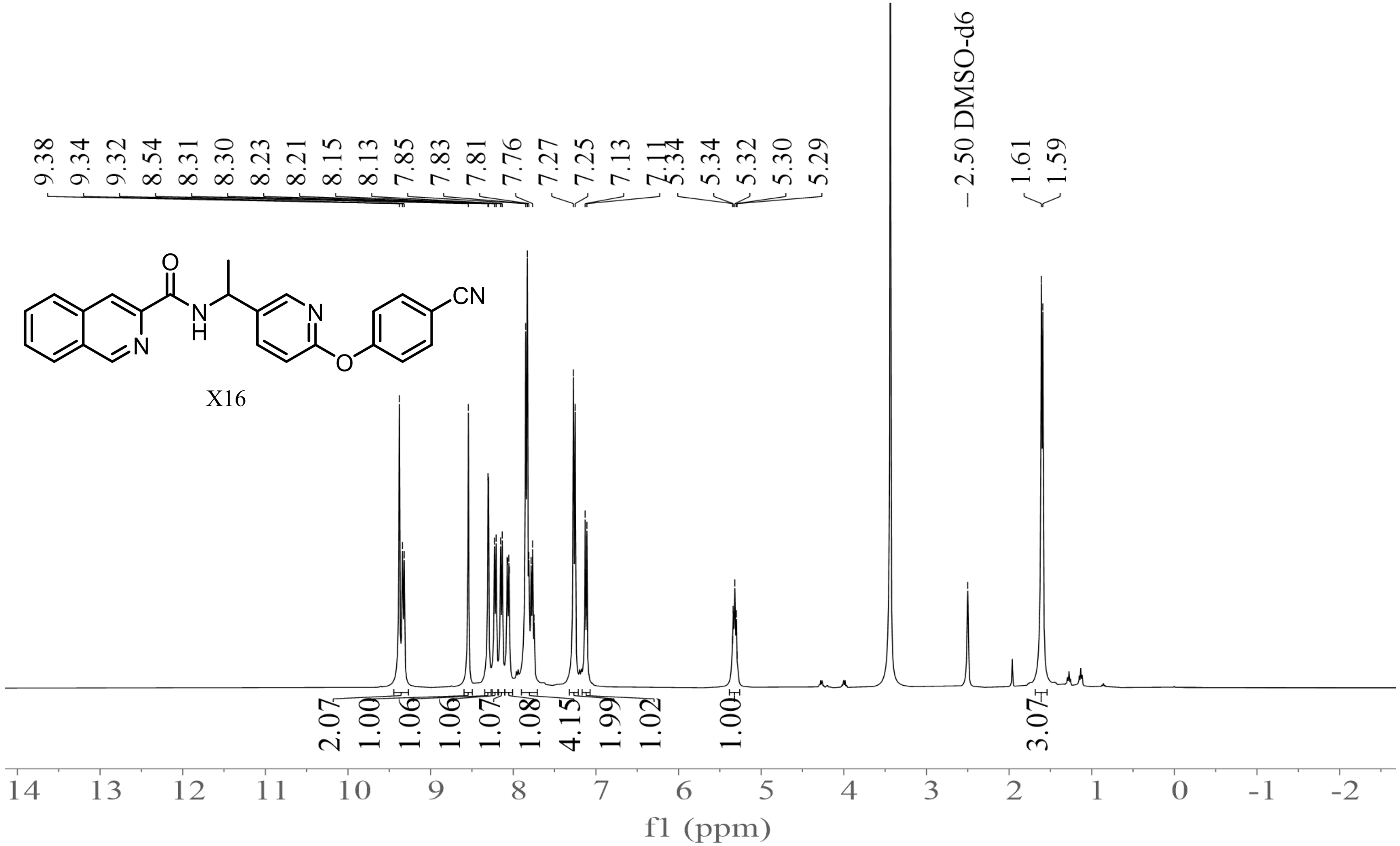

^{1}H NMR spectrum of the compound X16

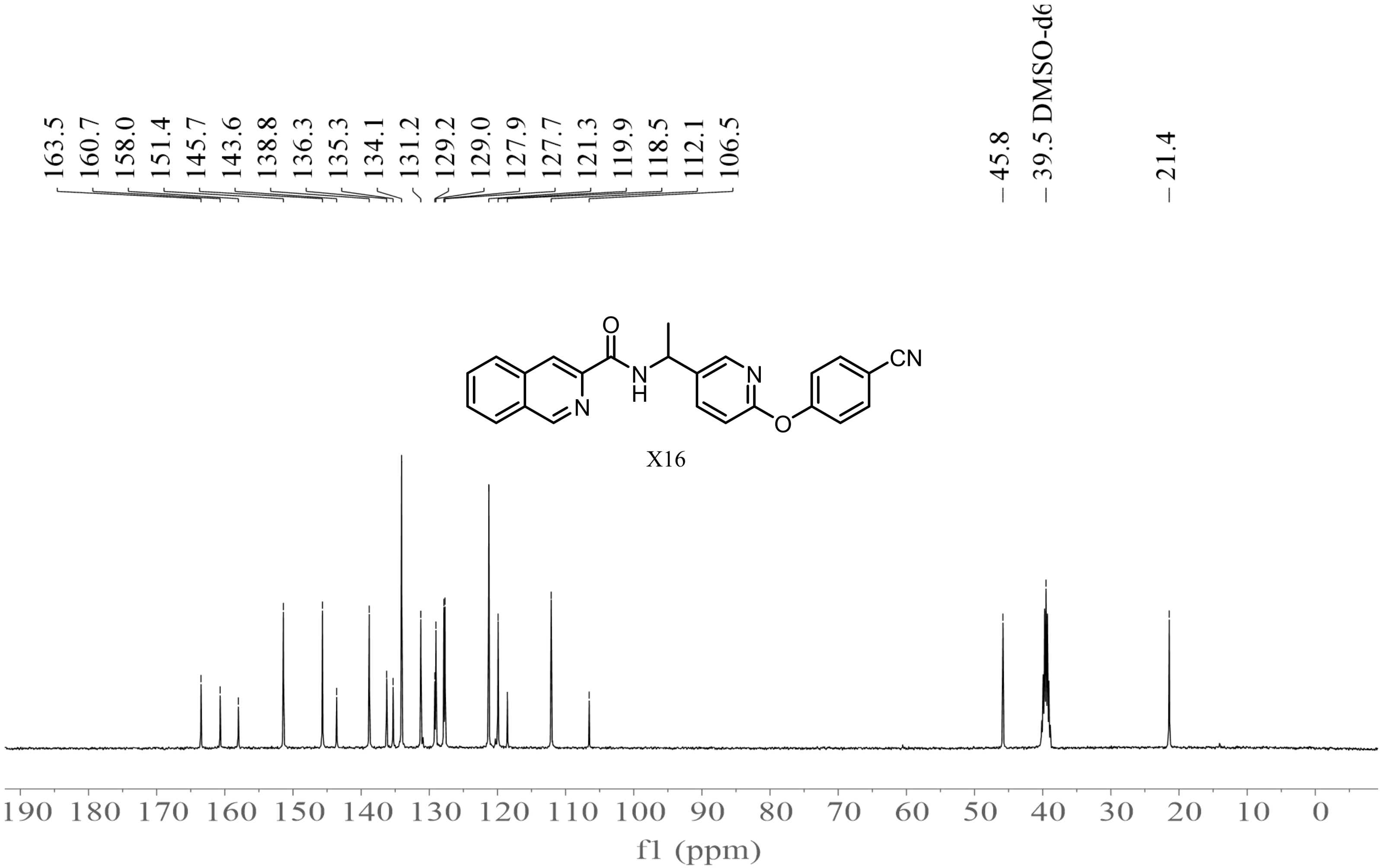

X16

^{13}C NMR spectrum of the compound X16

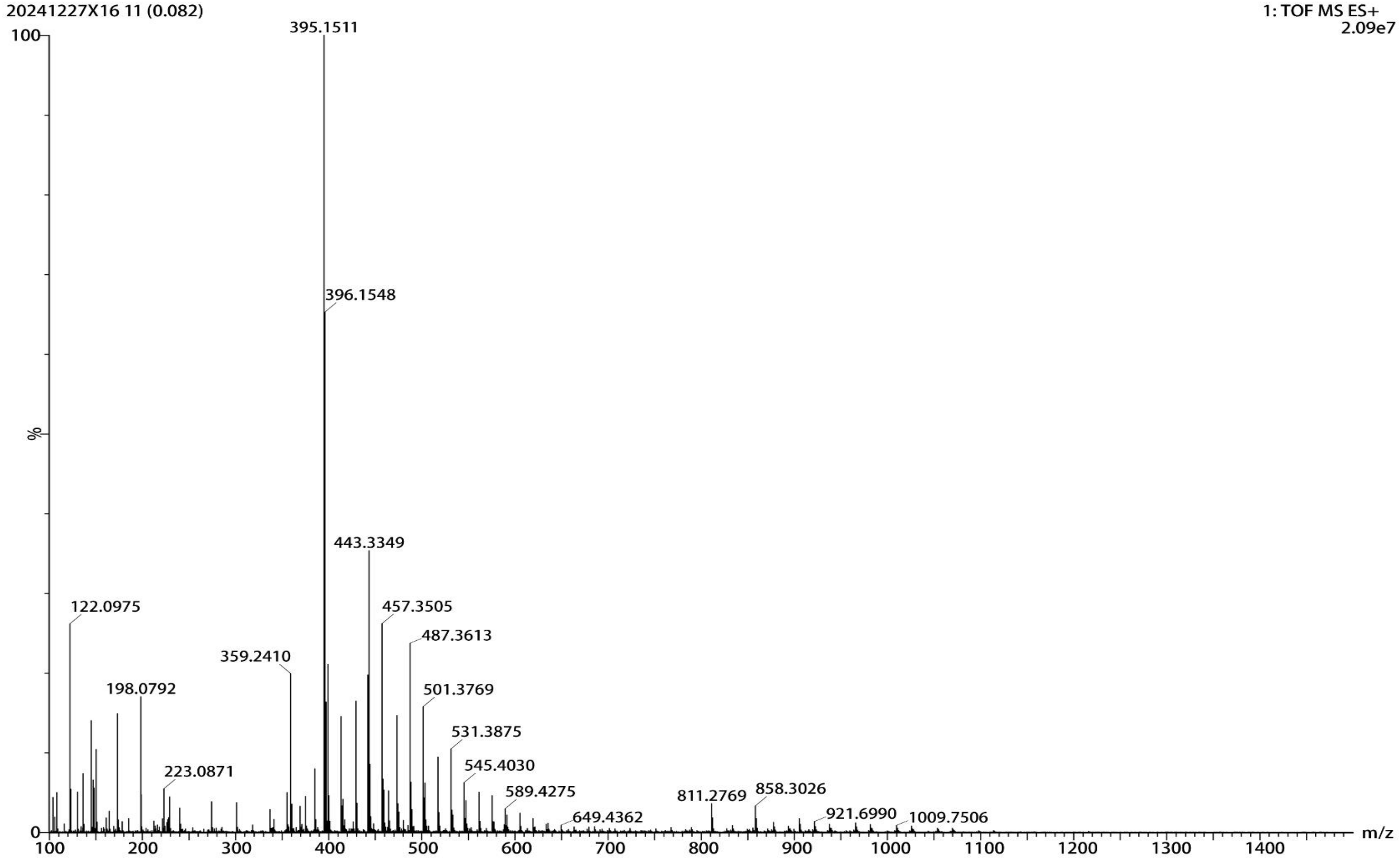

ESI-MS spectrum of the compound X16

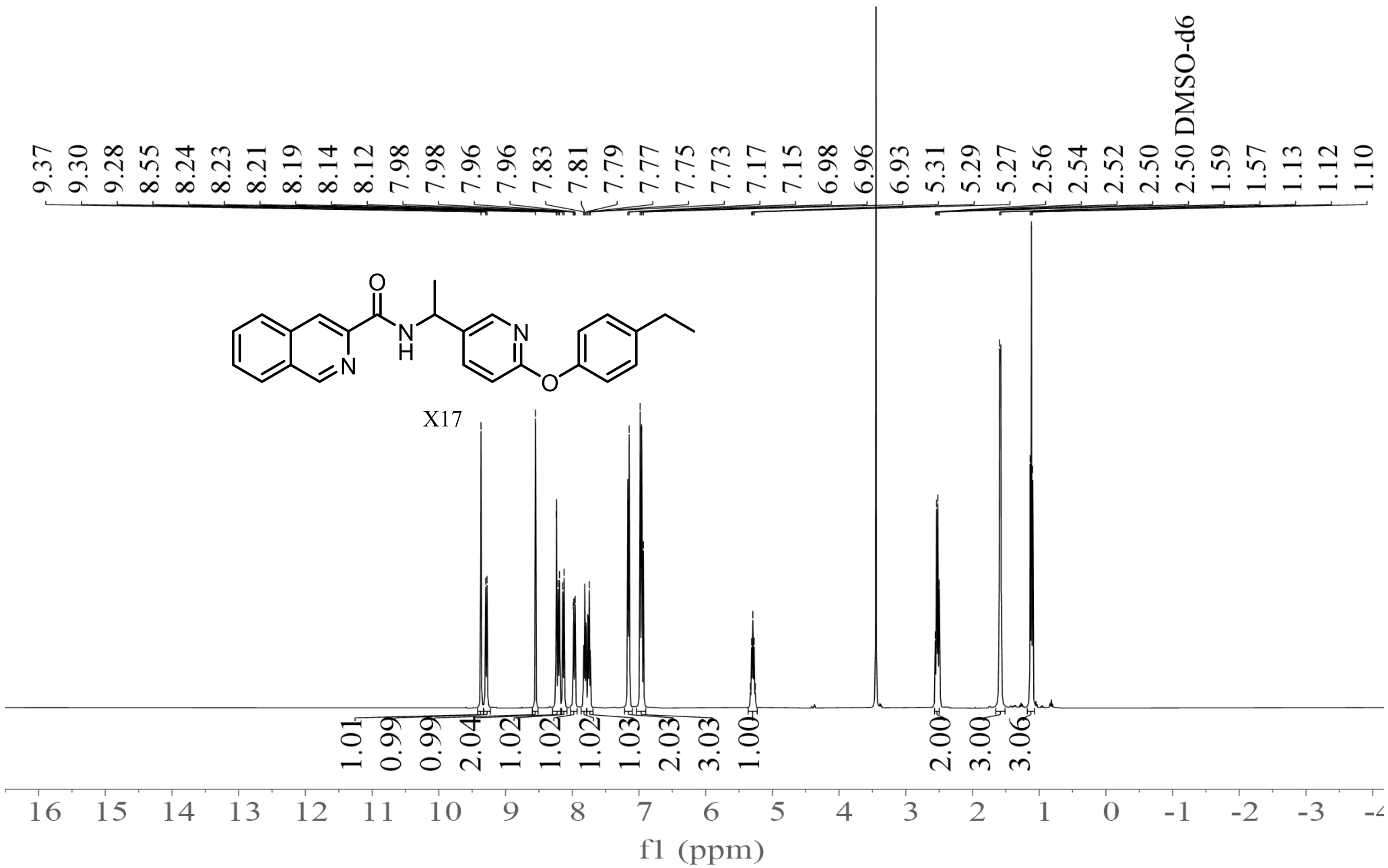

^{1}H NMR spectrum of the compound X17

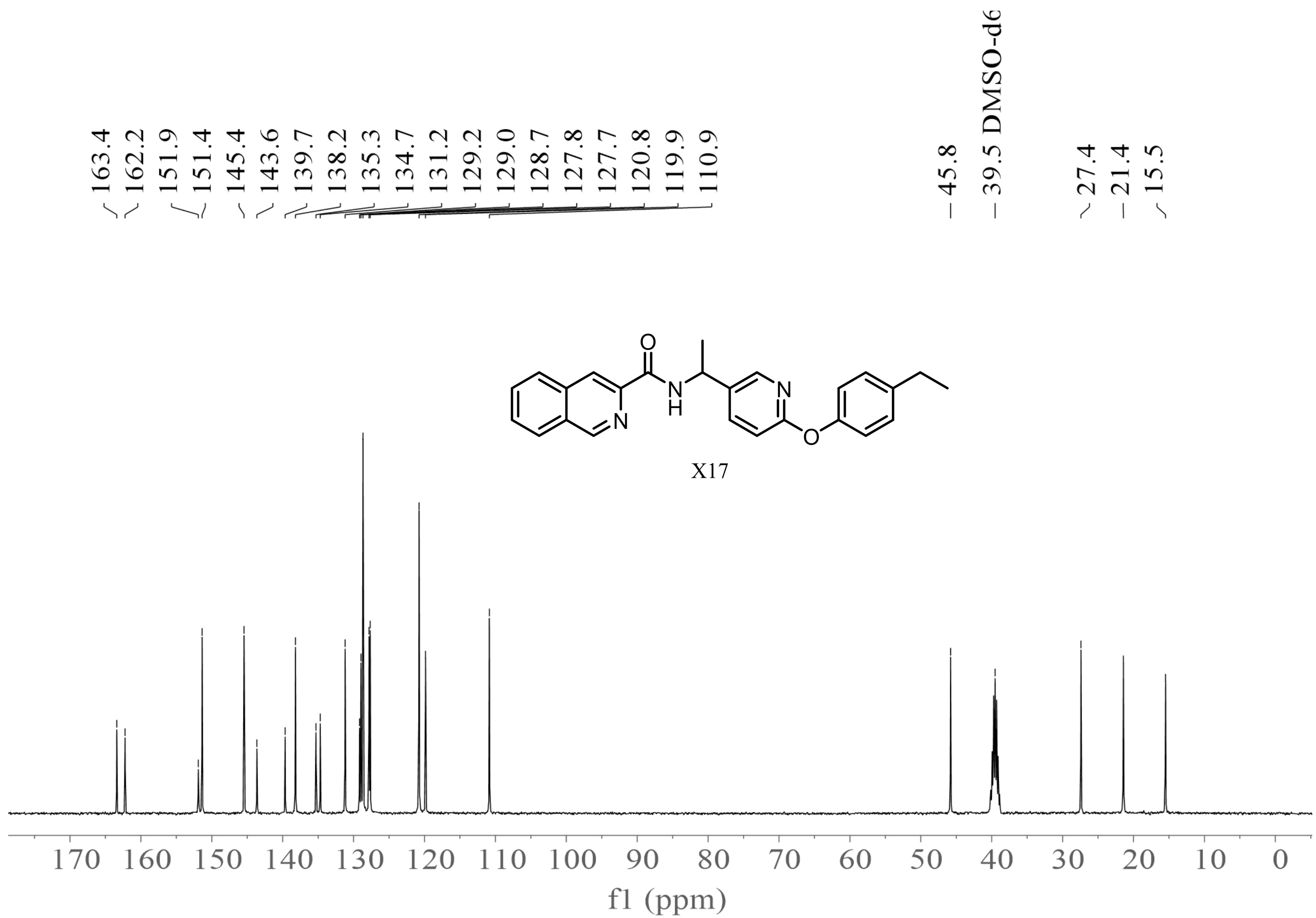

^{13}C NMR spectrum of the compound X17

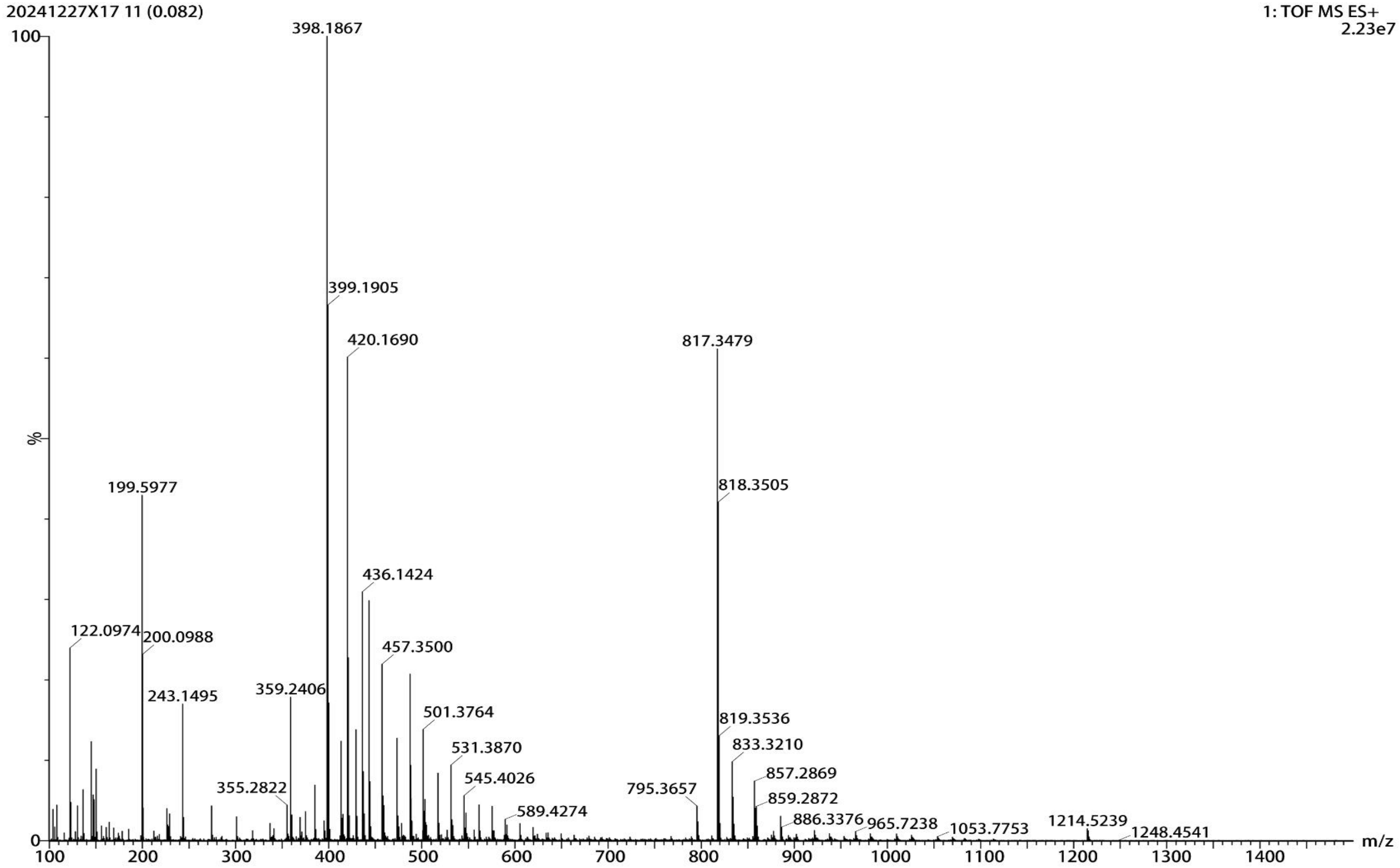

ESI-MS spectrum of the compound X17

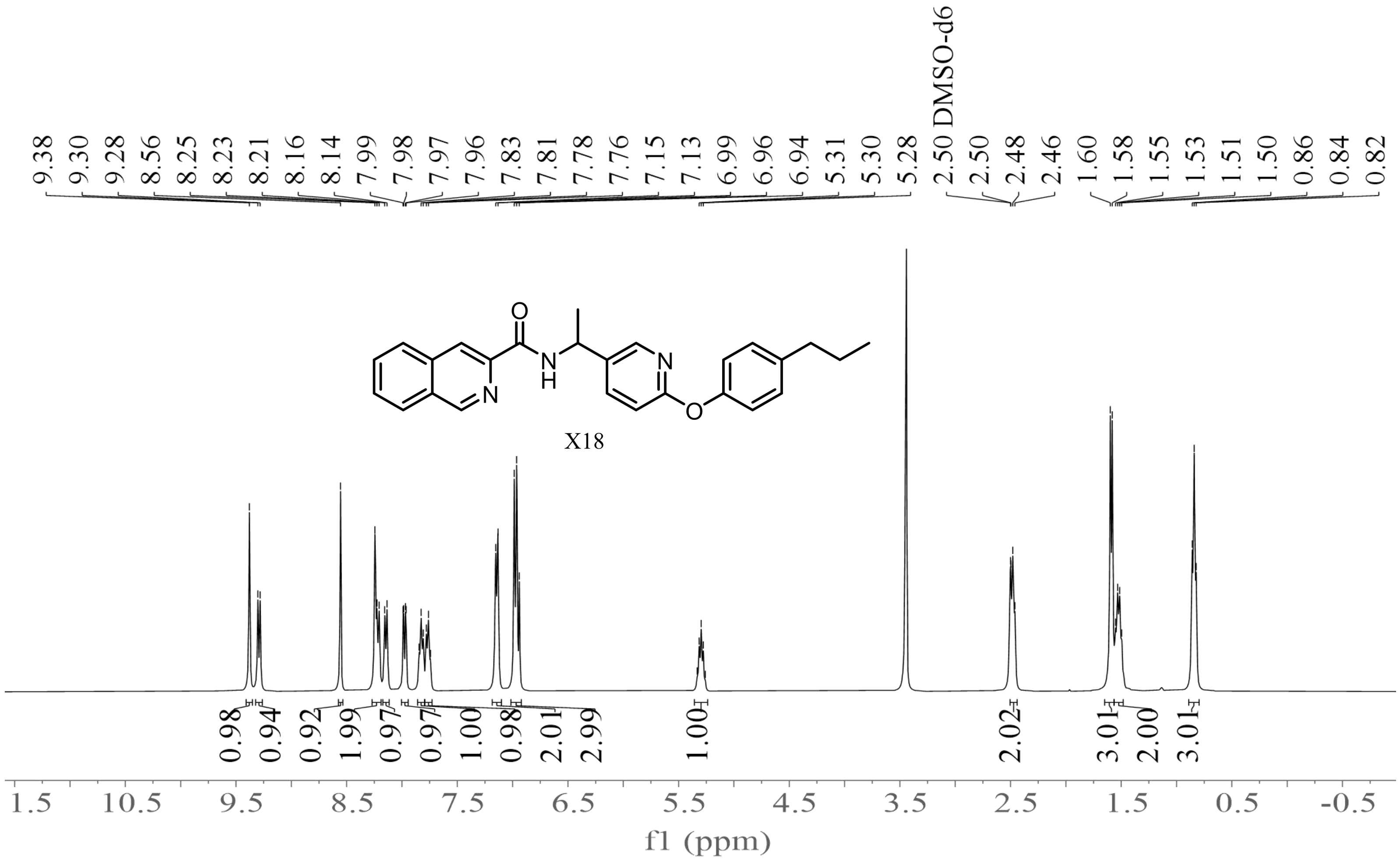

^{1}H NMR spectrum of the compound X18

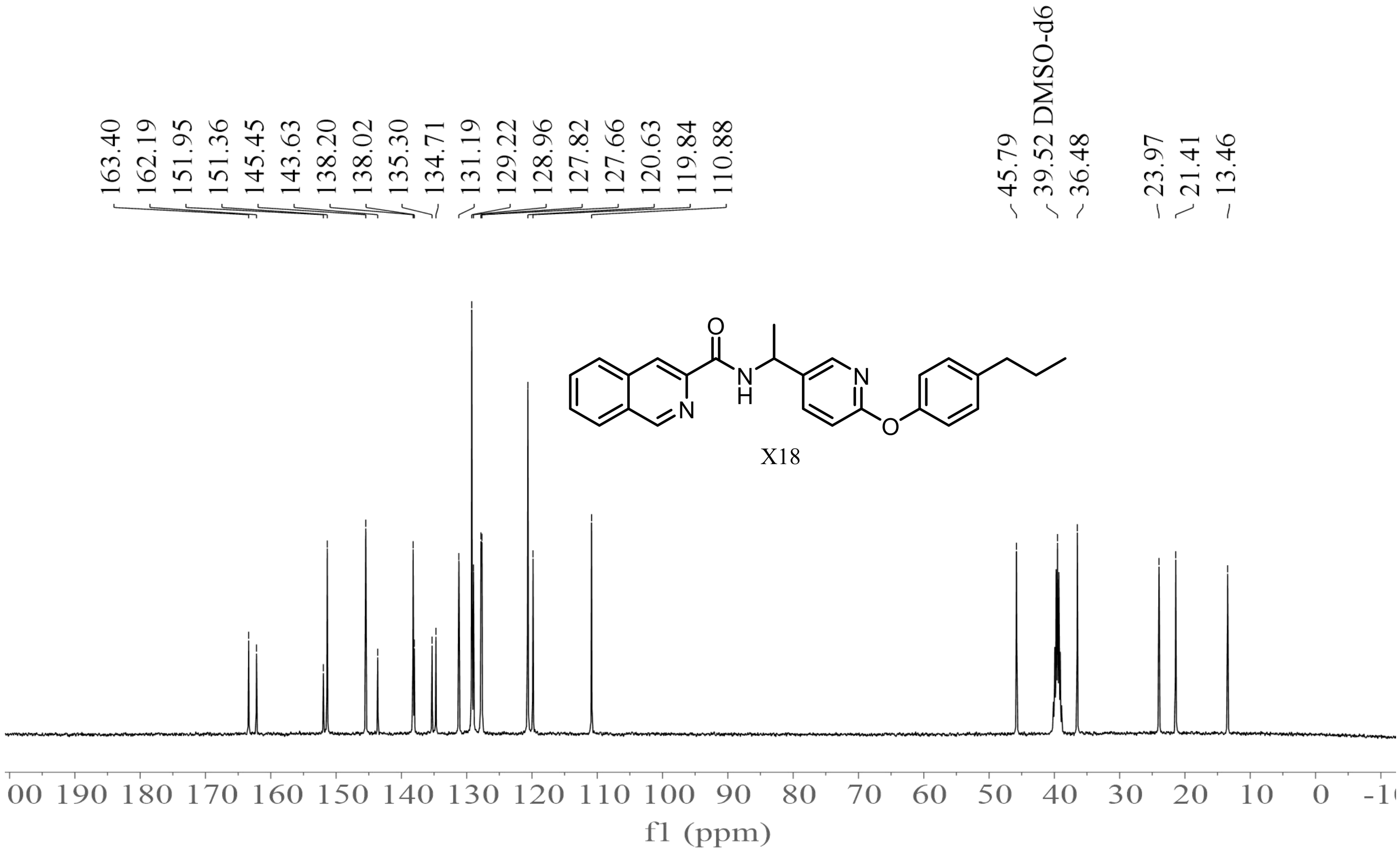

^{13}C NMR spectrum of the compound X18

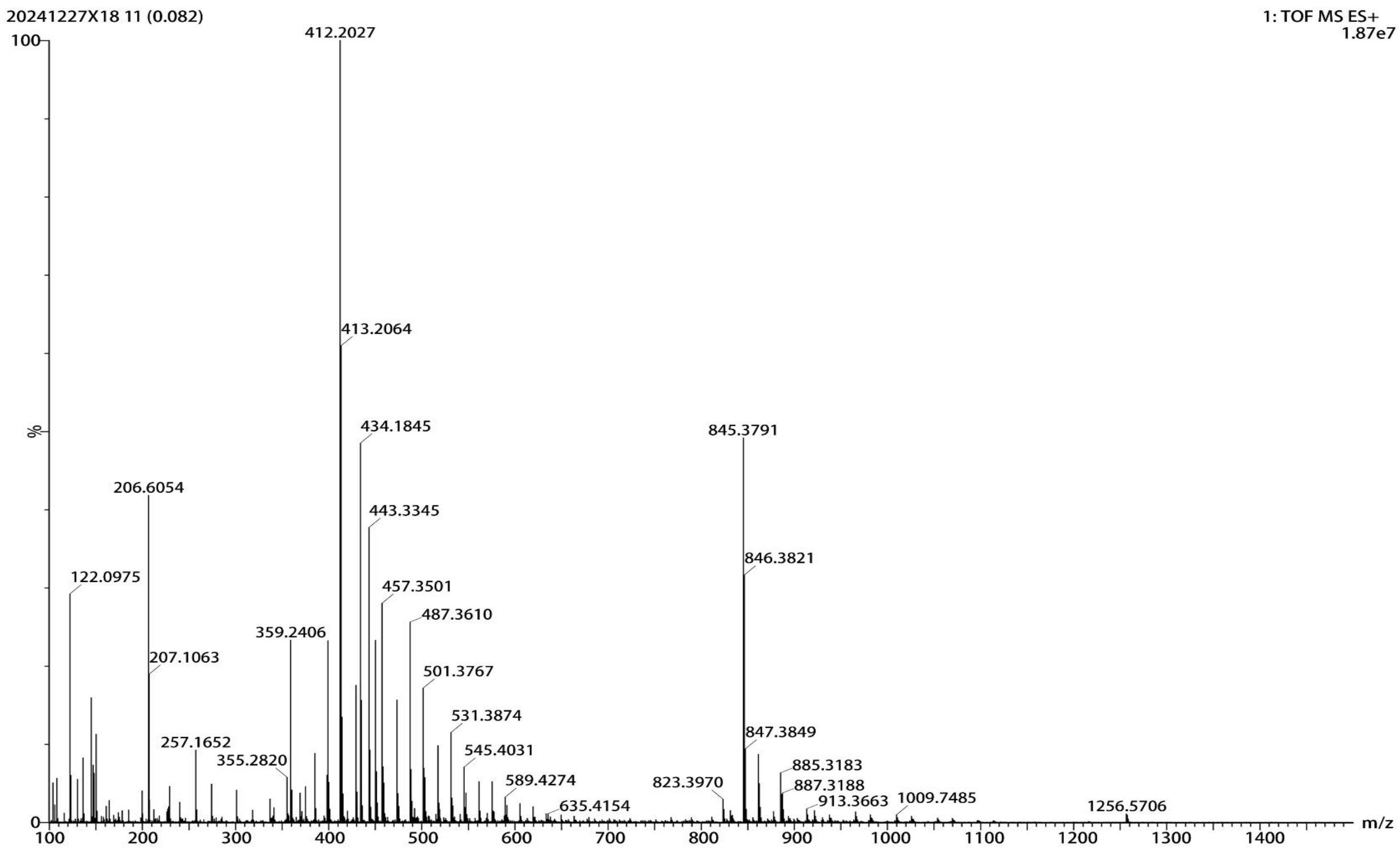

ESI-MS spectrum of the compound X18

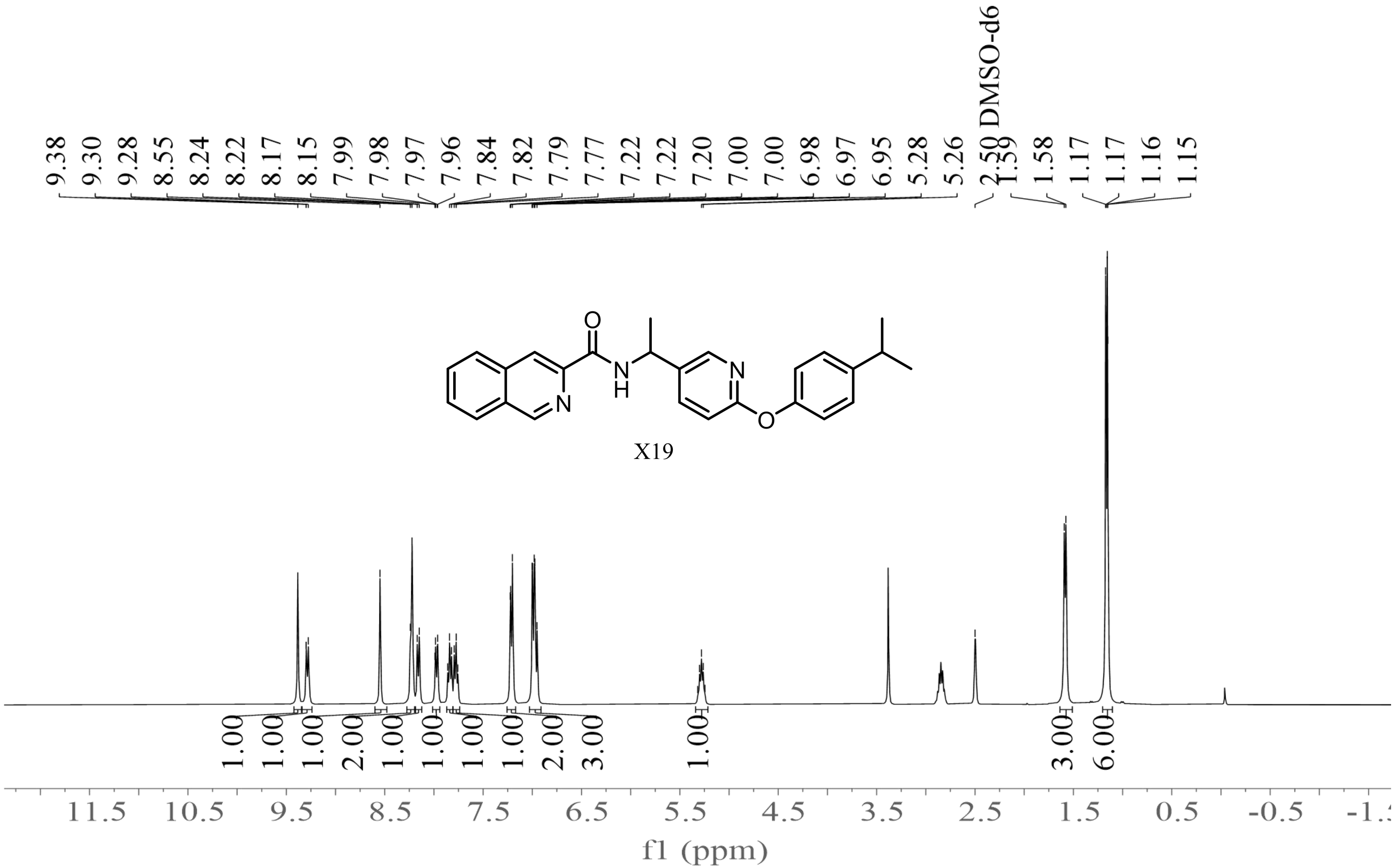

^{1}H NMR spectrum of the compound X19

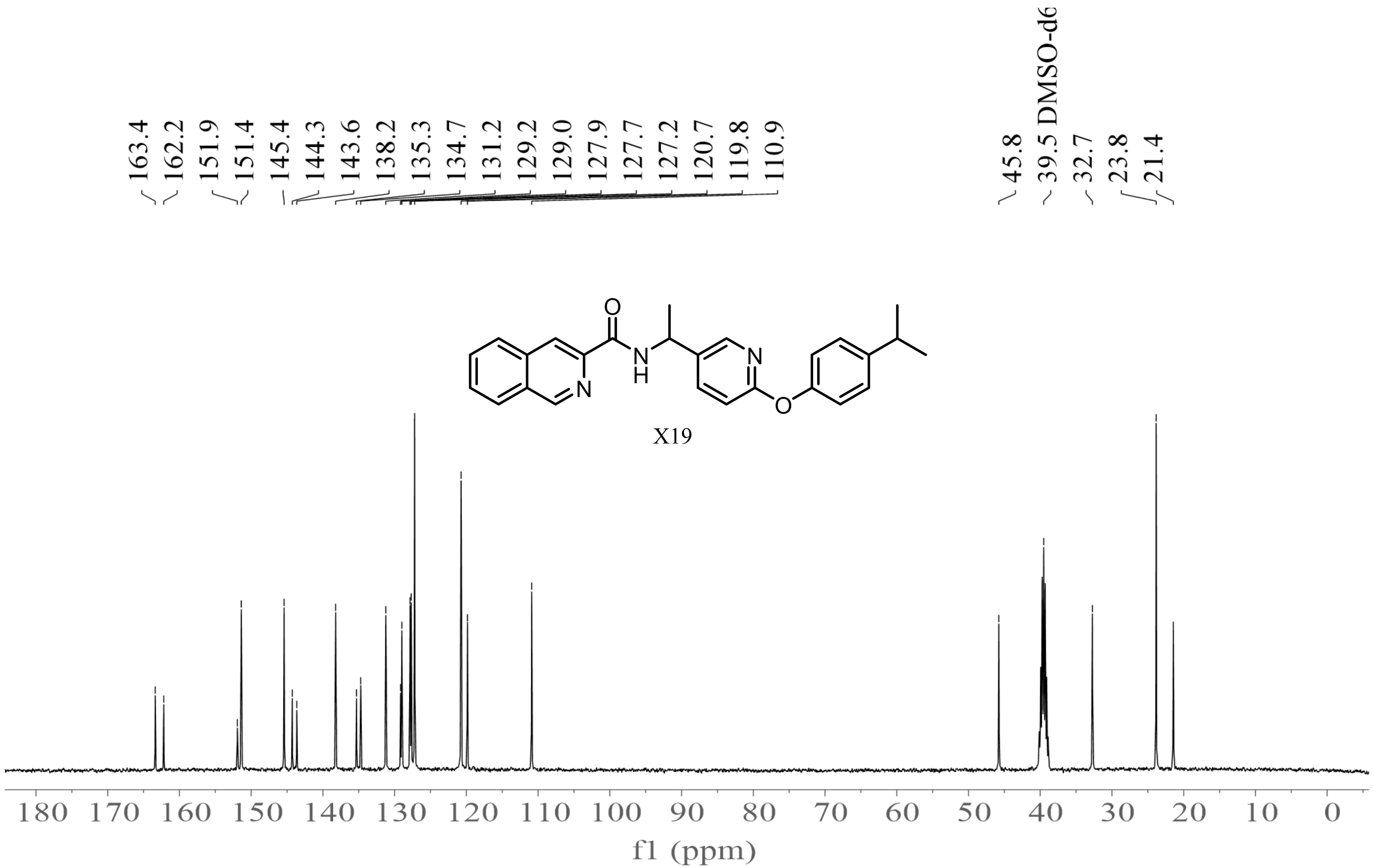

^{13}C NMR spectrum of the compound X19

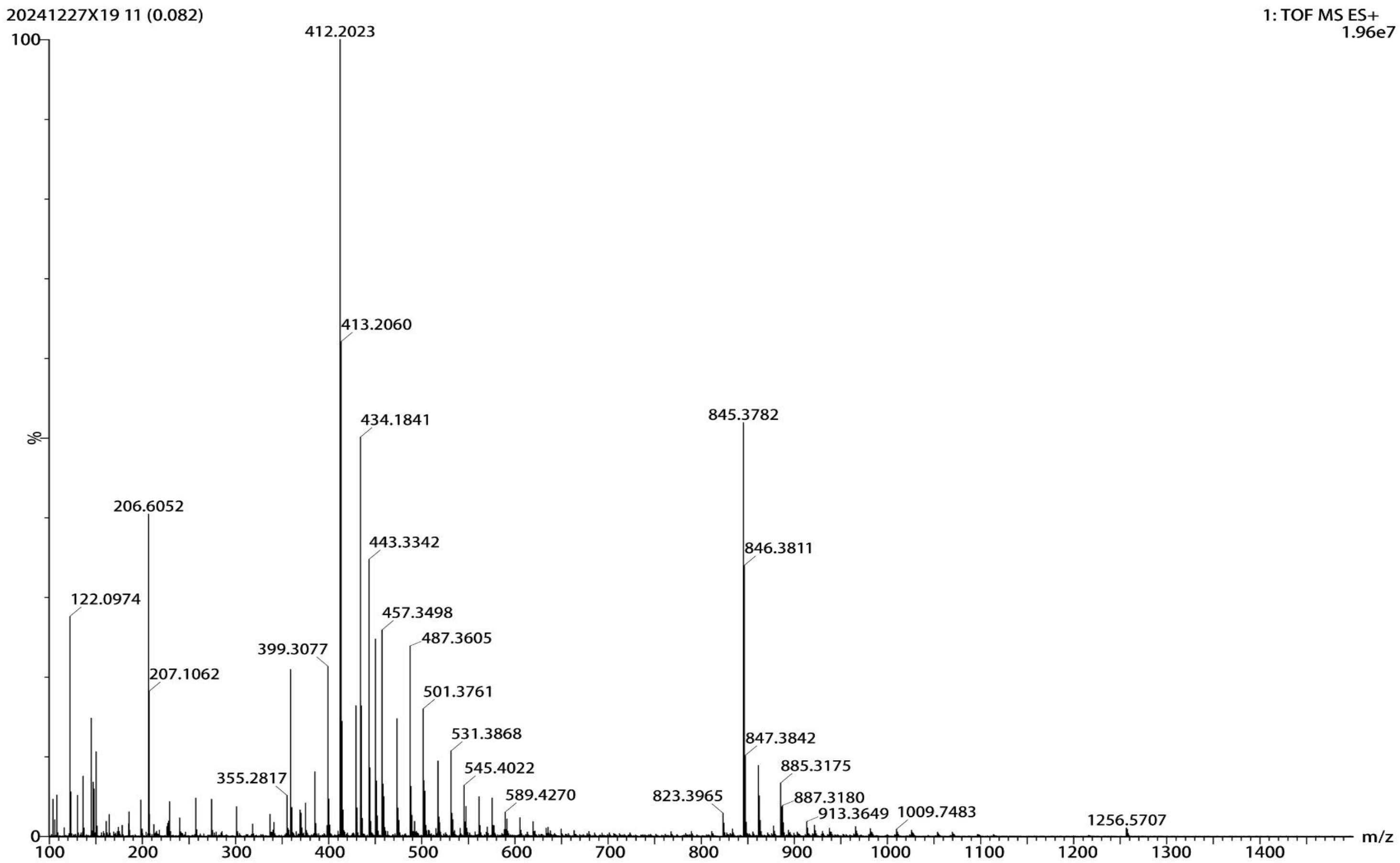

ESI-MS spectrum of the compound X19

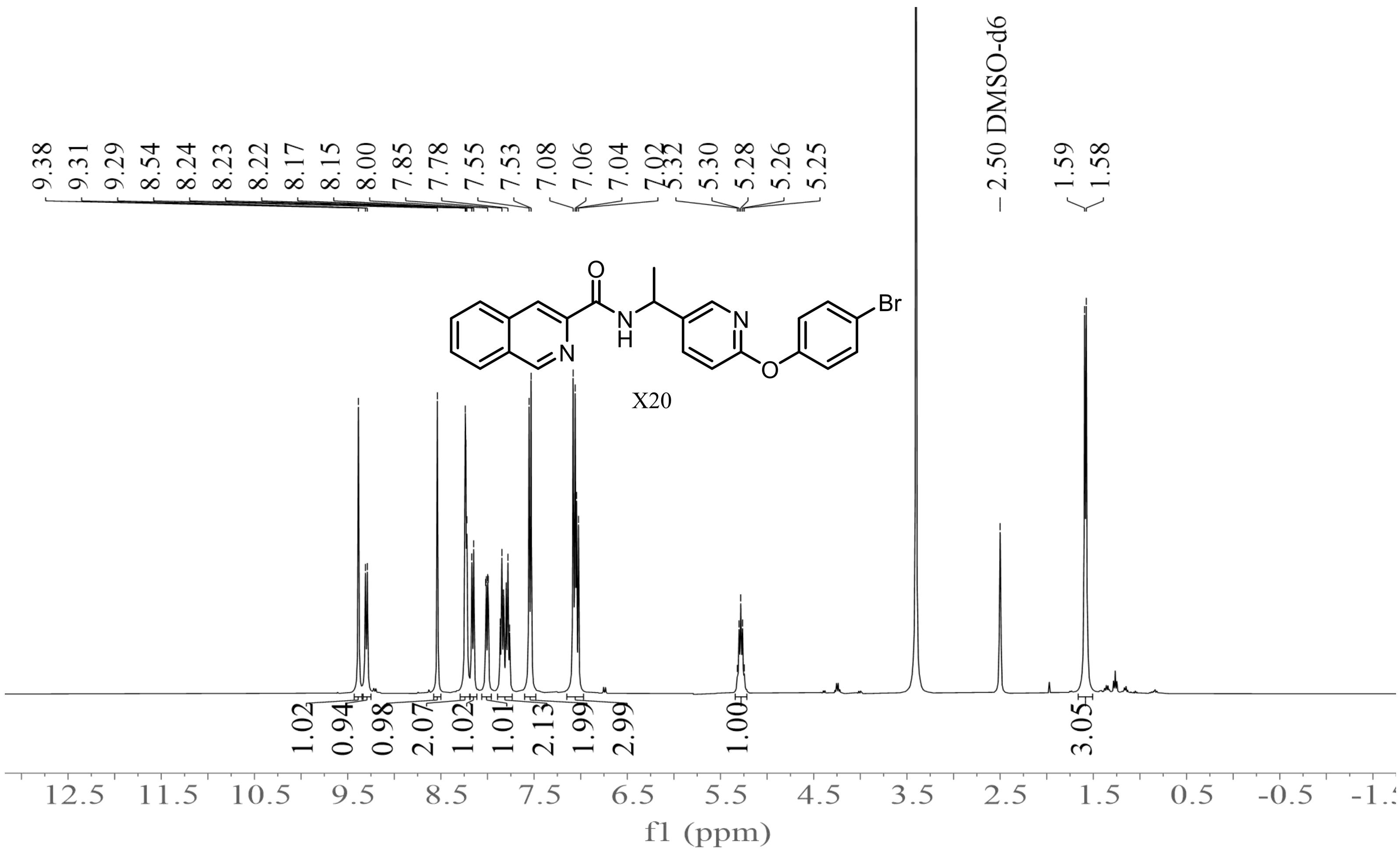

^{1}H NMR spectrum of the compound X20

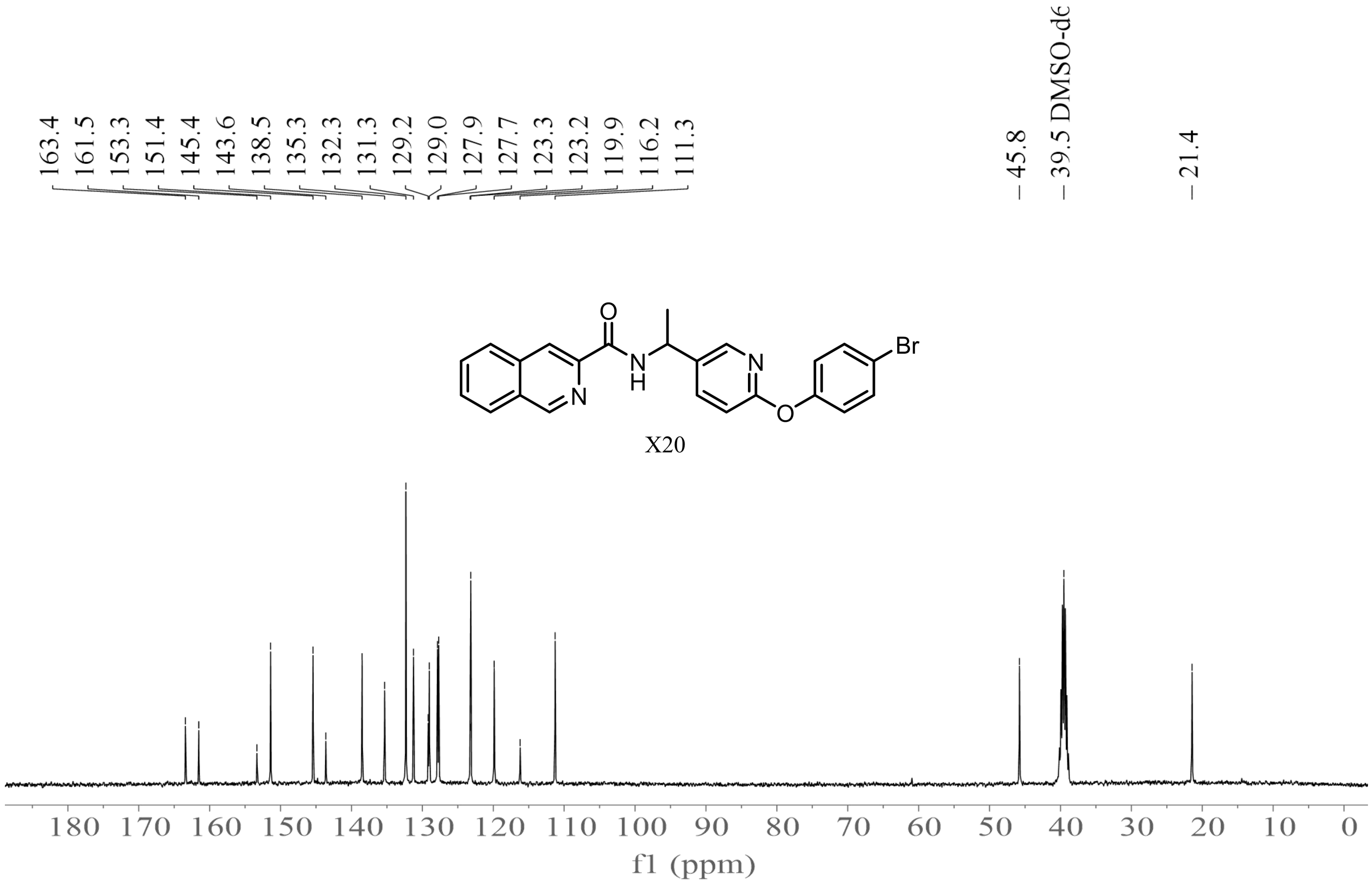

^{13}C NMR spectrum of the compound X20

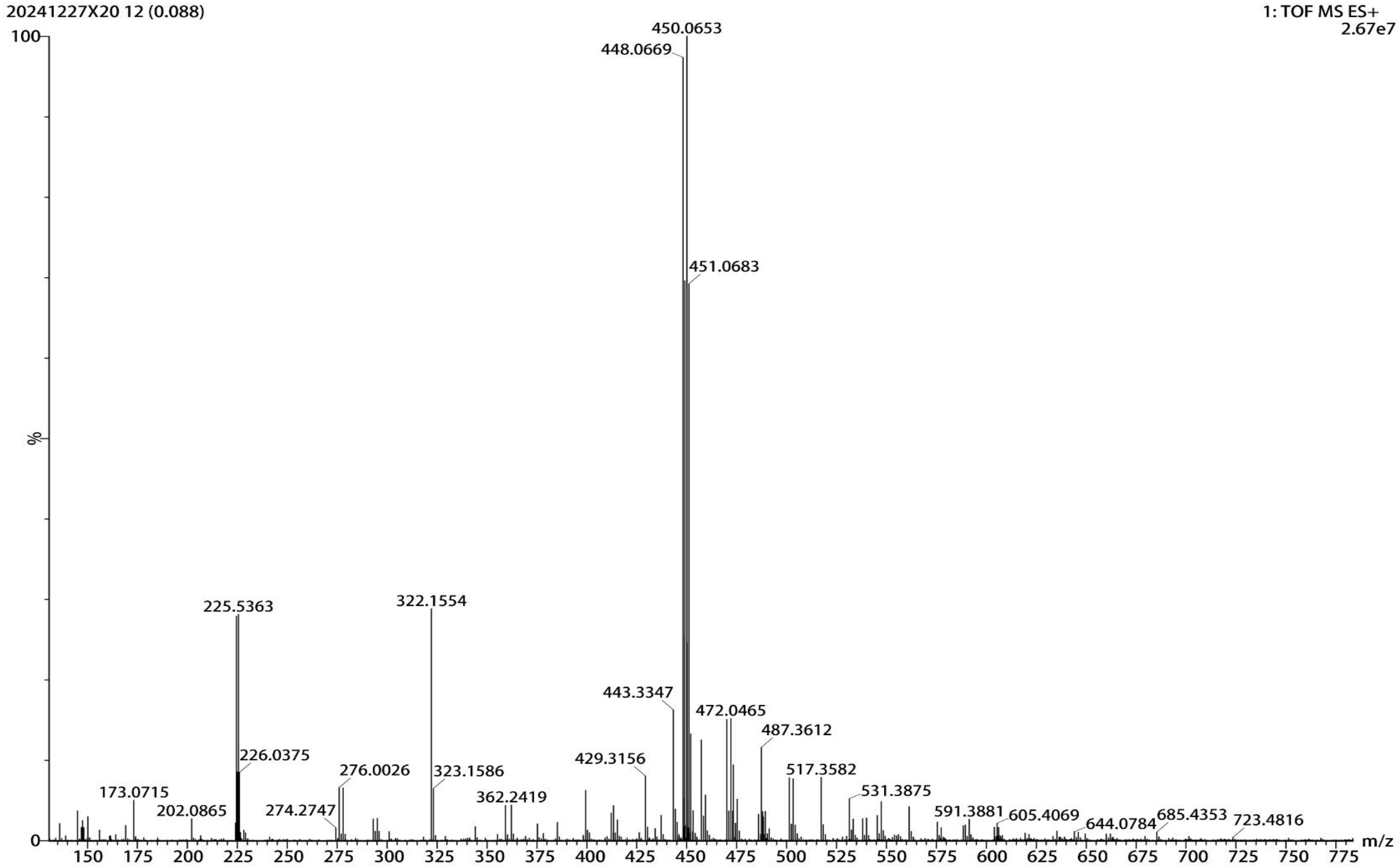

ESI-MS spectrum of the compound X8
